# Linc00707 regulates autophagy and promotes the progression of triple negative breast cancer by activation of PI3K/AKT/mTOR pathway

**DOI:** 10.1038/s41420-024-01906-7

**Published:** 2024-03-14

**Authors:** Hongli Li, Qinghua Liu, Yaqiong Hu, Chonggao Yin, Yunxiang Zhang, Peng Gao

**Affiliations:** 1 Department of Pathology, Qi Lu Hospital and School of Basic Medical Sciences, Shandong University, Shandong, China; 2 Medicine Research Center, Shandong Second Medical University, Shandong, China; 3 College of Nursing, Shandong Second Medical University, Shandong, China; 4 Department of Pathology, First Affiliated Hospital of Shandong Second Medical University (Weifang People’s Hospital), Shandong, China

**Keywords:** Breast cancer, Long non-coding RNAs

## Abstract

Triple-negative breast cancer (TNBC) is a pathological subtype of breast cancer (BC) with high malignancy, strong invasiveness and poor prognosis. Long non-coding RNA (LncRNA) plays an important role during tumorigenesis. We identified that Linc00707 was upregulated in TNBC tissues by TCGA database and RT-qPCR assay, compared with normal breast tissues and other subtypes of BC. Linc00707 promoted TNBC cells proliferation, migration and invasion. Furthermore, we found that knockdown of Linc00707 influenced autophagy via PI3K/AKT/mTOR signaling pathway in TNBC cells. Linc00707 affected the progress of TNBC cells through affecting autophagy. Further mechanistic experiments confirmed that Linc00707 could competitively bind with miR-423-5p to up-regulate MARCH2 expression, ultimately promoting TNBC progression and autophagy through PI3K/AKT/mTOR pathway. In conclusion, we demonstrate that Linc00707 is a key molecule in tumor progression and may be an effective target for patients with TNBC.

## Introduction

Breast cancer (BC) is currently the most common malignancy in women worldwide [1–3]. Expression profiling of invasive breast carcinomas by DNA microarray techniques has identified five distinct subtypes of tumors (Luminal A, Luminal B, normal breast-like, HER2 overexpression, and basal-like) that are associated with different clinical outcomes and with different chemotherapy. Basal-like carcinoma is associated with younger patient age, high histological grade, aggressive clinical course, development of distant metastasis, poor prognosis, and relatively high mortality rate. Basal-like carcinomas do not express estrogen receptor, progesterone receptor, or HER2 (triple-negative phenotype) [4, 5]. Triple-negative breast cancer (TNBC) remains the most challenging BC subtype to treat. To date, therapies directed to specific molecular targets have rarely achieved clinically meaningful improvements in outcomes of patients with TNBC, and chemotherapy remains the standard of care. High-risk early TNBC is frequently associated with early recurrence and high mortality [6, 7].

Autophagy is a conserved ubiquitous process and energy recycling system that delivers damaged organelles, misfolded proteins and intracellular constituents to lysosomes for degradation [8]. And autophagy has opposing, context-dependent roles in cancer, and interventions to both stimulate and inhibit autophagy have been proposed as cancer therapies [9, 10]. This led to the therapeutic targeting of autophagy in cancer to be sometimes viewed as controversial. Previous studies have reported that autophagy promotes survival, proliferation, metastasis, and invasion of BC cells via regulating autophagy associated genes and non-coding RNAs [11, 12]. However, the underlying mechanisms of autophagy in TNBC progression are not yet fully elucidated.

Long non-coding RNAs (LncRNAs) comprise a heterogeneous family of RNA molecules longer than 200 nucleotides with no or limited protein-coding potential [13]. LncRNAs participate in modulating biological processes through regulating gene expression at almost all levels, including chromatin remodeling, transcription, and post-transcription [14]. Linc00707 is a 3087 bp ncRNA, located on chromosome 10p14, could promote lung adenocarcinoma, hepatocellular carcinoma, cervical cancer and glioma progression by regulating with target genes [15–17]. High expression of Linc00707 mediates a series of biological functions, including cell proliferation, apoptosis, metastasis, invasion and cell cycle [18]. High Linc00707 expression in these tumors is indicative of poor prognosis [19–21]. While, the underlying mechanisms of Linc00707 in regulating autophagy and progression of TNBC are not yet fully elucidated.

In this study, we attempted to investigate the role of Linc00707 in regulating the tumor progression and autophagy and explored the underlying mechanisms in TNBC. We demonstrated that Linc00707 specifically promoted TNBC cell progression via increasing PI3K/AKT/mTOR pathway signaling and inhibiting autophagic activity by the miR-423-5p/membrane associated ring-CH-type finger 2 (MARCH2) axis.

## Results

### Linc00707 is upregulated in TNBC patients and cell lines

**Fig. 1:**
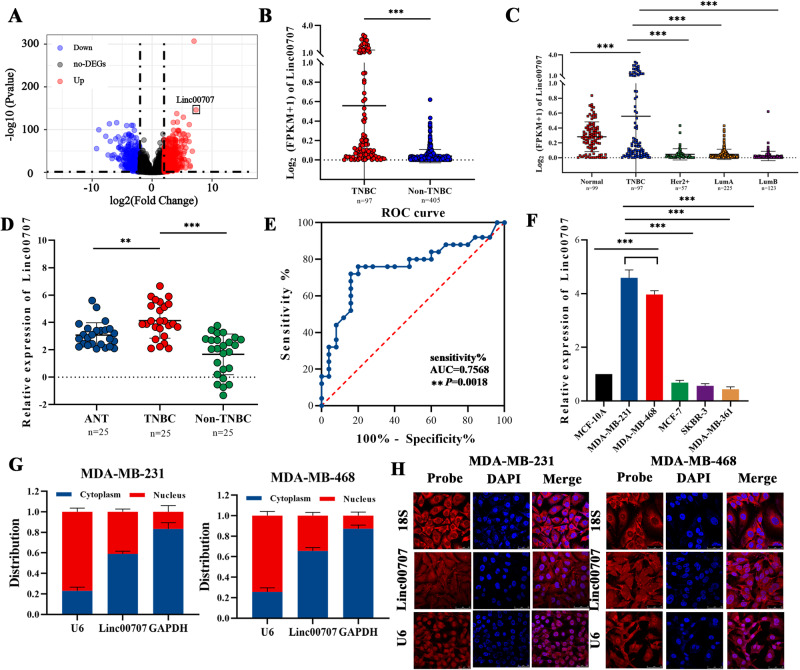
Linc00707 is upregulated in TNBC patients and cell lines. **A** Volcano plot showed that 13,582 LncRNAs were differential expression by using lncRNA expression thresholds of more than twofold change with *P* < 0.05 in TCGA. (no -DEGs: no difference, UP: upregulation, Down: downregulation). **B** The Linc00707 expression in TNBC and Non-TNBC was obtained from TCGA database. **C** The Linc00707 expression in TNBC, normal breast tissues (Normal), Her2 overexpressing breast cancer tissues (Her2+), Luminal A (LumA) and Luminal B (LumB) was obtained from TCGA database. **D** qRT-PCR analyses of Linc00707 expression levels in TNBC specimens compared with ANT and Non-TNBC. **E** ROC curve for diagnosis of TNBC by Linc00707 level in tissues. **F** The expression of Linc00707 in MCF-10A and different breast cancer cell lines. **G** The expression level of Linc00707 in the subcellular fractions of MDA-MB-231 and MDA-MB-468 cells used by RT-qPCR. U6 and GAPDH were used as nuclear and cytoplasmic markers, respectively. **H** The expression of Linc00707 in MDA-MB-231 and MDA-MB-468 cells by RNA FISH. Nuclei were stained with DAPI (blue) and Linc00707 probes were labeled with Cy3 (red). U6 RNA were used as positive controls of in the nucleus and 18 S were used as positive controls of in the cytoplasm. Scale bar, 50 μm. All experiments were repeated independently three times. Data are presented as means ± standard deviation. *P < 0.05, **P < 0.01, ***P < 0.001.

To identify aberrantly expression of LncRNAs in BC, the RNA sequencing (RNA-seq) datasets of BC was downloaded from TCGA for bioinformatics analysis. The results showed that 377 LncRNAs were significantly downregulated and 609 LncRNAs were significantly upregulated in the TNBC compared with Non-TNBC. And among all up-regulated genes, the Log2 Fold Change of Linc00707 was the largest and the *P*-value was the second smallest, therefore, we selected Linc00707 as our study subject (Fig. 1A). Meanwhile, the expression of Linc00707 in TNBC was significantly higher than that in Non-TNBC (Fig. 1B). TCGA database showed that the expression of Linc00707 in TNBC was significantly higher than in normal breast tissues (Normal), Her2 overexpressing breast cancer tissues (Her2+), Luminal A (Lum A) and Luminal B (Lum B) (Fig. 1C). To confirm the accuracy of the database, the expression of Linc00707 in 25 Non-TNBC, 25 TNBC samples and adjacent normal tissues (ANT) was detected by RT-qPCR. The results showed that Linc00707 was also expressed at higher levels in TNBC specimens compared with ANT and Non-TNBC (Fig. 1D). In order to further explore the diagnostic values of Linc00707 in TNBC, we performed receiver operating characteristic (ROC) curve analysis to assess the potential use of Linc00707 as a non-invasive molecular biomarker, and effectively distinguish TNBC tissue from normal breast tissue. The results showed that the cut-off value was 3.54, the area under curve (AUC) was 0.7568 (95% CI = 0.62–0.90) and with a sensitivity of 76% and specificity of 80% (*P* = 0.0018) (Fig. 1E). Next, we assessed the expression of Linc00707 in human normal mammary epithelial cells (MCF-10A) and Luminal A (MCF-7), Luminal B (MDA-MB-361), TNBC (MDA-MB-231 and MDA-MB-468) and HER2+ (SKBR-3) of the breast cancer cells. Linc00707 was highly expressed in MDA-MB-231 and MDA-MB-468 cells compared with MCF-7, MDA-MB-361 and SKBR-3 cells and MCF-10A (Fig. 1F). The results of nuclear/cytoplasmic RNA fractionation from the subcellular distribution assay confirmed that Linc00707 was mainly located in the cytoplasm of MDA-MB-231 and MDA-MB-468 cells (Fig. 1G). Confocal microscopy for RNA FISH also further confirmed that Linc00707 was distributed in both nuclei and cytoplasm, but mainly distributes in the cytoplasm (Fig. 1H).

### Linc00707 promotes TNBC cells proliferation, migration and invasion

**Fig. 2:**
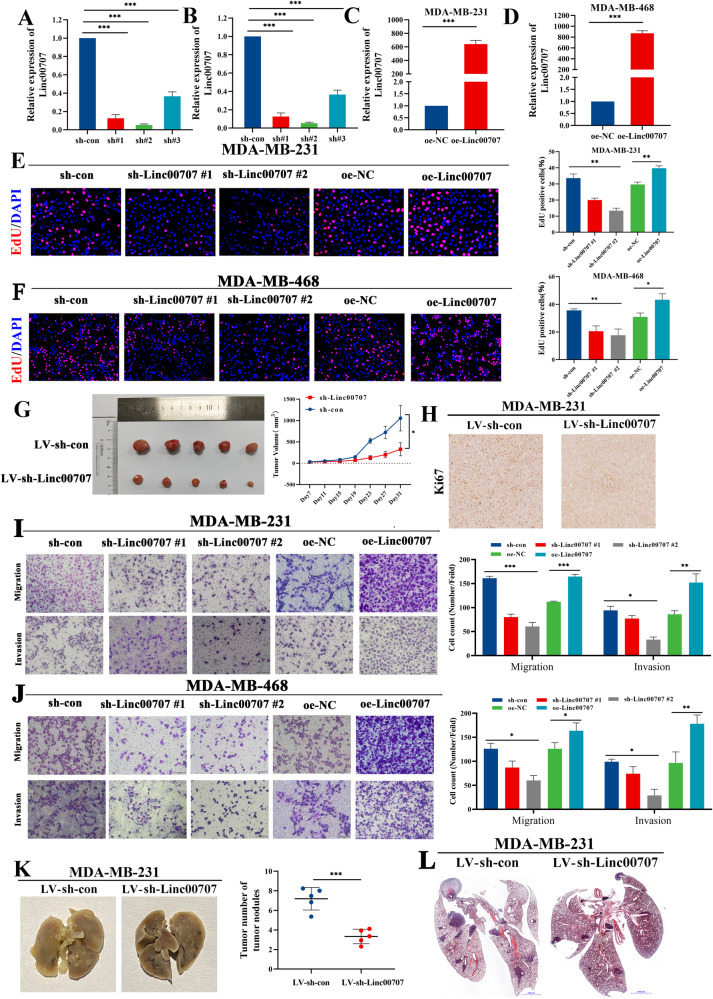
Linc00707 promotes TNBC cells proliferation, migration and invasion. **A**, **B** Analysis of Linc00707 expression after siRNAs transfection in MDA-MB-231 and MDA-MB-468 cells by RT-qPCR. **C**, **D** Analysis of Linc00707 expression after overexpression vector transfection in MDA-MB-231 and MDA-MB-468 cells by RT-qPCR. **E** EdU assay of the cell proliferation ability in MDA-MB-231 cells. Scale bar: 100 μm. **F** EdU assay of the cell proliferation ability in MDA-MB-468 cells. Scale bar: 100 μm. **G** The nude mice were subcutaneously injected with 5 × 10^6^ MDA-MB-231 cells stably transfected with LV-sh-Linc00707 or LV-sh-con cells. A ruler was used to indicate the size of the tumors. The tumor weight with different cells was shown. **H** Immunohistochemistry for Ki-67 detection in LV-sh-con and LV-sh-Linc00707 group. **I**, **J** Transwell assay in MDA-MB-231 and MDA-MB-468 cells transfected with sh-con or sh-Linc00707 #1 or sh-Linc00707 #2, oe-NC or oe-Linc00707. Scale bar: 100 μm. **K** Representative images of the lung obtained from nude mice and numbers of lung metastasis lesions were calculated. **L** HE staining of lung tissues were used to detect the metastasis nodules. All experiments were repeated independently three times. Data are presented as means ± standard deviation. *P < 0.05, **P < 0.01, ***P < 0.001.

In order to investigate the role of Linc00707 in TNBC progression, we downregulated Linc00707 with shRNA or overexpressed Linc00707 using pcDNA3.0 plasmid vector in MDA-MB-231 and MDA-MB-468 cells. The knockdown and overexpression efficiency were confirmed by RT-qPCR (Fig. 2A–D). We chose the first and second targets with low knockdown efficiency for subsequent functional tests. To explore the effect of Linc00707 on TNBC cell proliferation, EdU and colony formation assays were performed. The results showed that inhibition of Linc00707 expression reduced EdU staining (Fig. 2E, F). Consistently, overexpression of Linc00707 significantly increased EdU staining (Fig. 2E, F). Coinciding results of the EdU assay, colony formation assay confirmed that silencing of Linc00707 markedly impaired the proliferation abilities of TNBC cells, and overexpression of Linc00707 promoted the proliferation abilities significantly (Supplementary Fig. 1A, B).

To analyze the effects of Linc00707 on TNBC cells in vivo, xenograft nude mouse model experiments were performed. The tumor volumes in the LV-sh-Linc00707 group were significantly smaller compared to the LV-sh-con group (Fig. 2G); Conversely, the tumor volumes in the LV-oe-Linc00707 group were larger than compared to the LV-oe-NC group (Supplementary Fig. 2A). Immunohistochemistry staining for Ki-67 showed that tumor cells in LV-sh-Linc00707 group had less proliferative activity compared with those in LV-sh-con group (Fig. 2H). Conversely, tumor cells in the LV-oe-LincC00707 group had more proliferative activity than those in the LV-oe-NC group (Supplementary Fig. 2B). In summary, these data demonstrated that downregulation of Linc00707 suppressed tumor growth and upregulation of Linc00707 promoted tumor growth in vivo.

Then, we used Transwell assays to detect the migration and invasion ability of the TNBC cells. The results demonstrated that silencing of Linc00707 profoundly suppressed the ability of migration and invasion in TNBC cells, whereas increasing of Linc00707 increased the ability of such cells (Fig. 2I, J). Wound healing assays showed that downregulation of Linc00707 significantly decreased cell migratory behaviors of TNBC cells and upregulation of Linc00707 significantly increased cell migratory behaviors of TNBC cells (Supplementary Fig. 1C, D). Additionally, mice injected with LV-sh-Linc00707 cells had much fewer metastatic nodules in the lungs than mice injected with LV-sh-con cells (Fig. 2K). On the contrary, mice injected with LV-oe-Linc00707 cells had more metastatic nodules in the lungs than mice injected with LV-oe-NC cells (Supplementary Fig. 2C). Consistent with the in vitro results, downregulated expression of Linc00707 suppressed the ability of MDA-MB-231 cells to spread to the lung after tail vein injection, Linc00707 overexpression caused the opposite result. HE staining slices further confirmed that knockdown of Linc00707 significantly diminished the number of lung metastasis nodules (Fig. 2L), and overexpression of Linc00707 significantly increased the number of lung metastatic nodules (Supplementary Fig. 2D). Collectively, these results suggest that Linc00707 plays a critical role in the proliferation and migration of TNBC cells.

### Linc00707 influences autophagy in TNBC cells via PI3K/AKT/mTOR signaling pathway

**Fig. 3:**
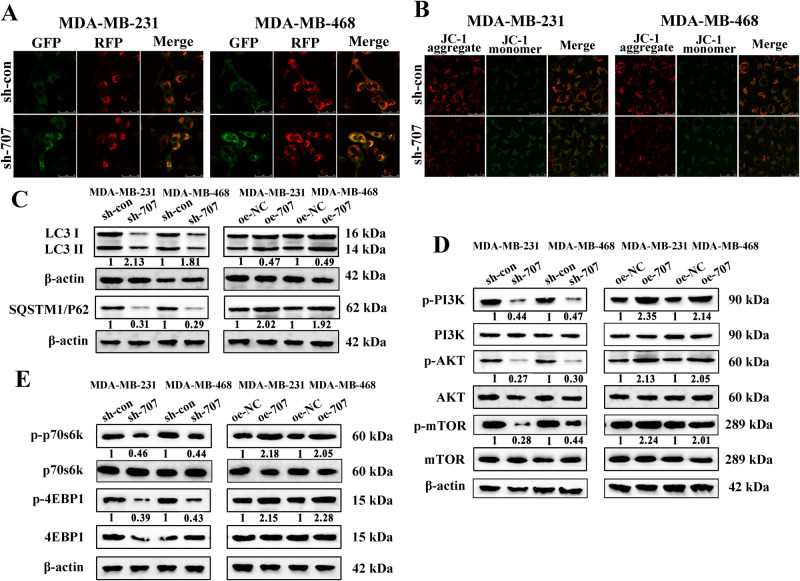
Linc00707 influences autophagy in TNBC cells via PI3K/AKT/mTOR signaling pathway. **A** The MDA-MB-231 and MDA-MB-468 cell lines stably expressing stubRFP-sensGFP-LC3 were transfected with sh-con and sh-Linc00707 plasmids were observed by the fluorescence microscope, and the StubRFP-SensGFP-LC3 fluorescence spots were photographed under laser confocal microscopy. GFP represented autophagosomes, RFP represented the total number of autophagosomes and autolysosomes, yellow puncta in the merged image represented the flow rate of autophagic flow from autophagosomes to autolysosomes. Scale bar: 50 μm. **B** The changes of mitochondrial membrane potential of sh-con and sh-linc00707 groups in MDA-MB-231 and MDA-MB-468 were monitored by JC-1. Green fluorescence: JC-1 monomer, red fluorescence: JC-1 aggregates. Scale bar: 50 μm. **C** Western Blot analysis of the ratio of LC3-II/LC3-I and protein levels of SQSTM1/P62 in MDA-MB-231 and MDA-MB-468 cells transfected with sh-con or sh-Linc00707, oe-NC or oe-Linc00707. Western blot results are expressed as fold changes in relative band densities to control from three independent experiments. **D** Western Blot analysis of protein levels of p-PI3K, PI3K, p-AKT, AKT, p-mTOR, mTOR in MDA-MB-231 and MDA-MB-468 cells transfected with sh-con or sh-Linc00707, oe-NC or oe-Linc00707. Western blot results are expressed as fold changes in relative band densities to control from three independent experiments. **E** Western Blot analysis of protein levels of p-p70s6k, p70s6k, p-4EBP1, 4EBP1 in MDA-MB-231 and MDA-MB-468 cells transfected with sh-con or sh-Linc00707, oe-NC or oe-Linc00707. Western blot results are expressed as fold changes in relative band densities to control from three independent experiments. All experiments were repeated independently three times. Data are presented as means ± standard deviation. * P < 0.05.

Autophagy is integral to human health and is involved in physiology, development, and a wide range of diseases, including cancer [22–24]. To further explore the effects of Linc00707 on the autophagic response, we obtained 13497 differential protein-coding genes through TCGA database analysis. A total of 222 autophagy-related proteins were obtained by HADb database analysis. 168 differentially expressed proteins related to autophagy were obtained (Supplementary Fig. 3A). Spearman correlation analysis was carried out between the 168 differentially expressed proteins and the expression levels of Linc00707. Ten autophagy-related proteins, TP63, NRG1, FAS, ITGB, CFLAR, PRKCQ, TMEM74, CX3CL1, EGFR, NRG2, were obtained by using R > 0.4, *P* < 0.01 as screening conditions (Supplementary Fig. 3B). Therefore, we speculate that Linc00707 is involved in the process of autophagy, and so far, no study has found Linc00707 to be related to autophagy, so we chose autophagy as the research object. Then we introduced a stubRFP-sensGFP-LC3 reporter system to monitor changes in autophagic flux. The number of red fluorescent dots in cells represents the total number of autophagosomes and autolysosomes, and the number of green fluorescent dots represents the number of autophagosomes, and yellow puncta in the merged image represents the flow rate of autophagic flow from autophagosomes to autolysosomes. The results showed that autophagosomes and the flow rates of autophagic flow from autophagosomes to autolysosomes were increased in the merged image in the sh-Linc00707 group compared with the control group, indicating that knockout of Linc00707 promoted autophagy of MDA-MB-231 and MDA-MB-468 cells (Fig. 3A). Thus, changes in intracellular Mitochondrial membrane potential (MMP) were examined after knockdown of Linc00707 in MDA-MB-231 and MDA-MB-468 cells by JC-1. The results showed that compared with the control group, the red fluorescence decreased and the green fluorescence increased in sh-Linc00707 group and the results indicated that knockdown of Linc00707 could reduce MMP (Fig. 3B). LC3 is the key molecule for the fusion of autophagosome and lysosome. During autophagy, LC3 is cleaved by the protease ATG4 to produce LC3I, which binds to phosphatidylethanolamine to form LC3II and anchors to the autophagosome membrane to form a fully closed autophagosome, therefore, LC3II/LC3I ratio can estimate the functional status of autophagy [22–24]. P62 proteins, also known as SQSTM1, are sent to autophagosomes for degradation by membrane-bound LC3II proteins, and thus the accumulation of SQSTM1/P62 proteins in the cytoplasm is used as a marker of reduced autophagic flux [25]. The results showed that the ratio of LC3II/LC3I in MDA-MB-231 and MDA-MB-468 cells of the sh-Linc0070 group was significantly higher than the control group, and the SQSTM1/P62 protein expression was lower than the control group. These results indicated that the autophagy was activated. Conversely, the ratio of LC3II/LC3I in MDA-MB-231 and MDA-MB-468 cells of the oe-Linc00707 group was significantly lower than the control group, and the SQSTM1/P62 protein expression was higher than the control group. These results showed that the autophagy was inhibited (Fig. 3C). The PI3K/AKT/mTOR pathway, as a critical regulator of autophagy, is involved in the initiation and promotion of a series of pathological disorders including various tumors [26]. To determine the mechanisms by which Linc00707 regulates autophagy, we investigated the effect of Linc00707 on the mTOR pathway. And we found that phosphorylation of PI3K, AKT, mTOR were increased significantly after Linc00707 overexpression, while PI3K, AKT, mTOR phosphorylation was inhibited in MDA-MB-231 and MDA-MB-468 cells after Linc00707 inhibition, as compared with control groups (Fig. 3D). MTOR pathway inhibition has been reported to play an important role in autophagy initiation, and mTOR activity can be inferred by the levels of phosphorylation of its substrates (p70S6K and 4EBP1) [27–29]. So, we detected the phosphorylation of p70S6K and 4EBP1, the results showed that the levels of phosphorylation of p70S6K and 4EBP1 were inhibited in TNBC cells of Linc00707 knockdown. But the levels of phosphorylation of p70S6K and 4EBP1 were increased significantly after Linc00707 overexpression (Fig. 3E). To further verify whether Linc00707 affected autophagy through the PI3K/AKT/mTOR signaling pathway, we observed the changes in autophagy after treatment with the PI3K inhibitor LY294002. The results showed that autophagosomes and the flow rates of autophagic flow from autophagosomes to autolysosomes were increased in the merged image in the sh-Linc00707 + LY294002 group compared with controls group added with the LY294002, but autophagy did not change with or without LY294002 (Supplementary Fig. 4A). JC-1 results showed that compared with the control group, the red fluorescence decreased and the green fluorescence increased in sh-Linc00707 + LY294002 group, but MMP did not change with or without LY294002 (Supplementary Fig. 4B). These results suggest that Linc00707 regulates autophagy in TNBC cells via activating PI3K/AKT/mTOR signaling pathway.

### Linc00707 affects the progress of TNBC cells through affecting autophagy

**Fig. 4:**
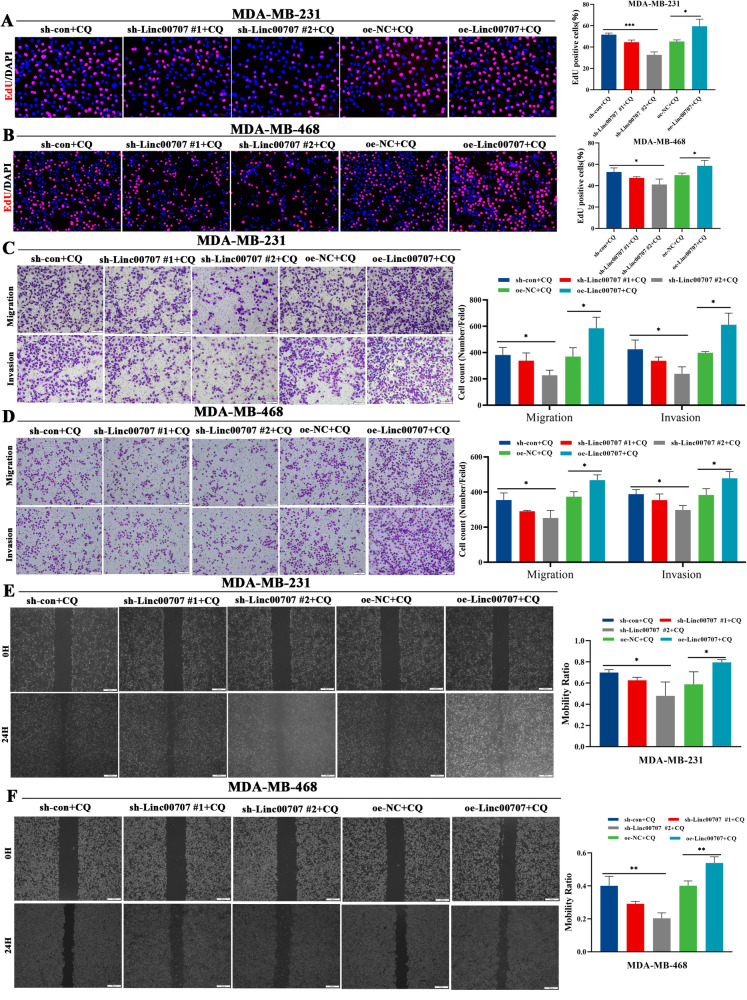
Linc00707 affects the progress of TNBC cells through affecting autophagy. **A**, **B** EdU analysis of the cell proliferation capacity in MDA-MB-231 and MDA-MB-468 cells transfected with sh-con, sh-Linc00707 #1, sh-Linc00707 #2, oe-NC or oe-Linc00707 after CQ treatment. Scale bar: 100 μm. **C**, **D** Transwell assay analysis of the cell invasion and migration capacity in MDA-MB-231 and MDA-MB-468 cells transfected with sh-con, sh-Linc00707 #1, sh-Linc00707 #2, oe-NC or oe-Linc00707 after CQ treatment. Scale bar: 100 μm. **E**, **F** Wound healing assay analysis of the cell migration capacity in MDA-MB-231 and MDA-MB-468 cells transfected with sh-con, sh-Linc00707 #1, sh-Linc00707 #2, oe-NC or oe-Linc00707 after CQ treatment. Scale bar: 500 μm. All experiments were repeated independently three times. Data are presented as means ± standard deviation. *P < 0.05, **P < 0.01, ***P < 0.001.

To further verify whether Linc00707 affected the invasion, migration and proliferation of TNBC cells through autophagy, we added the autophagy inhibitor CQ to MDA-MB-231 and MDA-MB-468 cells that were knockdown and overexpression of Linc00707. When CQ was added, EdU assay results showed that the proliferation activity of TNBC cells was decreased in the Linc00707 knockdown group compared with the control group, whereas it was enhanced in the Linc00707 overexpression group (Fig. 4A, B). Meanwhile, the proliferation activity of TNBC cells was significantly enhanced in all CQ treatment groups compared with non CQ treatment groups (Figs. 2E, F and 4A, B). Transwell assay results showed that the number of transmembrane cells was reduced in the Linc00707 knockdown group compared with the control group after CQ treatment. Conversely, the number of transmembrane cells was increased in the Linc00707 overexpression group after CQ treatment (Fig. 4C, D). Meanwhile, the number of TNBC transmembrane cells increased in all CQ treatment groups compared with non CQ treatment groups (Figs. 2I, J and 4C, D). The Wound healing assay results showed that the scratch spacing was widened in Linc00707 knockdown cells compared with the control groups after CQ treatment. In contrast, the scratch spacing was narrower in the Linc00707 overexpressing group after CQ treatment (Fig. 4E, F). Moreover, the scratch spacing of TNBC cells was significantly narrower in all CQ treatment groups compared with non CQ treatment groups (Supplementary figs. 1C, D and Fig. 4E, F). These results indicate that Linc00707 can affect TNBC cells progression through affecting autophagy.

### Linc00707 sponges miR-423-5p and is negatively associated with miR-423-5p expression

**Fig. 5:**
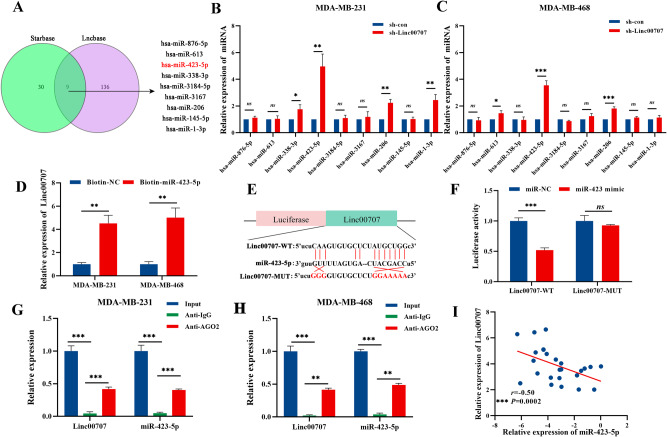
Linc00707 sponges miR-423-5p and is negatively associated with miR-423-5p expression. **A** The Venn diagram of miRNAs binding to Linc00707 were predicted using by using online database Starbase v.2.0 and LncBase v.2.0. **B**, **C** qRT-PCR analyses of the relative levels of 9 miRNA candidates in sh-Linc00707 and sh-con of MDA-MB-231 and MDA-MB-468. **D** Relative Linc00707 level in MDA-MB-231 and MDA-MB-468 cells lysates captured by biotin-labeled miR-423-5p or miR-NC was detected by RT-qPCR. **E** Binding sites of Linc00707 and miR-423-5p. **F** Relative luciferase activities in HEK293T cells co-transfected with Linc00707-WT or Linc00707-MUT and miR-423 overexpression vector (miR-423 mimics) or miR-NC. **G**, **H** Anti-AGO2 RIP was performed using MDA-MB-231 and MDA-MB-468 cells followed by RT-qPCR to detect Linc00707 and miR-423-5p. **I** Pearson correlation analysis of Linc00707 expression and miR-423-5p level in 25 TNBC samples. All experiments were repeated independently three times. Data are presented as means ± standard deviation. ns *P* ≥ 0.05, * *P* < 0.05, ** P < 0.01, *** P < 0.001.

The lncRNA located in the cytoplasm can be used as a competitor of mRNA binding miRNA to affect the expression of downstream target genes through the competing endogenous RNA (ceRNA) mechanism. At the same time an anti-AGO2 RIP assay showed that Linc00707 were pulled down by the anti-AGO2 antibody but not by IgG (Fig. 5G, H). Therefore, we speculate that Linc00707 participates in the regulation of mRNA through ceRNA mechanism. The putative candidate miRNAs binding to Linc00707 were predicted using LncBase Predicted v.2.0 and Starbase v.2.0. The results showed that there are 9 miRNAs (miR-876-5p, miR-613, miR-338-3p, miR-423-5p, miR-3184-5p, miR-3167, miR-206, miR-145-5p, miR-1-3p) shared by two prediction websites (Fig. 5A). Then, we used RT-qPCR to detect the expression of 9 miRNAs in TNBC cells after Linc00707 knockdown, and the results showed that miR-423-5p was most significantly up-regulated in sh-Linc00707 cells compared with sh-con (Fig. 5B, C). Accordingly, biotin labelled miR-423-5p could directly pull down Linc00707, ruling out the possibility of false positives due to indirect pull-down assays (Fig. 5D). Then, in order to verify whether miR-423-5p can bind to Linc00707, a pmirGLO-Linc00707 dual luciferase plasmid was constructed for dual luciferase experiments. The luciferase activity was detected after 48 h of co-transfection miR-423 mimics and pmirGLO-Linc00707 (Linc00707-WT) into HEK293T cell lines. The results showed that the luciferase activity was highly decreased in cells of transfection miR-423 mimics and Linc00707-WT. But when we tried the same experiment with a mutated Linc00707 (Linc00707-MUT), it was clear that there was no significant change in the luciferase activity in the presence of miR-423-5p (Fig. 5E, F). Accordingly, an anti-AGO2 RIP assay was performed, and the results showed that miR-423-5p were pulled down by the anti-AGO2 antibody but not by IgG (Fig. 5G, H). Additionally, pearson correlation analysis of Linc00707 expression and miR-423-5p level in 25 TNBC samples, the results showed Linc00707 expression was inversely associated with miR-423-5p levels (Fig. 5I). Taken together, the above results implied that Linc00707 served as a ceRNA via sponging miR-423-5p.

### Inhibition of miR-423-5p reverses the suppressive effects of Linc00707 downregulation on TNBC cells progress

**Fig. 6:**
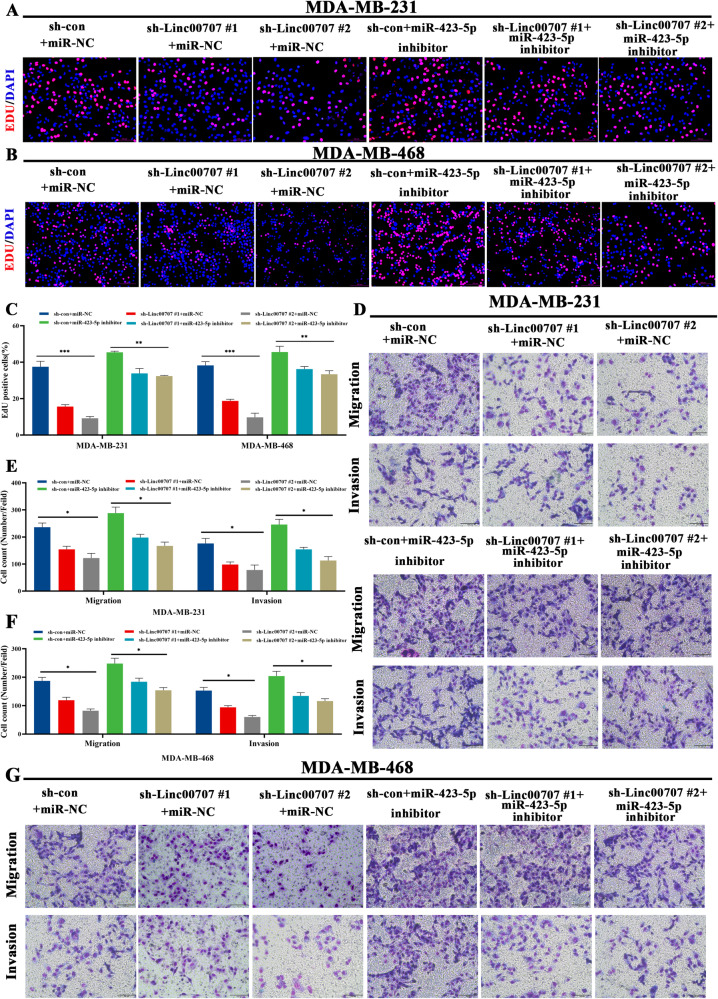
Inhibition of miR-423-5p reverses the suppressive effects of Linc00707 downregulation on TNBC cells progress. **A**, **B** Typical images of of the cell proliferation ability in MDA-MB-231 and MDA-MB-468 cells transfected with sh-con+miR-NC, sh-Linc00707 #1+miR-NC, sh-Linc00707 #2+miR-NC, sh-con+miR-423-5p inhibitor, sh-Linc00707 #1+miR-423-5p inhibitor, sh-Linc00707 #2+miR-423-5p inhibitor by EdU assay. Scale bar: 100 μm. **C** EdU statistical analysis of the cell proliferation ability in MDA-MB-231 and MDA-MB-468 cells. **D** Typical images of Transwell assay in MDA-MB-231 cells transfected with sh-con+miR-NC, sh-Linc00707 #1+miR-NC, sh-Linc00707 #2+miR-NC, sh-con+miR-423-5p inhibitor, sh-Linc00707 #1+miR-423-5p inhibitor, sh-Linc00707 #2+miR-423-5p inhibitor. Scale bar: 100 μm. **E**, **F** Transwell statistical analysis of the cell migration and invasion ability in MDA-MB-231 and MDA-MB-468 cells. **G** Typical images of Transwell assay in MDA-MB-468 cells transfected with sh-con+miR-NC, sh-Linc00707 #1+miR-NC, sh-Linc00707 #2+miR-NC, sh-con+miR-423-5p inhibitor, sh-Linc00707 #1+miR-423-5p inhibitor, sh-Linc00707 #2+miR-423-5p inhibitor. Scale bar: 100μm. All experiments were repeated independently three times. Data are presented as means ± standard deviation. *P < 0.05, **P < 0.01, ***P < 0.001.

To determine whether Linc00707 functions as an oncogene via miR-423-5p, we performed rescue experiments. We cotransfected sh-Linc00707 and miR-423-5p inhibitors into TNBC cells to determine whether the oncogene effect of Linc00707 could be blocked by miR-423-5p inhibitors. The results of EdU assays showed that miR-423-5p inhibitors could partly increase the proliferation rate and reversed the suppressive effects on these processes induced by Linc00707 downregulation in MDA-MB-231 and MDA-MB-468 cells (Fig. 6A–C). Similar results were observed in colony formation (Supplementary Fig. 5A–C). Further, Transwell showed that knockdown of miR-423-5p further reversed the inhibitory effect of knockdown of Linc00707 on the malignant properties of TNBC cells (Fig. 6D–G). And wound healing results showed that knockdown of miR-423-5p further reversed the inhibitory effect of knockdown of Linc00707 on the migration ability of TNBC cells (Supplementary Fig. 5D–F). Therefore, these data revealed that Linc00707 promoted TNBC cell proliferation and metastasis by sponging miR-423-5p.

### Linc00707/miR-423-5p/MARCH2 regulates autophagy via the PI3K/AKT/mTOR pathway

**Fig. 7:**
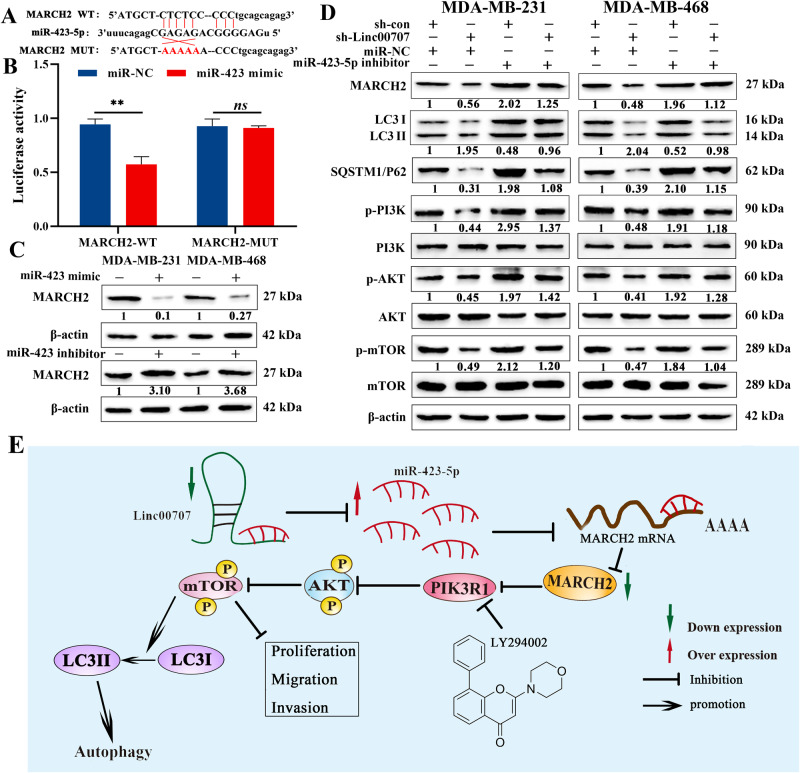
Linc00707/miR-423-5p/MARCH2 regulates autophagy via the PI3K/AKT/mTOR pathway. **A** Binding sites of miR-423-5p and MARCH2. **B** The relative luciferase activities in HEK293T cells co-transfected with MARCH2-WT or MARCH2-MUT and miR-423 overexpression vector (miR-423 mimic) or miR-NC. **C** Western blot analysis of the protein expression level of MARCH2 in MDA-MB-231 and MDA-MB-468 cells. Western blot results are expressed as fold changes in relative band densities to control from three independent experiments. **D** Western blot analysis of the protein expression level of MARCH2, LC3-II/LC3-I, SQSTM1/P62,p-PI3K, PI3K, p-AKT, AKT, p-mTOR, mTOR in MDA-MB-231 and MDA-MB-468 cells with different cotransfection. Western blot results are expressed as fold changes in relative band densities to control from three independent experiments. **E** Schematic representation of the mechanism showed that Linc00707/miR-423-5p/MARCH2 regulates autophagy and progression of TNBC cells via the PI3K/AKT/mTOR pathway. All experiments were repeated independently three times. Data are presented as means ± standard deviation. ns *P* ≥ 0.05, **P < 0.01.

The previous study had found that depletion of MARCH2 greatly elevated the levels of endogenous LC3II and regulated autophagy by PIK3CA/AKT/mTOR signaling [30]. Online prediction software showed that there was a binding site between miR-432-5p and MARCH2 (Fig. 7A). Thus, we wondered whether MARCH2 contributed to the effect of Linc00707 and miR-423-5p in TNBC cells. To elucidate the interaction between miR-423-5p and MARCH2, we inserted the luciferase reporter with wild type (MARCH2-WT) or mutant-type (MARCH2-MUT) sequences that were predicted to be potential binding sites for miR-423-5p and MARCH2. The relative luciferase activity was decreased by miR-423-5p mimic in HEK293T cells transfected with MARCH2-WT constructs while no significant difference was observed in the MARCH2-MUT group (Fig. 7B). Subsequently, miR-423-5p significantly reduced MARCH2 protein levels (Fig. 7C), suggesting that MARCH2 is a direct target gene of the miR-423-5p. Likewise, Western Blot showed knockdown of Linc00707 inhibited the expression of MARCH2 and SQSTM1/P62 protein and increased the LC3-II/LC3-I ratio, while, knockdown of miR-423-5p promoted the expression of MARCH2 and SQSTM1/P62 protein and also lowed the LC3-II/LC3-I ratio. And the amplification effects of Linc00707 could be partly reversed by miR-423-5p downregulation (Fig. 7D). Then, further experiments were performed to determine the influence of Linc00707 and miR-423-5p on the PI3K/AKT/mTOR pathway. As shown in Fig. 7D, there was a significant decrease in the phosphorylation of PI3K, AKT, mTOR after knockdown of Linc00707, indicating Linc00707 leads to activation of the PI3K/AKT/mTOR axis. At the same time, miR-423-5p silencing increased the expression of the phosphorylation of PI3K, AKT and mTOR. Collectively, these results suggested that Linc00707/miR-423-5p/MARCH2 regulates autophagy and progression of TNBC cells via the PI3K/AKT/mTOR pathway (Fig. 7E).

## Discussion

The discovery of the functional significance of protein-coding genes has led to great successes in the progression of targeted therapies against cancer [31]. Numerous evidences have suggested that nonprotein-coding genes account for the overwhelming majority of RNA transcripts and can drive important cancer phenotypes. Such discoveries have sparked great interest in the functions and regulation of LncRNAs [32].

Both LncRNAs and miRNAs have dynamic roles in transcriptional and translational regulation, and are involved in many human diseases, especially in cancer [33]. For example, long noncoding RNA LINC00336 inhibits ferroptosis in lung cancer by functioning as a ceRNA [34], and miR-423-5p inhibits osteosarcoma proliferation and invasion through directly targeting STMN1 [35]. These discoveries encouraged us to explore the potential roles of miRNAs in binding and regulating LncRNAs. Our functional characterization revealed that silencing Linc00707 reduced TNBC tumor growth and metastasis. Interestingly, we detected significant compartment differences between the sh-con and sh-Linc00707 by StuRFP-sensGFP-LC3 autophagic flux, which are supported by our immunofluorescence analyses. MMP is beneficial to maintain the normal physiological function of cells, JC-1 is an ideal fluorescence probe widely used for the detection of MMP [36]. At a high MMP, JC-1 aggregates in the matrix of mitochondria, forming J-aggregates that can produce red fluorescence; at a low MMP, JC-1 can’t aggregate in the matrix of mitochondria. At this point JC-1 is a monomer and can produce green fluorescence. The decrease of MMP is the early sign of cell death [37]. The results showed that knockdown of Linc00707 could reduce MMP. Then, this study further characterized that overexpression of miR-423-5p eliminated the promotive effect of Linc00707 on malignant behaviors. And Linc00707 promotes TNBC proliferation and metastasis by sponging miR-423-5p to modulate MARCH2 expression and regulate autophagy.

MARCH2 is a member of the MARCH family, which includes at least 9 members currently. MARCH2 is characterized by a RING-CH finger (N terminus), 2 transmembrane spans (middle) and a PDZ domain binding motif (C terminus) [38]. MARCH2 was identified for the first time as a member of a novel transmembrane ubiquitin ligase family probably associated with viral immune evasion proteins. To verify whether the autophagy effect in MARCH2 knockdown cells is a result of aberrant PI3K/AKT/mTOR signaling. Dan Xia et al. [30] reported that siMARCH2 can induce autophagy by reducing the phosphorylation level of PI3K/AKT/mTOR. In contrast, overexpression of MARCH2 increased the phosphorylation level of PI3K/AKT/mTOR and inhibited the development of autophagy. This result suggests that the regulation of PI3K/AKT/mTOR by MARCH2 is related to autophagy. Furthermore, LC3 is a target gene downstream of PIK3R1, PIK3R1is a regulatory subunit of class I PI3K, encodes p85a protein. When PIK3R1/AKT signaling inactivates mTOR, which consequently induces autophagy. Autophagy is activated. Next, LC3I was modified by ubiquitin-like processing and combined with the Phosphatidylethanolamine on the surface of autophagy membrane to form LC3II. Further investigation revealed that miR-423-5p could regulate the autophagy of TNBC cells by targeting and binding with MARCH2 to regulate progression of BC cells. These founding have strongly suggested that miR-423-5p may play important role in cancer development. All results shown that Linc00707 acts as a ceRNA of the miR-423-5p to regulate MARCH2 to inhibit autophagy and promote progression of TNBC cells.

In summary, it is our novel discovery of a positive feed-back loop between the Linc00707 and PI3K/AKT/mTOR signaling involved in human TNBC formation. We also illustrate that Linc00707 functions as an oncogene to facilitate tumor cell proliferation and inhibit autophagy. These findings indicate that Linc00707 is a critical molecule for tumor progression and may serve as an effective target of TNBC therapy.

## Conclusions

Our results demonstrate that Linc00707 is a key molecule in tumor progression and may be an effective target for patients with TNBC. These findings indicate that Linc00707 is a critical molecule for tumor progression and may serve as an effective target of TNBC therapy.

## Methods

### TCGA database

RNA-sequence data of BC were downloaded from TCGA (https://portal.gdc.cancer.gov/). The data included expression profile data of 99 normal tissues and 1072 tumor tissues. A total of 509 tumor tissues had both RNA-seq data and BC typing information. Therefore, 99 normal tissues and 509 tumor tissue samples were subjected to differential analysis. 509 tumor tissue samples included 97 TNBC, 57 Her2 overexpression breast cancer tissues (Her2+), 225 Luminal A (Lum A), 123 Luminal B (Lum B) and 7 other types of BC. Differential expression analysis was performed using the R package of version 4.2.2.

To further investigate the role of Linc00707 in autophagy, we downloaded BC transcriptome expression data from TCGA (total 1231 samples, Normal = 113, Tumor = 1118), 1219 samples (Normal = 113, Tumor = 1106) were obtained by deleting the transcriptome data of the duplicated and metastatic tumors (5 duplicated and 7 metastatic). The difference analysis of truncated coding protein RNA data was carried out using R language limma package.

### HADb database

The coding proteins related to autophagy were downloaded from the HADb database (http://www.autophagy.lu/index.html).

### Patient samples and ethical approval

We collected 25 pairs of TNBC tissues and corresponding adjacent noncancerous tissues (ANT), 25 Non-TNBC tissues from the affiliated hospital of Weifang Medical University in 2016-2018. Inclusion criteria: (1) TNBC and Non-TNBC (including Her2+, Lum A, Lum B) was diagnosed pathologically in the study group. (2) None of the patients enrolled in the study received chemotherapy or radiotherapy before surgery, and there was no evidence of any other malignancies. (3) All patients in this study provided written informed consent for sample collection and data analyses. Exclusion criteria: (1) Patients with major diseases of various organs. (2) Incomplete information and disagrees. (3) Other types of BC except Her2+, Lum A, Lum B. The samples were excised from patients, immediately frozen in liquid nitrogen and stored until use. All samples were collected for research use only. The research was approved by the ethics committee of Weifang Medical University.

### RNA extraction and quantitative real-time PCR (RT-qPCR)

Total RNA was extracted from tissue samples and cell using TRIzol Reagent (Sangon Biotech Shanghai) according to the manufacturer’s instructions. 1 μg RNA was reverse-transcribed by using the ReverTra Ace qPCR RT Kit (TOYOBO, Japan), then subjected to RT-qPCR reaction by use of the QuantiTect SYBR Green PCR kit (US ERBRIGHT, S2014) and ABI 7500 Fast system. All experiments were done in triplicate. Relative expression levels were calculated and normalized to endogenous U6 for miRNA and GAPDH for mRNA and Linc00707.

### Cell lines and cell culture

The human normal mammary epithelial cells (MCF-10A) and human BC cell lines MDA-MB-231, MDA-MB-468, MCF-7, SKBR-3 and MDA-MB-361 cell lines were purchased from the American Type Culture Collection (ATCC) and cultured according to their instructions. Cells were incubated at 37 °C in a humidified atmosphere with 5% CO_2_. The cell lines in the article were identified using STR analysis, and no positive results were found for detecting mycoplasma contamination.

### Subcellular fractionation

Nuclear and cytoplasmic separation was performed using the PARIS Kit (Life Technologies, USA) according to the manufacturer’s instructions.

### RNA-Fluorescence In-Situ Hybridization (RNA-FISH)

The expression and localization of Linc00707 in TNBC cells were detected by FISH method. Briefly, cells were grown on cover slides, fixed in 4% formaldehyde for 10 min at room temperature, then cleaned with PBS for 3 times, use 0.5% Triton permeable membrane, then cleaned with PBS for 3 times, Ribo fluorescence in situ hybridization kit (RiboBio, Guangzhou, China) was used for FISH detection. Cy3-labeled probes are resistant to Linc00707 or control probes are resistant to U6 snRNA and 18 S rRNA. Representative images were collected and analyzed under a confocal laser scanning microscope (TCS SP8, Leica, Germany). All experiments were independently repeated three times.

### Plasmid construction and cell transfection

For in vitro assays, three shRNAs against Linc00707 (sh-Linc00707), miR-423-5p inhibitor and their respective comparisons were purchased by Genechem (Shanghai, China). ShRNA sequences were sh#1: CAGCAGGAACATCACCATCTT, sh#2: CCTCCCAGGGATGATGGAAGGTTAA, sh#3: TACCTTCAGACACATTGATAT. The overexpressed plasmid pcDNA3.0-Linc00707 (oe-Linc00707) and the respective control plasmid pcDNA3.0 (oe-NC) were purchased by Boshang biotechnology (Jinan).

For in vivo studies, lentivirus (GV112) constructs shRNA against Linc00707 (LV-sh-Linc00707) and negative control (LV-sh-con) were purchased from Genechem (Shanghai, China) and were used for transfection of MDA-MB-231 cells to establish cells lines stably downregulating Linc00707. Additionally, lentivirus (GV513) constructs Linc00707 overexpression lentivirus (LV-oe-Linc00707) and negative control (LV-oe-NC) were purchased from Genechem (Shanghai, China) and were used for transfection of MDA-MB-231 cells to establish cells lines stably upregulating Linc00707.

### Cell proliferation assay

Cell proliferation was detected with EdU assays and clone formation assay. The EdU assay was performed according to the protocol of the BeyoClick™ EdU-594 cell proliferation detection kit (Beyotime Biotechnology, Shanghai, China). The treated cells were seeded in 24-well plates and incubated with 50 μM EdU for 2 h at 37 °C. After being fixed with 4% paraformaldehyde, the cells were exposed to 100 μL of 1 ×Apollo® reaction cocktail and then incubated with 10 μg/mL DAPI solution (ready-to-use) to stain cell nuclei. Furthermore, to further investigate whether Linc00707 affects the proliferative capacity of MDA-MB-231 and MDA-MB-468 cells through affecting autophagy, CQ 10 μmol/L (Solarbio, Beijing, China) was added to MDA-MB-231 and MDA-MB-468 cells knockdown and overexpressing Linc00707 for 24 h, respectively, the proliferation rate of each group was detected by EdU. Images were captured using a fluorescence microscope (TCS SP8, Leica, Germany). The percentage of EdU-positive cells was defined as the proliferation rate.

For clone formation assay cells were seeded in six-well plates (800 cells/well) and cultured in complete medium supplemented with 10% fetal bovine serum for 2 weeks. Subsequently, the cells were stained with 0.1% Giemsa stain solution (Solarbio, Beijing, China) for 30 min and then washed once with PBS. The colonies were then counted if their diameter was greater than 1 mm. All of the experiments were repeated three times.

### Transwell migration and invasion assays

Migration and invasion assays were performed using Transwell chamber system (Corning, USA). For migration assay, 5 × 10^4^ cells were seeded in the upper chamber of an insert with serum-free media, and 0.6 ml culture media with 20% FBS were added out-side the chamber in the wells of the plate. For invasion assays, the upper chamber of the insert was pre-coated with Matrigel (Millipore Sigma) before plating cells. After incubation for 24 h, cells were fixed with ice-cold methanol for 1 h and then stained with Giemsa solution for 30 min. Furthermore, to further investigate whether Linc00707 affected the invasion and migration ability of MDA-MB-231 and MDA-MB-468 cells through affecting autophagy, CQ 10 μmol/L was added to MDA-MB-231 and MDA-MB-468 cells knockdown and overexpression of Linc00707 for 24 h, respectively, the invasion and migration rates of each group of cells were detected by the above steps. After rinsing with water and airing, migrating or invading cells were imaged and counted using a Leica DM2500 microscope. At least 5 random fields/wells were photographed and cells were counted for each field. Each experiment was performed in triplicate.

### Wound healing assay

We utilized a scratch assay to measure the cell migration ability. 6 × 10^5^ cells were seeded in each well of a 6 multi-well dish. After 24 h, the cell layer was scratched by a 10 μL pipette tip, plates were incubated at 37 °C, 5% CO_2_. Meanwhile, to further investigate whether Linc00707 affected the migratory ability of MDA-MB-231 and MDA-MB-468 cells through affecting autophagy, CQ 10 μmol/L was added to MDA-MB-231 and MDA-MB-468 cells knockdown and overexpressing Linc00707 for 24 h, respectively, the migration rate of each group of cells was detected by the above steps. The wound monolayer cells were photographed with time duration and the wound distance and rate were calculated using ImageJ (Bethesda, USA) software.

### Xenograft tumorigenesis

Animal experiments were approved by the Animal Experimental Research Ethics Committee of Weifang Medical University and conducted according to its guidelines. BALB/C nude mice (female, 6 weeks) were obtained from Vital River Laboratory Animal Technology Co. Ltd. (Beijing, China) and housed in isolation and ventilation cages under SPF (Specific pathogen Free) conditions in a climate-controlled room (25 ± 1.5 °C) with 12 h light/dark illumination cycle and 50 ± 10% humidity. 5 × 10^6^ MDA-MB-231 cells were transfected with lentivirus vector LV-sh-Linc00707 or LV-sh-con and lentivirus vector LV-oe-Linc00707 or LV-oe-NC, and then these cells were injected into the right dorsal flanks or the lateral tail vein of mice. After being weighed, mice that were overweight or underweight were removed and were randomly grouped (five for each group). Tumor growth was observed every three days and tumor volumes were calculated using the following formula: V (volume) = (length × width ^2^)/2. Mice were sacrificed at day 31 and at day 22, the tumor nodules, and lungs were extracted and stained by Hematoxylin and Eosin (HE) for assessment. Tumor nodules and metastatic loci were confirmed histologically. The number of metastatic loci was determined under microscope. The sections were scanned by two observers in a blinded manner.

### Immunohistochemistry (IHC) method

The expression of Ki67 was monitored using universal two-step detection kit (PV-9000, ZSGB-BIO, Beijing, China), in accordance with the manufacturer’s instructions.

### Immunofluorescence (IF) assay

The IF assay was carried out, as described previously [25]. Primary antibodies specific for anti-LC3 (1:200) (bs-8878R, Bioss) were applied. Fluorescence images were acquired.

### Western Blot analysis

The antibodies specific to human proteins LC3 (1:1000) (bs-24359R, Bioss), anti-SQSTM1/P62 (1:1000) (bs-24359R, Bioss) were purchased from Bioss Biotech (Bioss Biotechnology Co. Ltd. Beijing, China). The antibodies specific to human proteins p-PI3K (1:1000) (17366 S, Cell Signaling Technology), PI3K (1:1000) (4249 S, Cell Signaling Technology), Phospho-AKT^S473^ (1:2000,) (4058, Cell Signaling Technology), AKT (1:1000) (9272 S, Cell Signaling Technology), Phospho-mTOR^S2448^ (1:1000) (2971, Cell Signaling Technology), mTOR (1:1000) (2971 S, Cell Signaling Technology), p-p70s6k (1:1000) (9204 S, Cell Signaling Technology), p70s6k (1:1000) (9206 S, Cell Signaling Technology), p-4EBP1 (1:1000) (2855 S, Cell Signaling Technology), 4EBP1 (1:1000) (9644 S, Cell Signaling Technology) were purchased from cell signaling technology.

### StubRFP-sensGFP-LC3 lentivirus infection

StubRFP-sensGFP-LC3 lentivirus were purchased from Genechem (Shanghai, China) and were used to monitor autophagic flux. The MDA-MB-231 and MDA-MB-468 cell lines were infected with StubRFP-SensGFP-LC3 lentivirus, and after stable expression, they were transfected with sh-con and sh-Linc00707 plasmids. Furthermore, to further verify whether Linc00707 affects autophagy through the PI3K/AKT/mTOR signaling pathway, The PI3K inhibitor LY294002 10 μmol/L was added to the above cells. The StubRFP-SensGFP-LC3 fluorescence spots were photographed under laser confocal microscopy (TCS SP8, Leica, Germany), to monitor the progress of intracellular autophagic flux.

### Measurement of mitochondrial membrane potential (MMP)

The MMP level of TNBC cells and TNBC cells after the addition of the PI3K/AKT pathway inhibitor LY294002 10 μmol/L was monitored using JC-1 (C2006, Beyotime Biotechnology, Shanghai, China) in accordance with the manufacturer’s instructions. The excess dye was then replaced with fresh medium, and images were obtained by confocal microscopy (TCS SP8, Leica, Germany).

### Bioinformatics analyses

To predict the potential miRNAs binding with Linc00707, we used two online analysis tools: StarBase v3.0 (http://starbase.sysu.edu.cn/contact.php) and Lncbase (http://carolina.imis.athena-innovation.gr/diana_tools/web/index.php?r=lncbasev2/index-predicted).

### Biotin-labelled miRNA pull-down assay

The biotinylated-miR-423-5p probe was designed and synthesized by RiboBio (Guangzhou, China). MDA-MB-231 and MDA-MB-468 cells were transfected with biotin miR-423-5p probes and negative control probes respectively. Collect cell precipitation after 48 h of culture. The cell lysates were then incubated with streptavidin magnetic beads. The RNA complexes bound to the beads were then eluted and extracted for RT-qPCR analysis.

### Dual-luciferase assay

To construct luciferase reporter plasmids, the sequences of wild-type Linc00707, miR-423-5p binding site mutated Linc00707, wild-type MARCH2 3’UTR (MARCH2 WT), and miR-423-5p binding site mutated MARCH2 3’UTR were inserted into a luciferase reporter plasmid, and transfected into MDA-MB-231 cells. The luciferase activity was normalized to renilla luciferase activity after 48 h of transfection. Wild-type and mutant (Linc00707-MUT or MARCH2-MUT) Linc00707 and MARCH2 fragments were constructed and inserted downstream of the luciferase reporter gene in the reporter plasmid pRL-SV40 (GenePharma, Shanghai, China). HEK293T cells were seeded in 24-well plates and grown to 30% confluence 24 h before being transfected with the reporter plasmid using Lipofectamine 3000. Cells were also cotransfected with different combinations of plasmids harbouring the 3′-untranslated region (3′-UTR) of assayed genes (500 ng) and miRNA mimics or the negative control (NC; 10 nM final concentration). After 48 h, the activities of both firefly luciferase (LUC) and renilla luciferase (RLUC) were measured with a Dual-Luciferase Reporter System Kit (E1910, Promega, USA).

### Anti-AGO2 RNA immunoprecipitation (RIP)

RIP assays were performed using an RNA Immunoprecipitation Kit (Geneseed Biotech, Guangzhou, China). Approximately 1 × 10^7^ MDA-MB-231 and MDA-MB-468 cells were lysed in 1 ml of Buffer A supplemented with protease and RNase inhibitors. The cell lysates were then incubated with IgG and AGO2 antibody-coated protein A + G beads and rotated at 4 °C overnight. Subsequently, the immunoprecipitated RNAs levels of the assayed genes were measured by RT-qPCR.

### Statistical analysis

For in vivo experiments, animals were randomized. Randomization was not performed for all other experiments. All of the experiments were performed in biological triplicate unless otherwise specified. Correlation analyses between RNA expressions were performed using GraphPad Prism 8.0. Receiver-operating characteristic (ROC) curves and area under the curve (AUC) were used to assess the feasibility of the Linc00707 levels as a diagnostic for BC detection. Pearson’s correlation test assessed correlation analysis. *P* < 0.05 was considered statistically significant.

### Supplementary information

Supplementary figure legends

Supplementary figure 1

Supplementary figure 2

Supplementary figure 3

Supplementary figure 4

Supplementary figure 5

Original Data File

## Data Availability

TCGA breast cancer data (https://portal.gdc.cancer.gov) from the National Cancer Institute Genome Data Sharing Portal were used for this study. The coding proteins related to autophagy were downloaded from the HADb database (http://www.autophagy.lu/index.html). All data relevant to this study are included in the paper and/or supplementary material. The raw data of this article will be made available by the authors, without undue reservation. Further inquiries can be directed to the corresponding authors.
