# Supplementary material for: Linc00707 regulates autophagy and promotes the progression of triple negative breast cancer by activation of PI3K/AKT/mTOR pathway
Source: Cell Death Discov. 2024 Mar 14;10:138. doi: 10.1038/s41420-024-01906-7 (PMC10940671; doi:10.1038/s41420-024-01906-7)
Supplement: Supplementary file 1 — Supplementary figure legends [file 41420_2024_1906_MOESM1_ESM.docx]

**Supplement Figure 1** **(A, B)** Colony formation demonstrated the proliferation abilities of MDA-MB-231 and MDA-MB-468 cells. **(C)** Wound healing assay in MDA-MB-231 cells transfected with sh-con or sh-Linc00707 #1 or sh-Linc00707 #2, oe-NC or oe-Linc00707. Scale bar of bright field: 500μm. **(D)** Wound healing assay in MDA-MB-468 cells transfected with sh-con or sh-Linc00707 #1 or sh-Linc00707 #2, oe-NC or oe-Linc00707. Scale bar of bright field: 200μm. All experiments were repeated independently three times. Data are presented as means ± standard deviation. ** P*<0.05, *** P*<0.01, **** P*<0.001.

.

**Supplement Figure 2** **(A)** The nude mice were subcutaneously injected with 5×10^6^ MDA-MB-231 cells stably transfected with LV-oe-NC or LV-oe-Linc00707 cells. A ruler was used to indicate the size of the tumors. The tumor weight with different cells was shown. **(B)** Immunohistochemistry for Ki67 detection in LV-oe-NC and LV-oe-Linc00707 group. **(C)** Representative images of the lung obtained from nude mice and numbers of lung metastasis lesions were calculated. **(D)** HE staining of lung tissues were used to detect the metastasis nodules. Data are presented as means ± standard deviation. ** P*<0.05, *** P*<0.01, **** P*<0.001.

**Supplement Figure 3** **(A)** The differentially expressed proteins related to autophagy were obtained from TCGA and HADb databases. **(B)** Spearman correlation analysis of the expression of Linc00707 and the expression levels of 10 autophagy-related proteins.

**Supplement Figure 4** (A) The MDA-MB-231 and MDA-MB-468 cell lines stably expressing stubRFP-sensGFP-LC3 were transfected with sh-con and sh-Linc00707 plasmids, and LY294002 was added, and StubRFP-SensGFP-LC3 fluorescent spots were observed by the Fluorescence microscope. GFP represented autophagosomes, RFP represented the total number of autophagosomes and autolysosomes, yellow puncta in the merged image represented the flow rate of autophagic flow from autophagosomes to autolysosomes. Scale bar: 50 μm. **(B)** Changes in mitochondrial membrane potential of MDA-MB-231 and MDA-MB-468 after addition of LY294002 in the sh-con and sh-Linc00707 groups were monitored by JC-1. Green fluorescence: JC-1 monomer, red fluorescence: JC-1 aggregates. Scale bar: 50 μm.

**Supplement Figure 5 (A, B)** Typical images of the proliferation abilities of MDA-MB-231 and MDA-MB-468 cells by Colony formation. **(C)** Colony statistical analysis of the cell proliferation ability in MDA-MB-231 and MDA-MB-468 cells. **(D)** Wound healing assay analysis of the cell migration ability of MDA-MB-231 and MDA-MB-468 cells. **(E, F)** Typical images of Wound healing assay in MDA-MB-231 and MDA-MB-468 cells transfected with sh-con+miR-NC, sh-Linc00707 #1+miR-NC, sh-Linc00707 #2+miR-NC, sh-con+miR-423-5p inhibitor, sh-Linc00707 #1+miR-423-5p inhibitor, sh-Linc00707 #2+miR-423-5p inhibitor. Scale bar: 100 μm. All experiments were repeated independently three times. Data are presented as means ± standard deviation. * *P*<0.05, *** P*<0.01, **** P*<0.001.
