# Supplementary figures and images for: Linc00707 regulates autophagy and promotes the progression of triple negative breast cancer by activation of PI3K/AKT/mTOR pathway
Source: Cell Death Discov. 2024 Mar 14;10:138. doi: 10.1038/s41420-024-01906-7 (PMC10940671; doi:10.1038/s41420-024-01906-7)

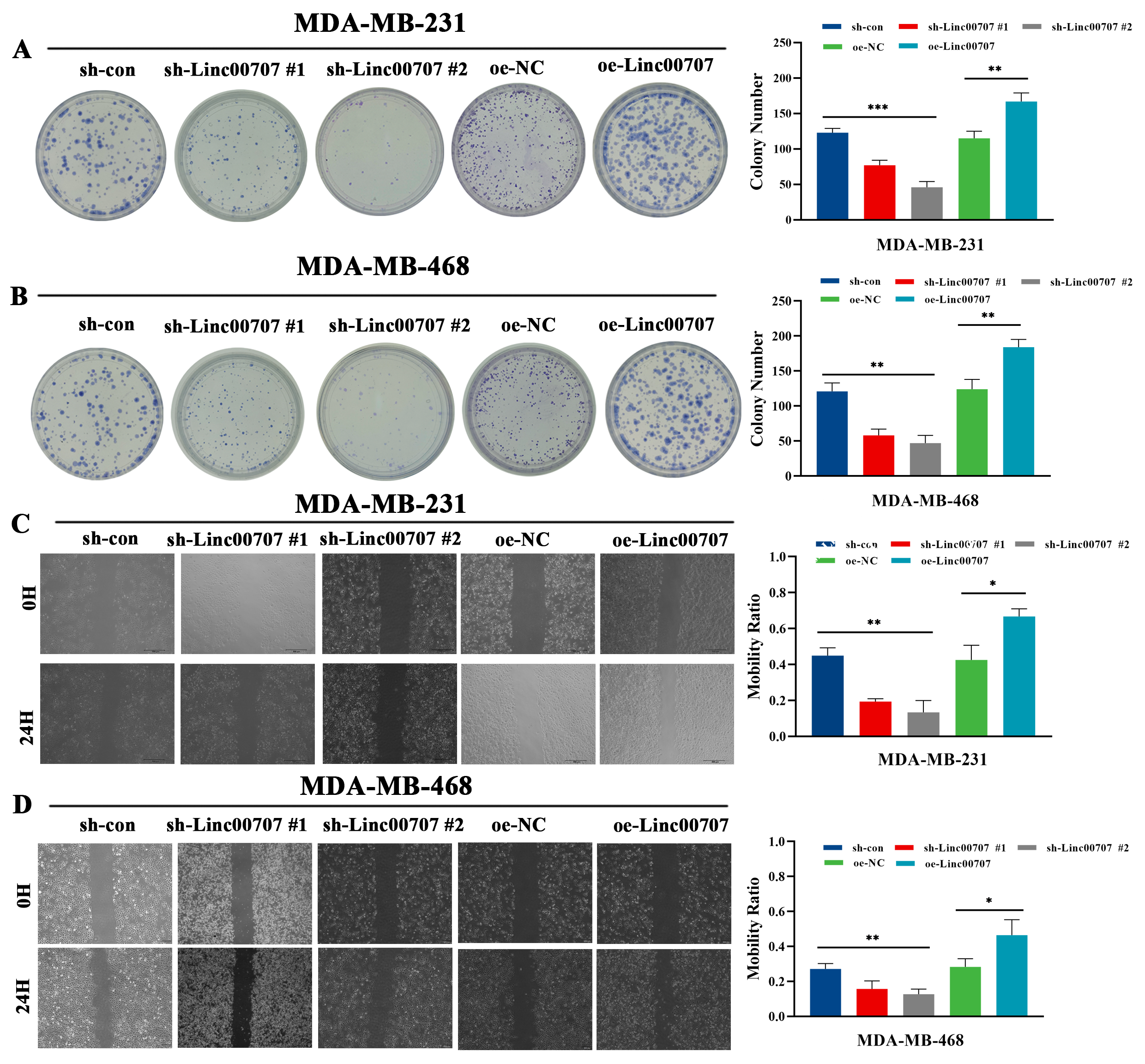

Supplement: Supplementary file 2 — Supplementary figure 1 [file 41420_2024_1906_MOESM2_ESM.tif]

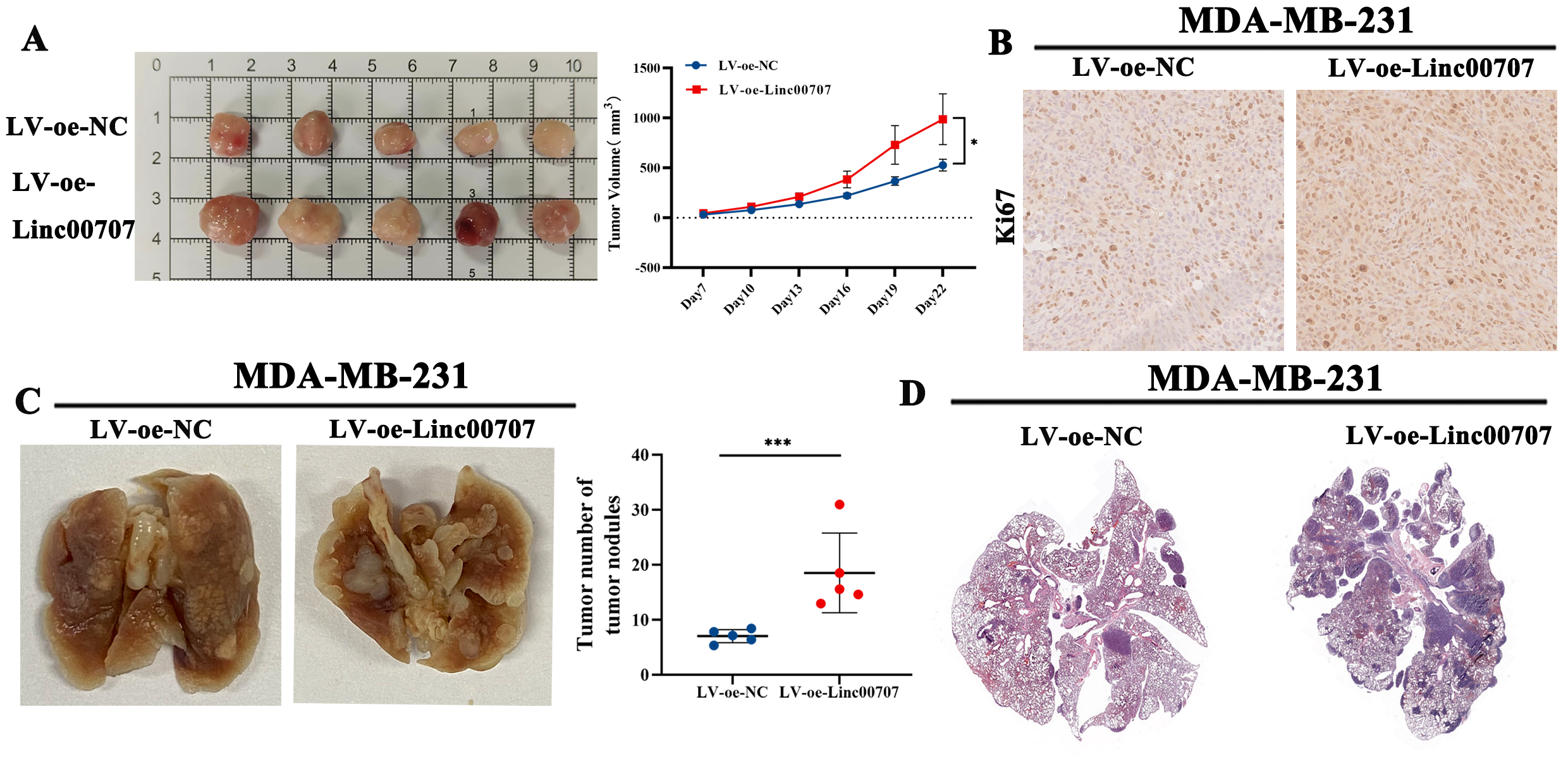

Supplement: Supplementary file 3 — Supplementary figure 2 [file 41420_2024_1906_MOESM3_ESM.tif]

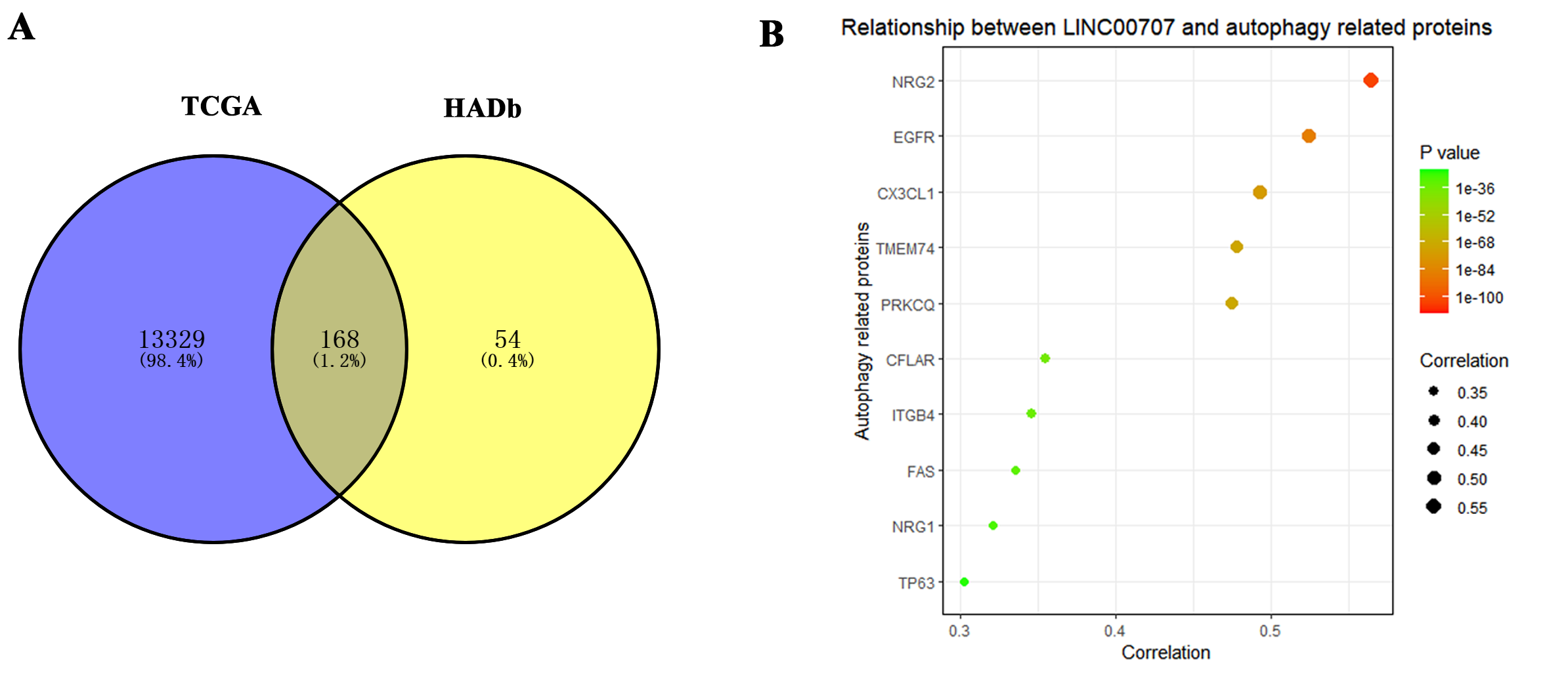

Supplement: Supplementary file 4 — Supplementary figure 3 [file 41420_2024_1906_MOESM4_ESM.tif]

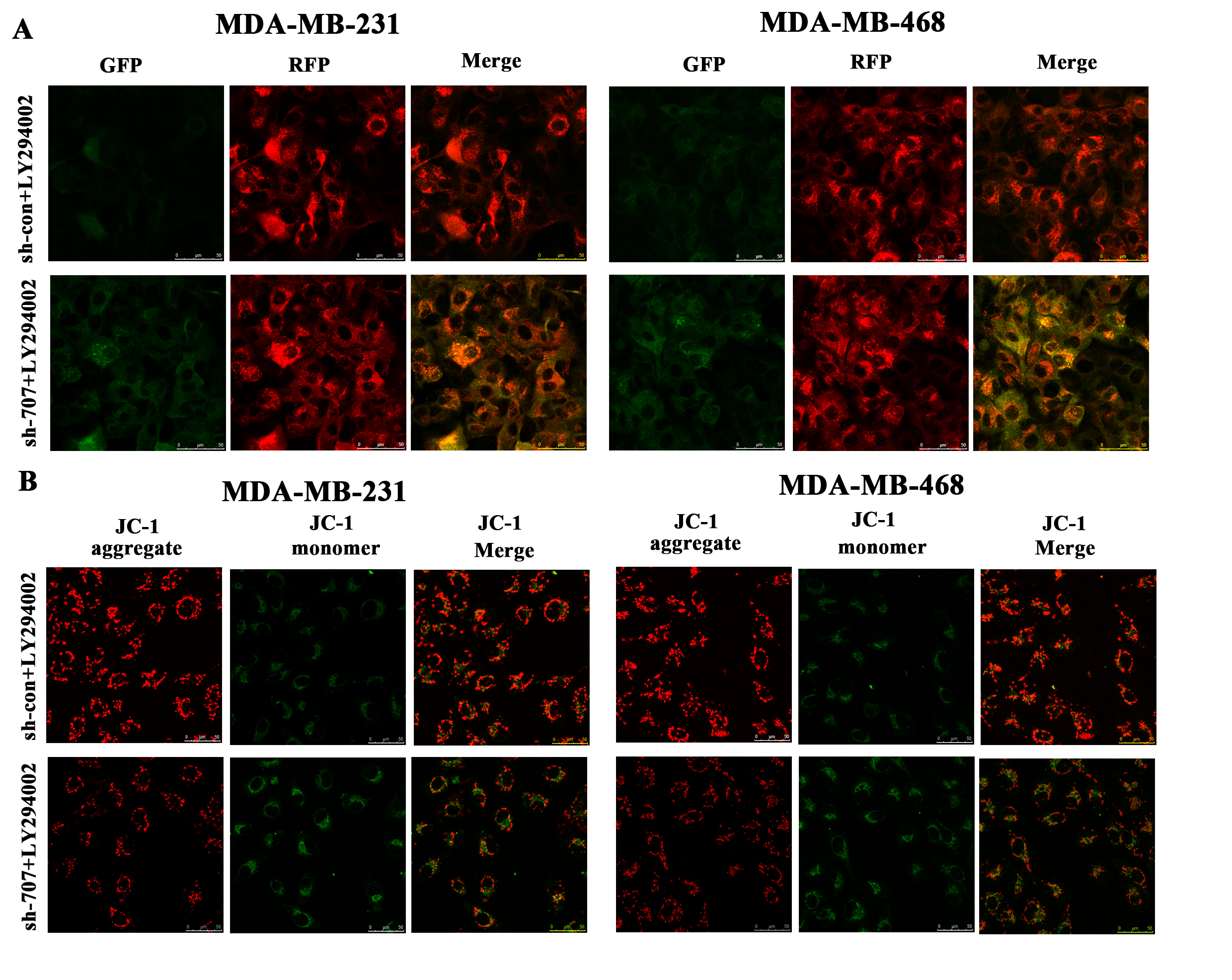

Supplement: Supplementary file 5 — Supplementary figure 4 [file 41420_2024_1906_MOESM5_ESM.tif]

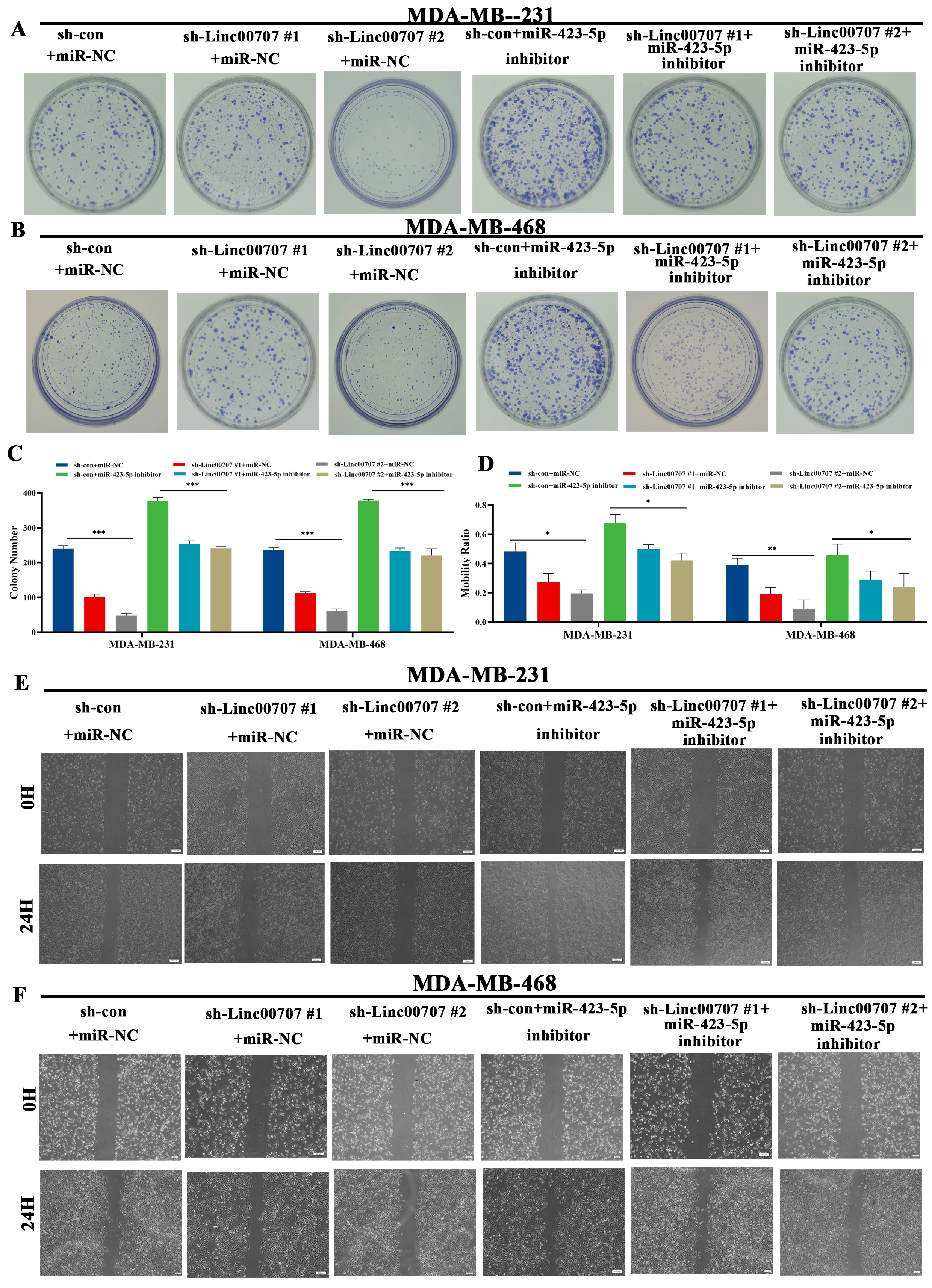

Supplement: Supplementary file 6 — Supplementary figure 5 [file 41420_2024_1906_MOESM6_ESM.tif]
