# Supplementary material for: Linc00707 regulates autophagy and promotes the progression of triple negative breast cancer by activation of PI3K/AKT/mTOR pathway
Source: Cell Death Discov. 2024 Mar 14;10:138. doi: 10.1038/s41420-024-01906-7 (PMC10940671; doi:10.1038/s41420-024-01906-7)

FIG1G-231FISH

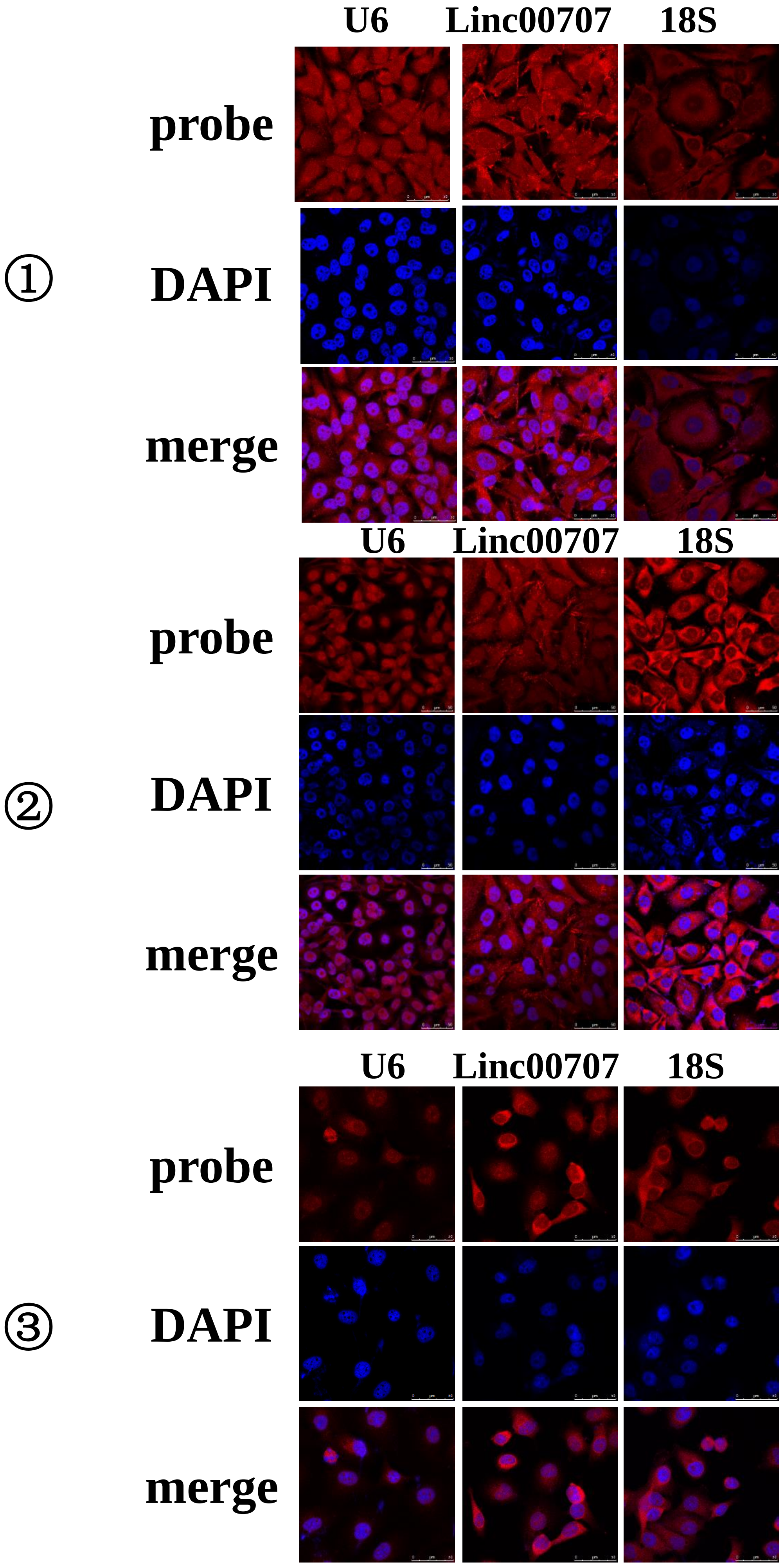

FIG1G-468FISH

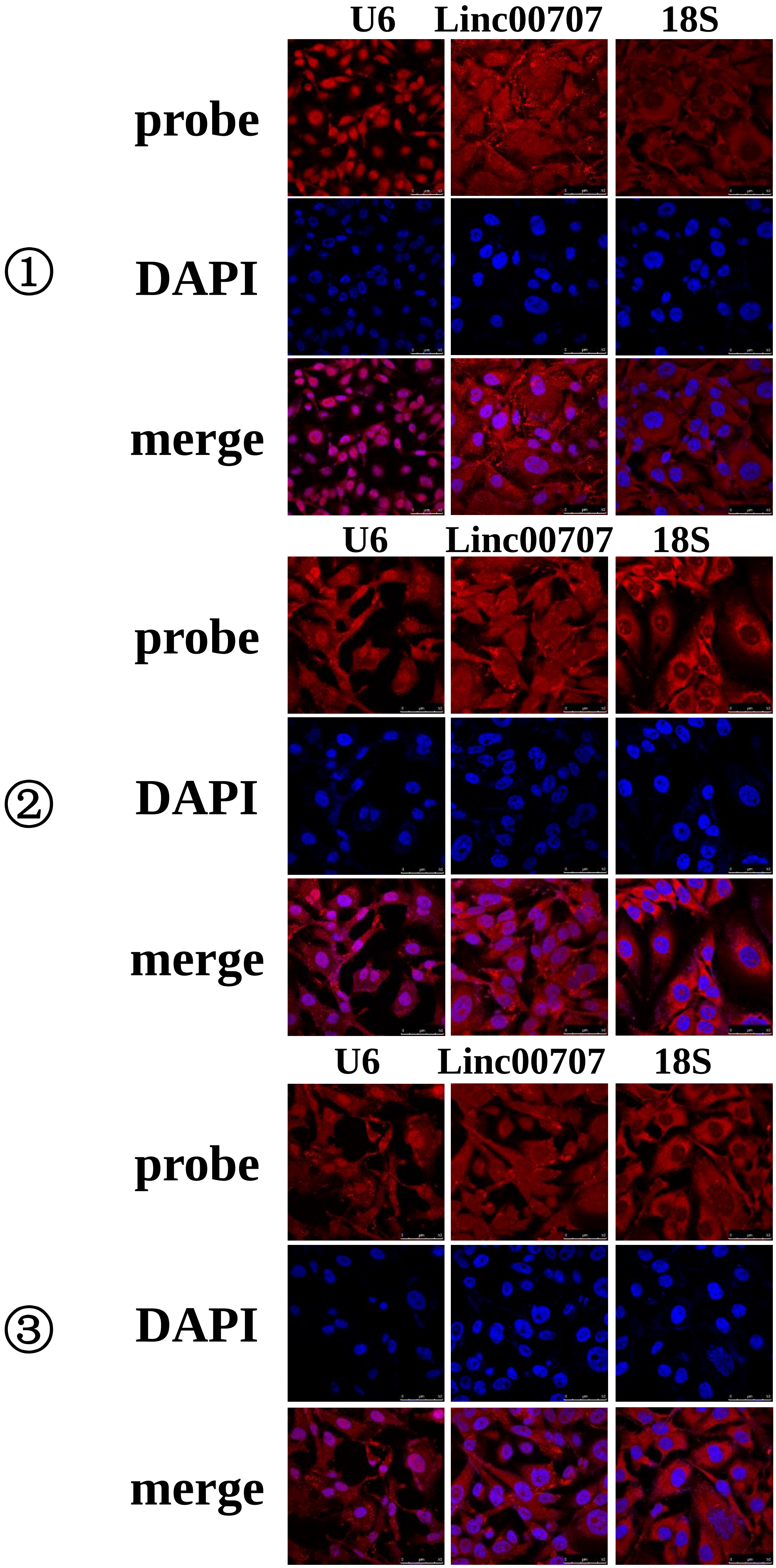

FIG2E-231EdU

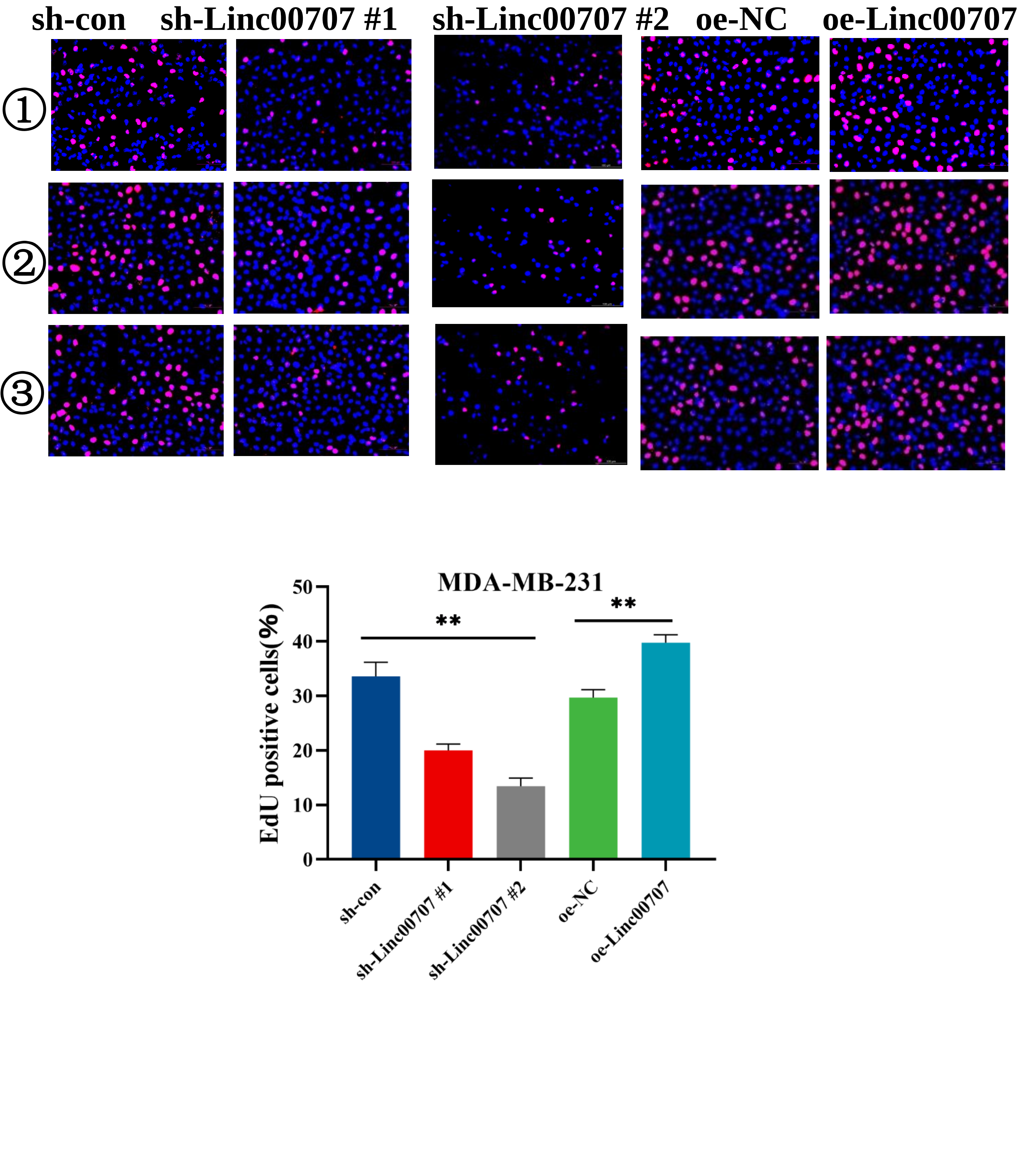

FIG2F-468EdU

sh-con　sh-Linc00707 #1　sh-Linc00707 #2　oe-NC　oe-Linc00707

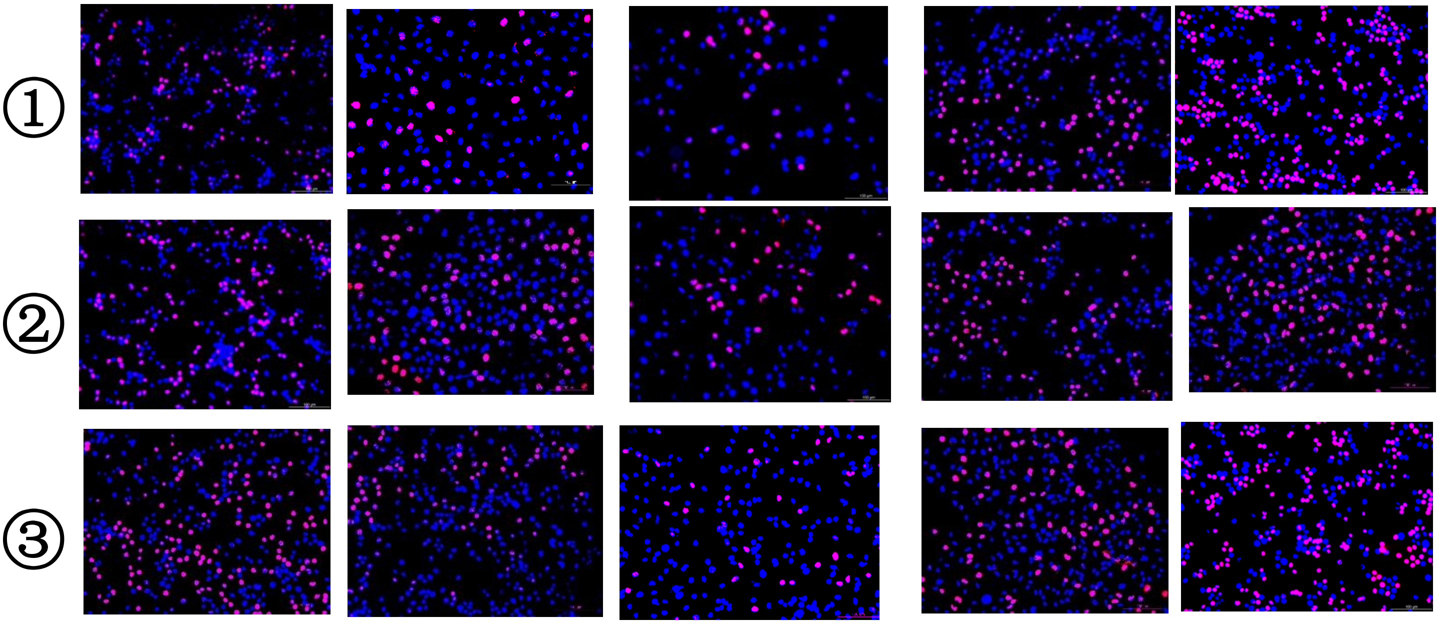

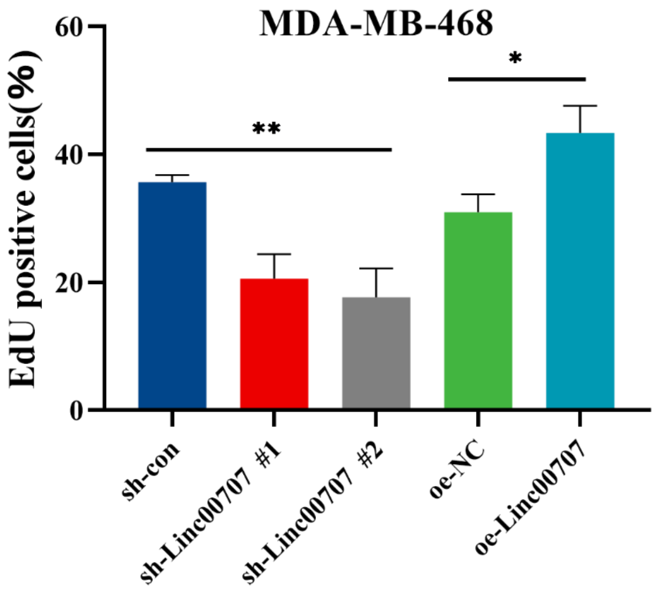

FIG2G

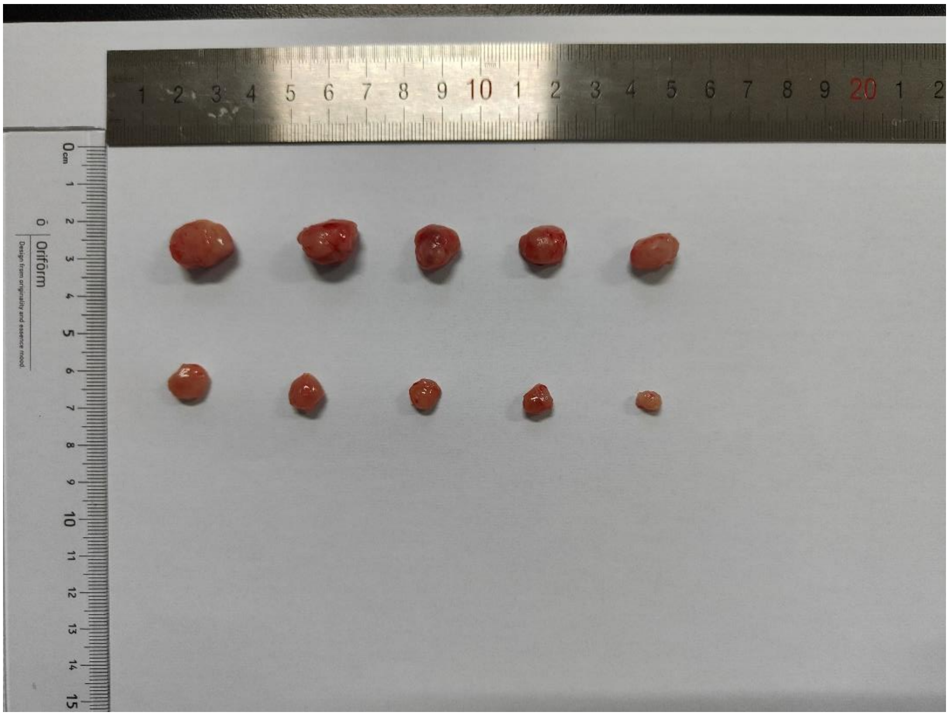

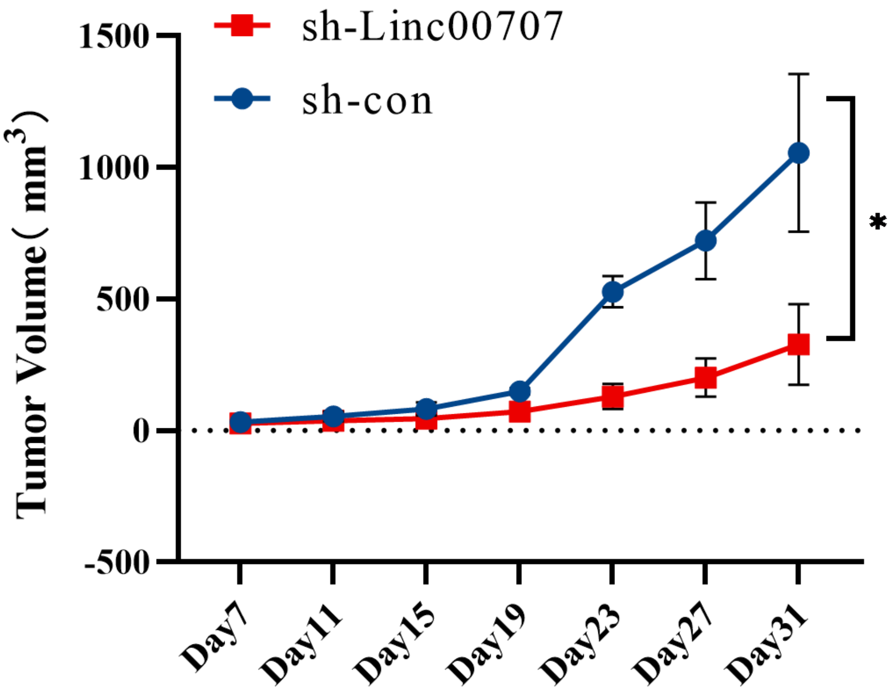
sh-Linc00707
sh-con
Tumor Volume（mm3）
1500
1000
500
0
-500
*
Day7
Day11
Day15
Day19
Day23
Day27
Day31

FIG2H-ki67

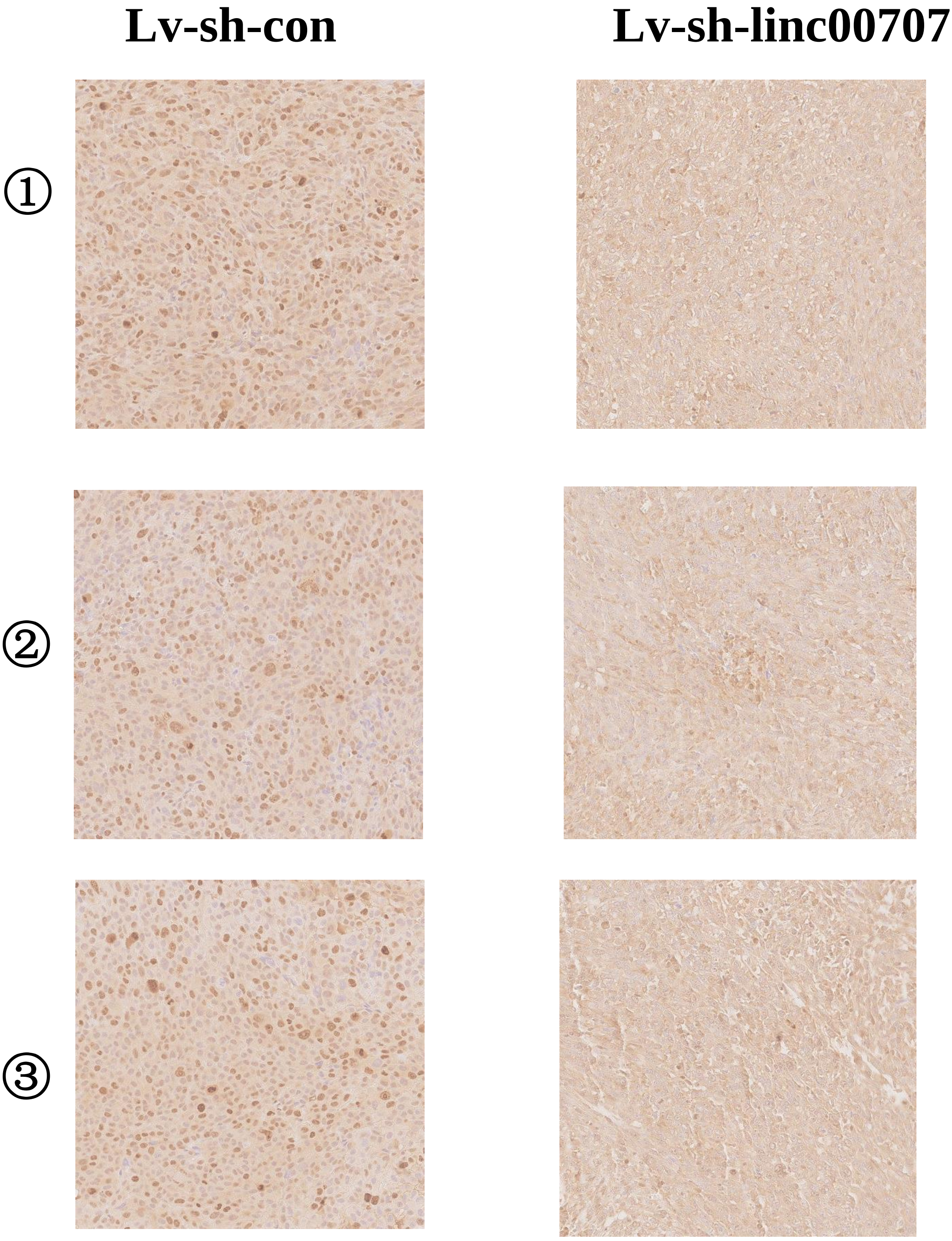

FIG2I-231TRANSWELL

Invasion

sh-con sh-Linc00707 #1 sh-Linc00707 #2 oe-NC oe-Linc00707

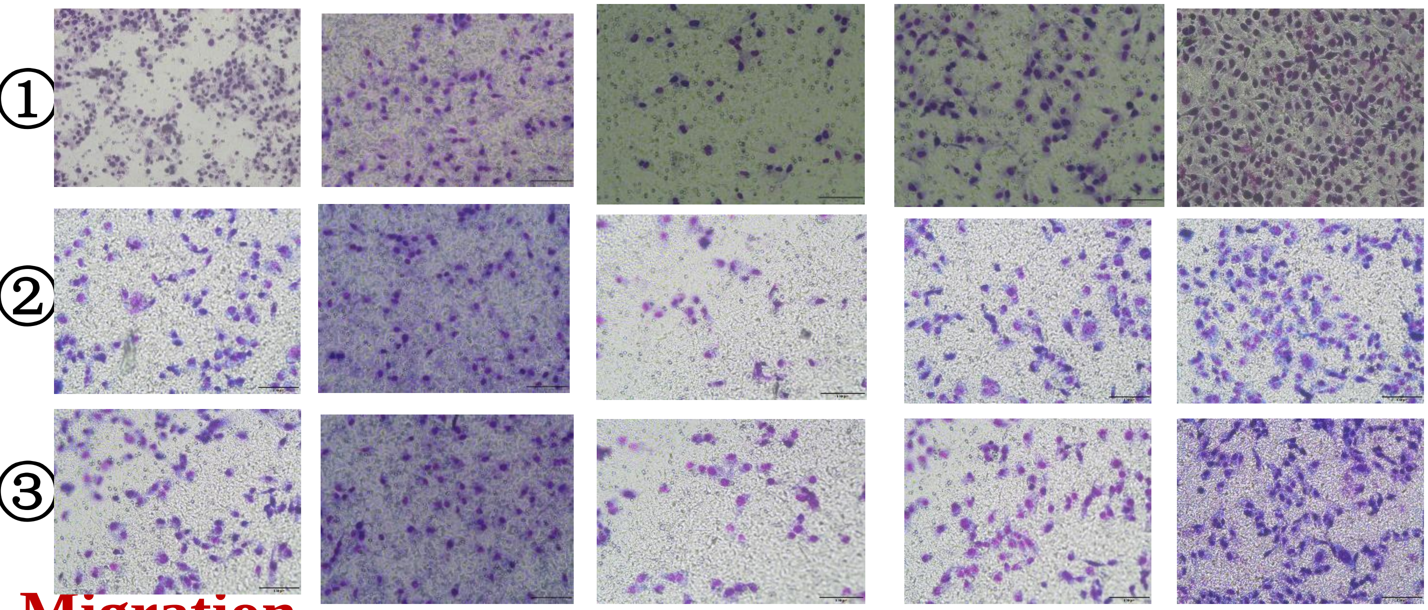

Migration

sh-con sh-Linc00707 #1 sh-Linc00707 #2 oe-NC oe-Linc00707

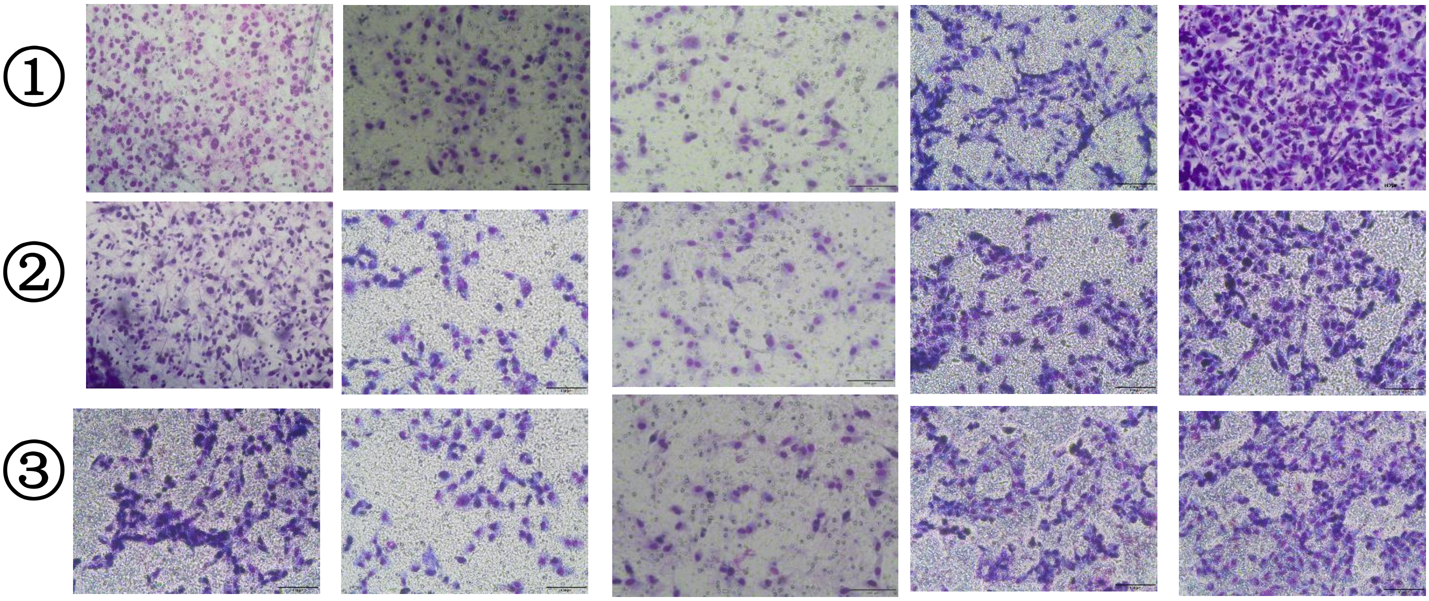

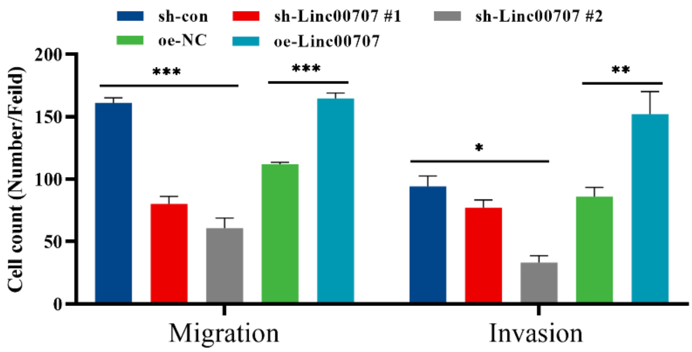

FIG2J-468TRANSWELL

Invasion

sh-con sh-Linc00707 #1 sh-Linc00707 #2 oe-NC oe-Linc00707

① ② ③

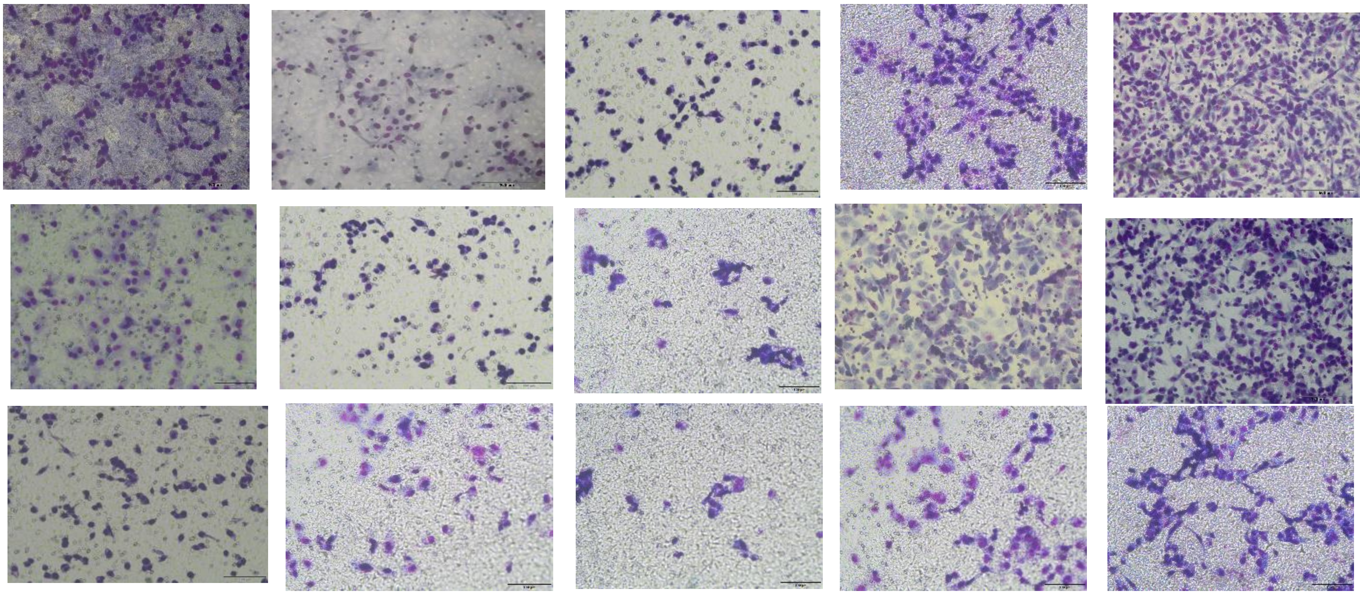

Migration

sh-con sh-Linc00707 #1 sh-Linc00707 #2 oe-NC oe-Linc00707

① ② ③

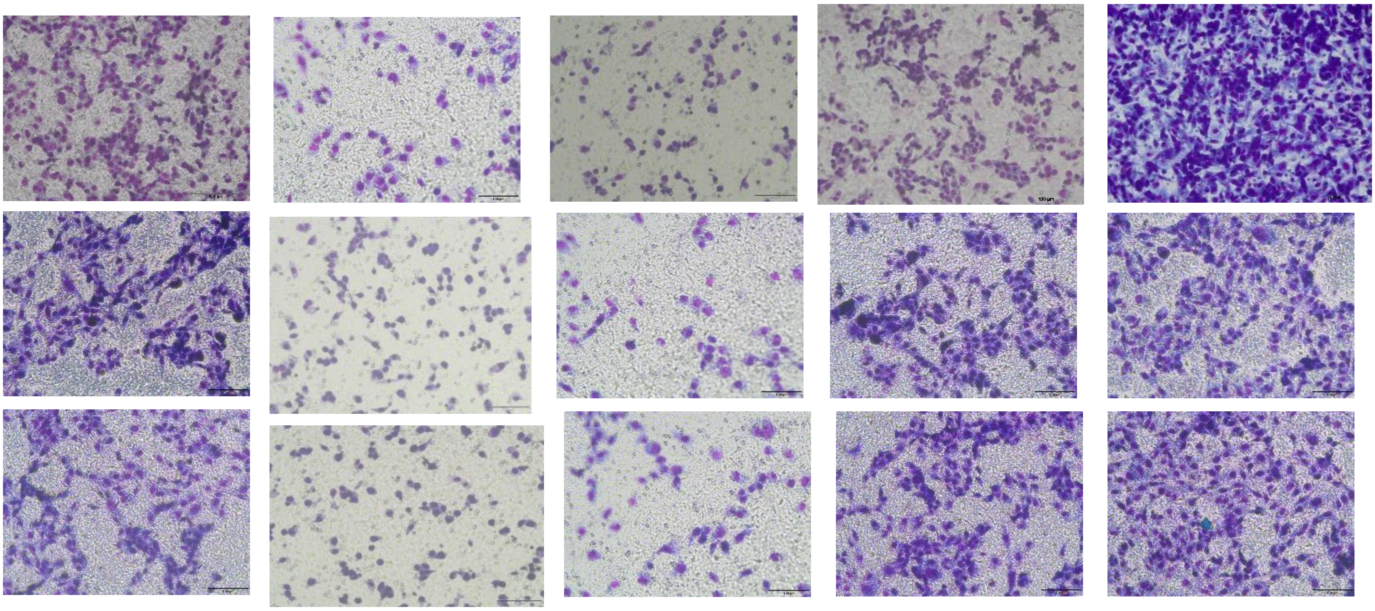

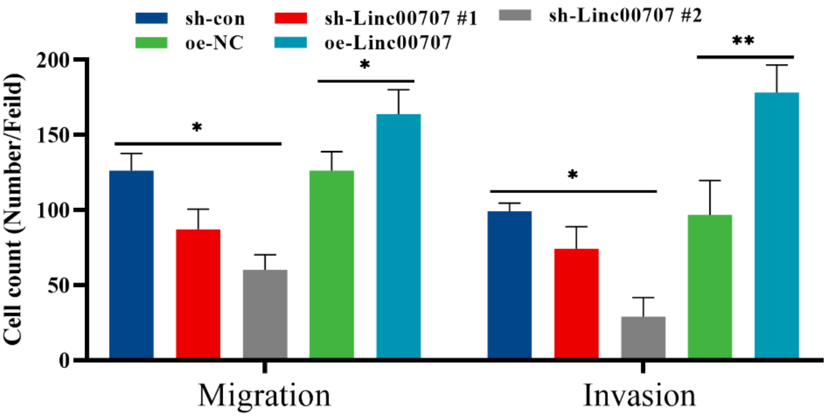

FIG2K

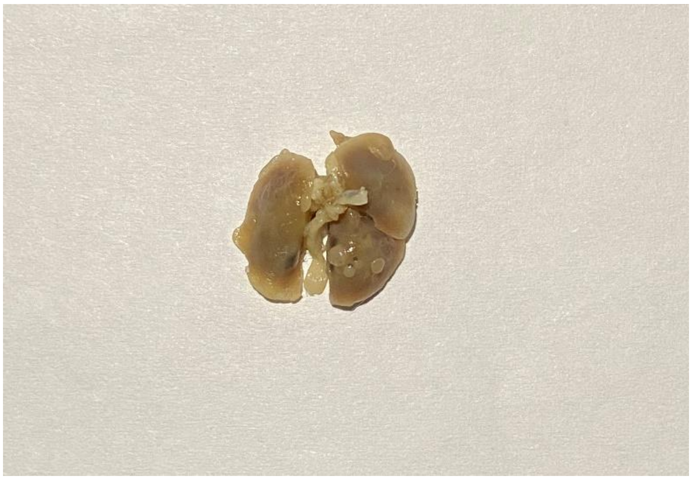

Sh-con

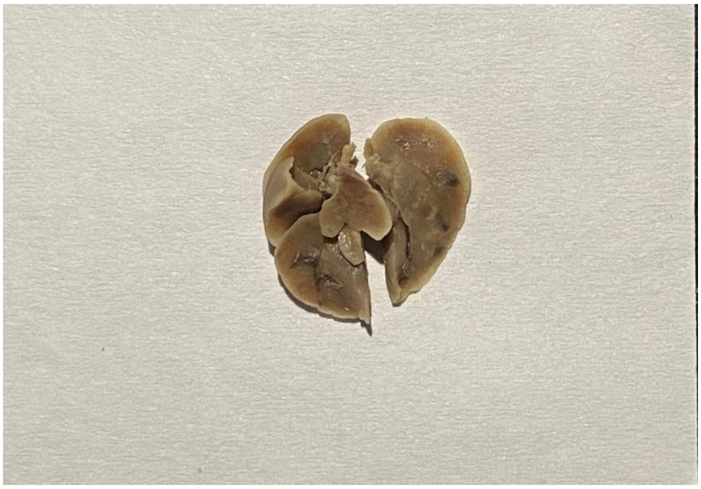

Sh-Linc00707

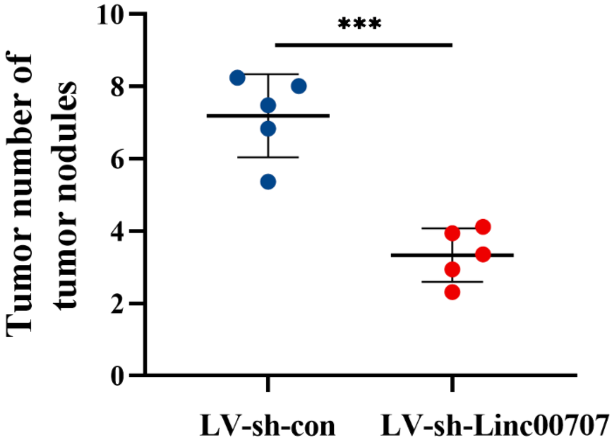

FIG2L

Lv-sh-con Lv-sh-linc00707

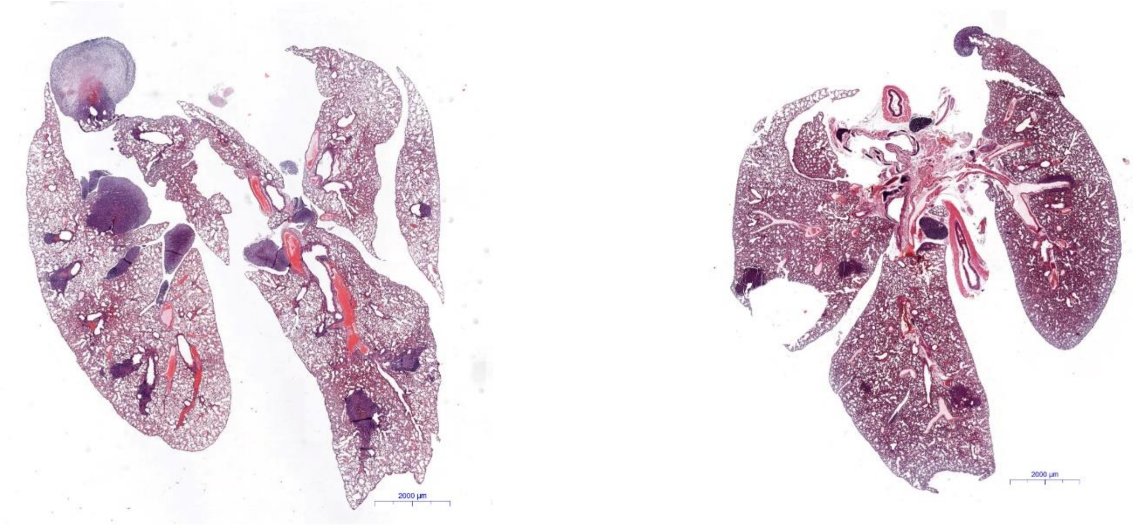

FIG3A-LC3

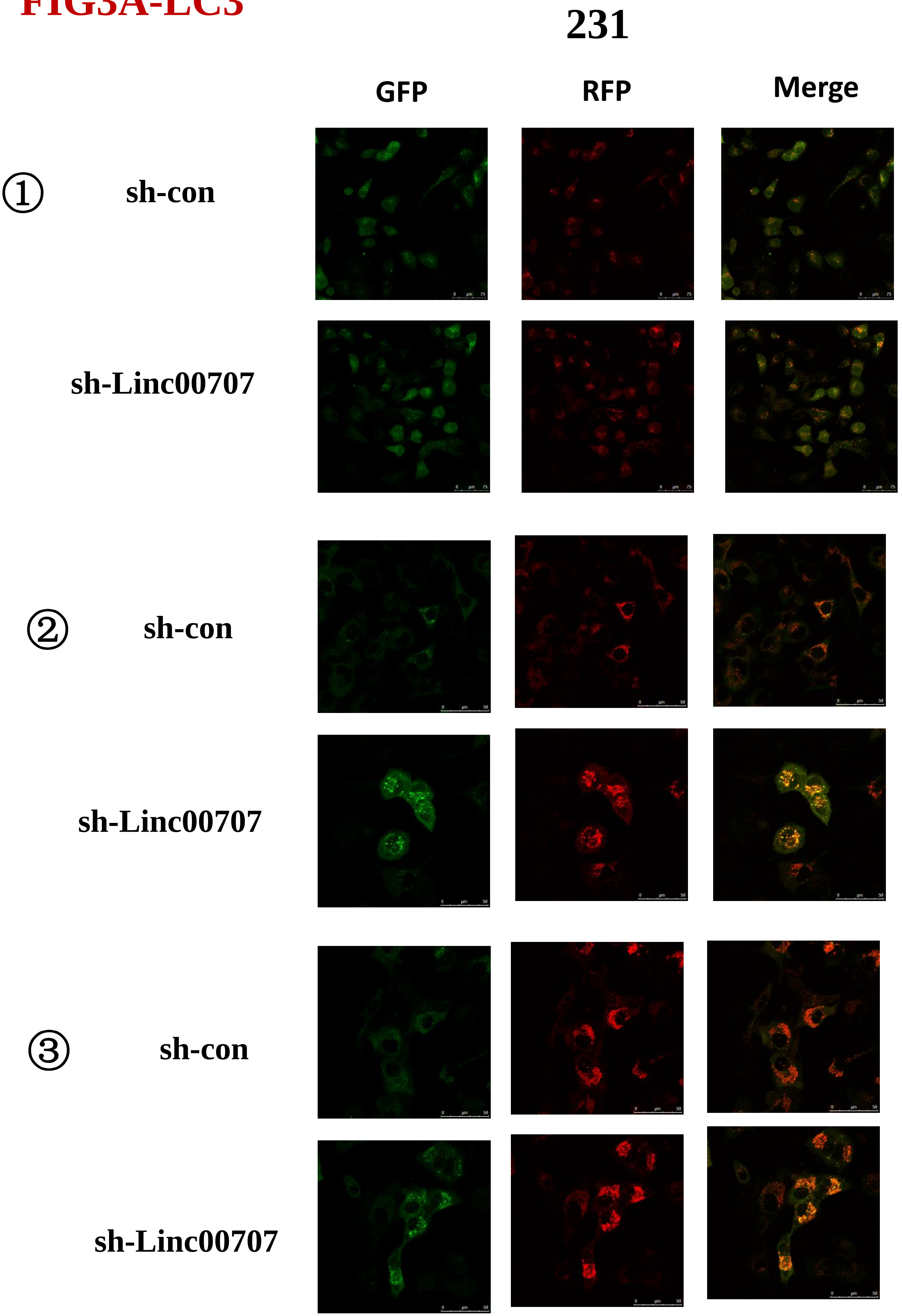
231
GFP
RFP
Merge
① sh-con
sh-Linc00707
② sh-con
sh-Linc00707
③ sh-con
sh-Linc00707

FIG3A-LC3

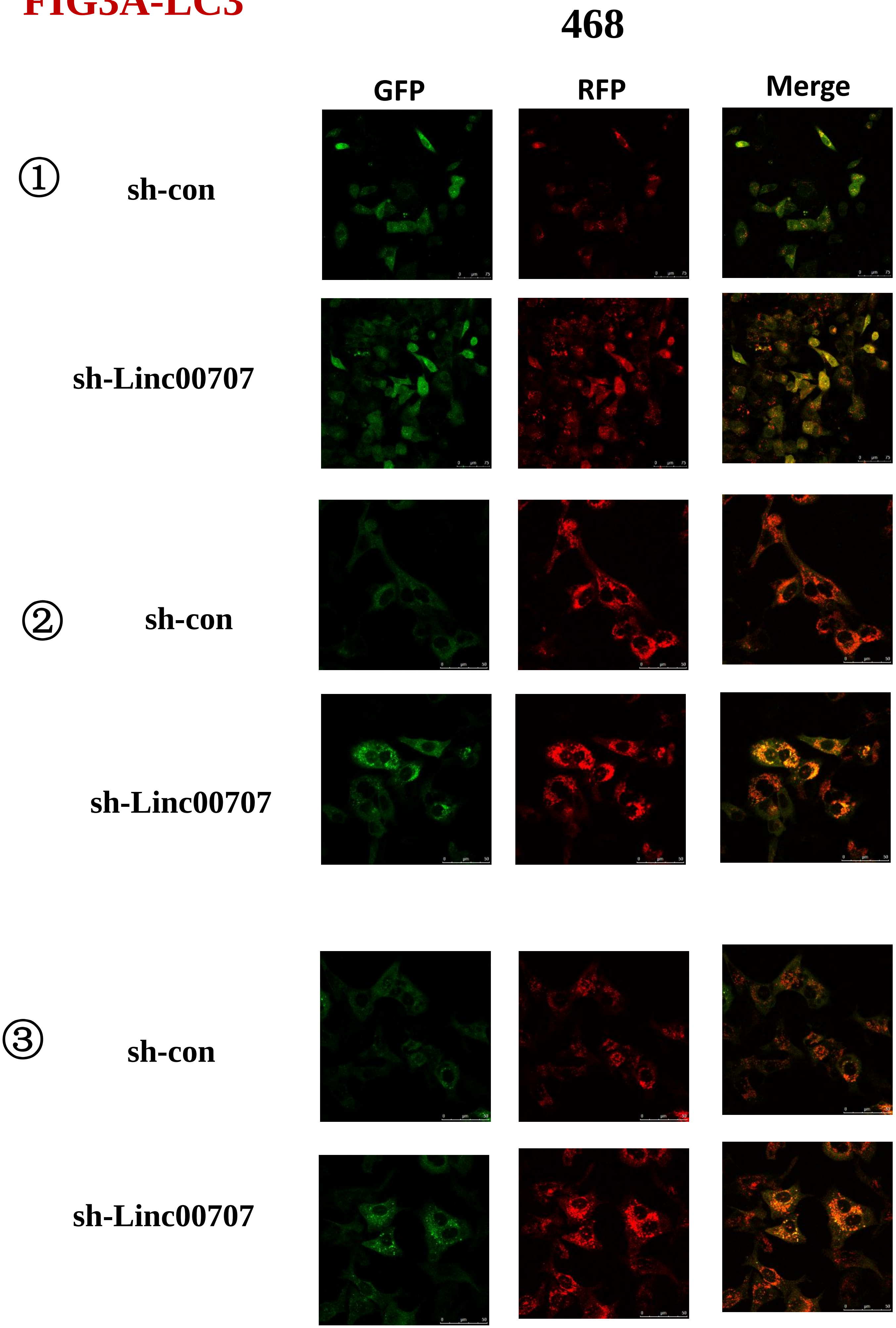
468
GFP
RFP
Merge
① sh-con
sh-Linc00707
② sh-con
sh-Linc00707
③ sh-con
sh-Linc00707

FIG3B-JC-1

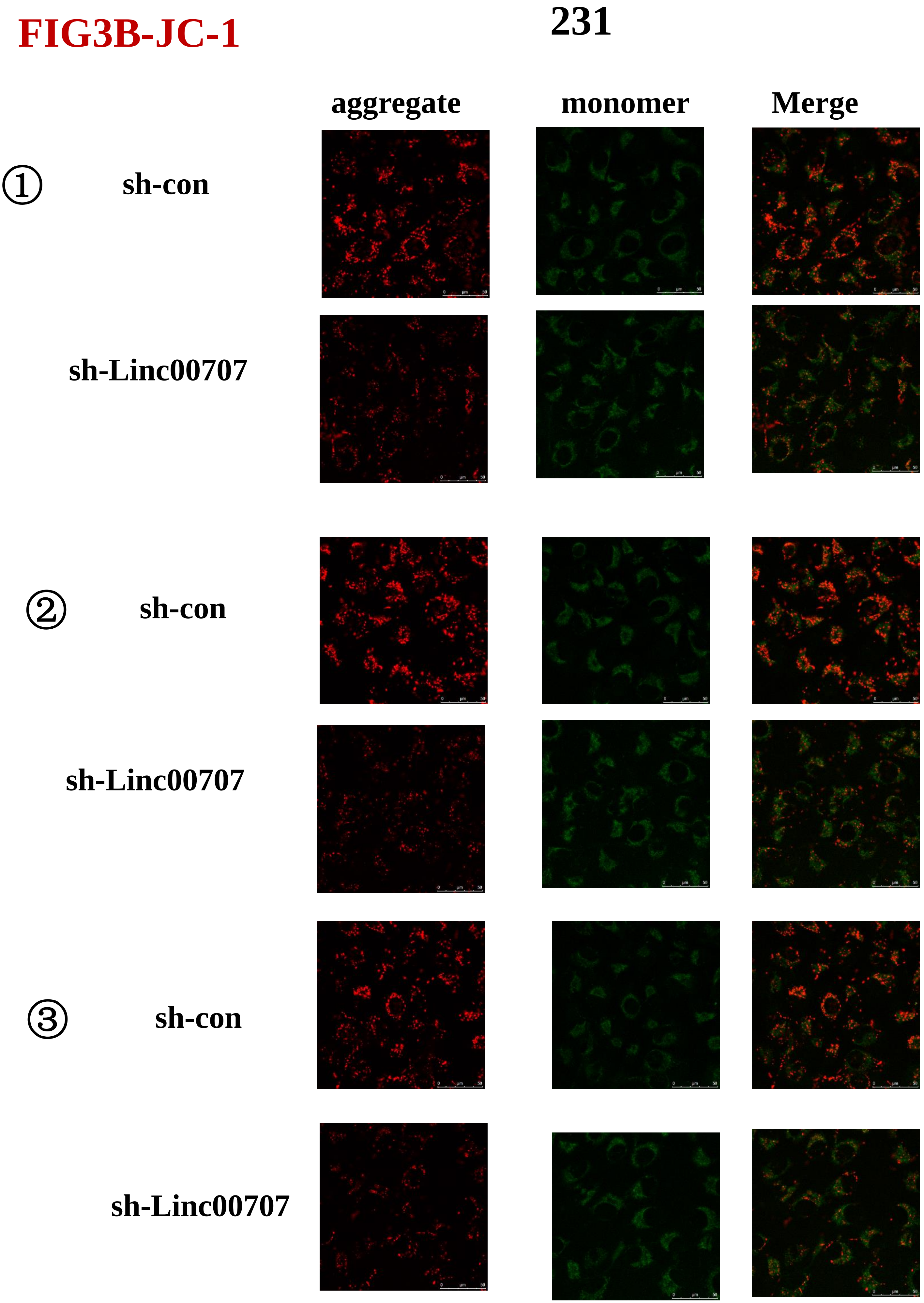
231
aggregate
monomer
Merge
① sh-con
sh-Linc00707
② sh-con
sh-Linc00707
③ sh-con
sh-Linc00707

FIG3B-JC-1

468

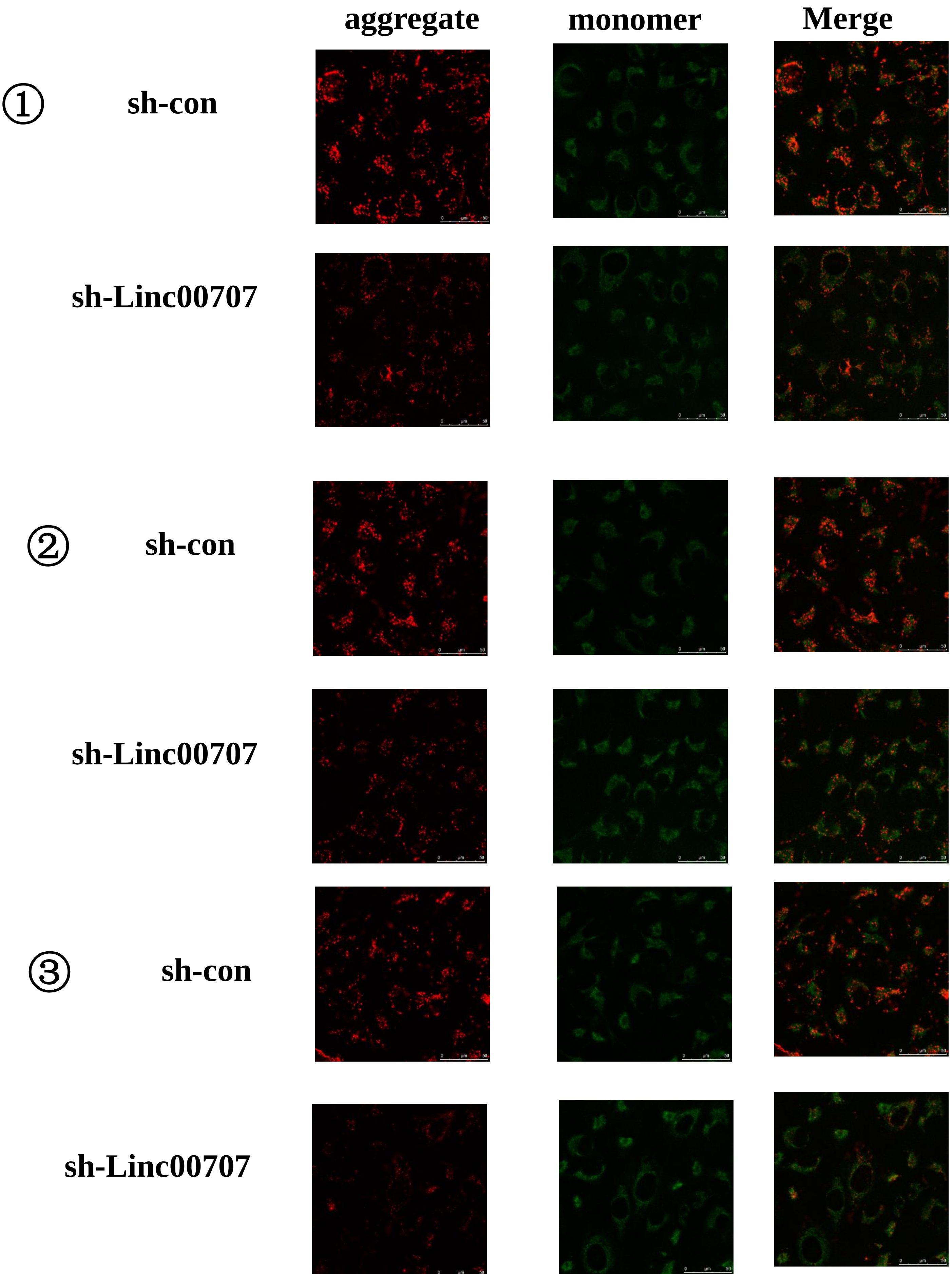

FIG3C-D

①

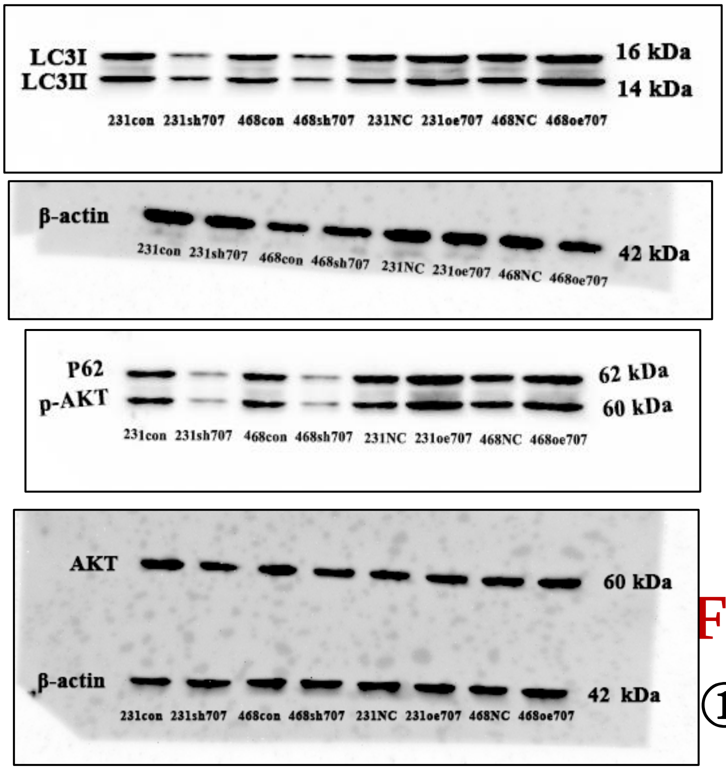

②

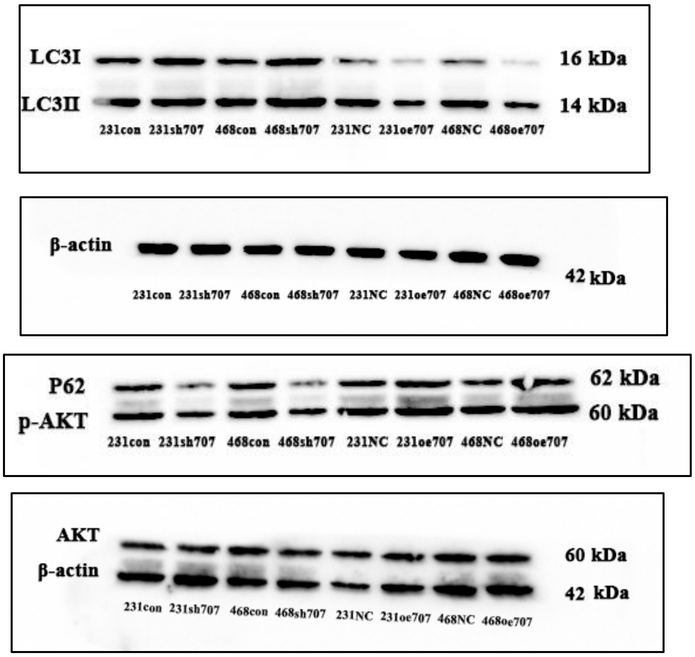

③

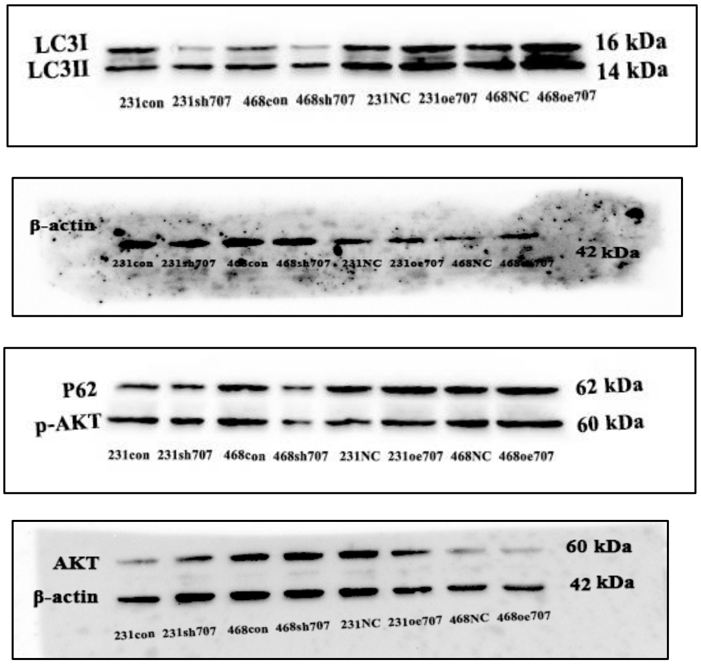

FIG3D

①

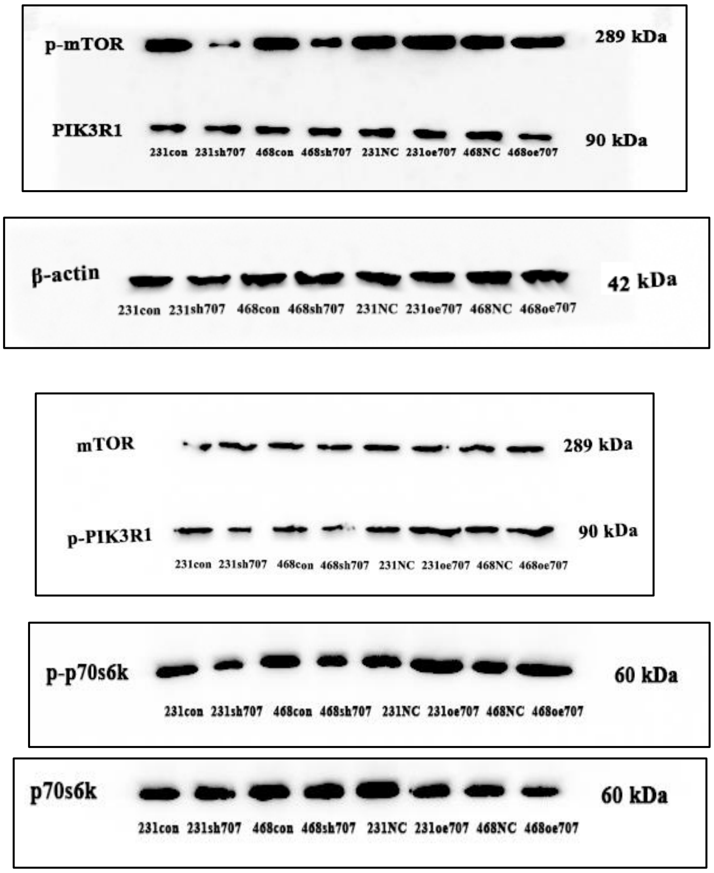

FIG3E

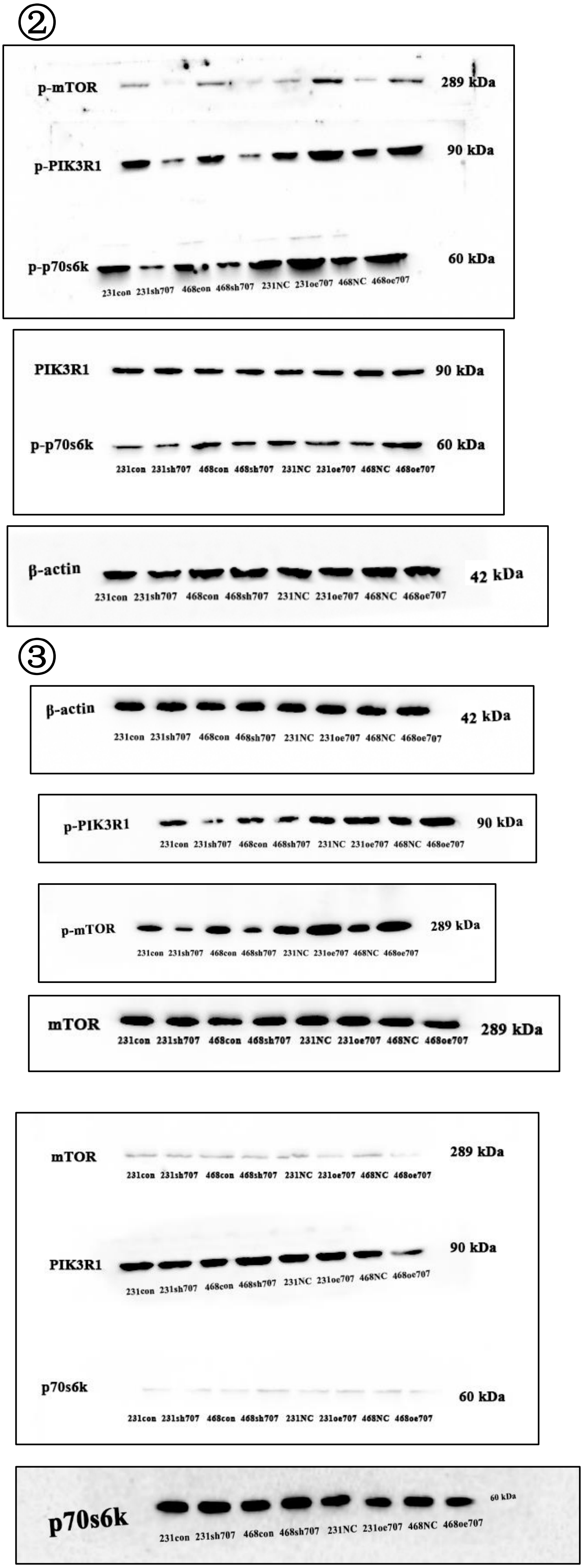
②
p-mTOR
289 kDa
p-PIK3R1
90 kDa
p-p70s6k
60 kDa
231con 231sh707 468con 468sh707 231NC 231oe707 468NC 468oe707
PIK3R1
90 kDa
p-p70s6k
60 kDa
231con 231sh707 468con 468sh707 231NC 231oe707 468NC 468oe707
β-actin
42 kDa
231con 231sh707 468con 468sh707 231NC 231oe707 468NC 468oe707
③
β-actin
42 kDa
231con 231sh707 468con 468sh707 231NC 231oe707 468NC 468oe707
p-PIK3R1
90 kDa
231con 231sh707 468con 468sh707 231NC 231oe707 468NC 468oe707
p-mTOR
289 kDa
231con 231sh707 468con 468sh707 231NC 231oe707 468NC 468oe707
mTOR
289 kDa
231con 231sh707 468con 468sh707 231NC 231oe707 468NC 468oe707
mTOR
289 kDa
231con 231sh707 468con 468sh707 231NC 231oe707 468NC 468oe707
PIK3R1
90 kDa
231con 231sh707 468con 468sh707 231NC 231oe707 468NC 468oe707
p70s6k
60 kDa
231con 231sh707 468con 468sh707 231NC 231oe707 468NC 468oe707
p70s6k
60 kDa
231con 231sh707 468con 468sh707 231NC 231oe707 468NC 468oe707

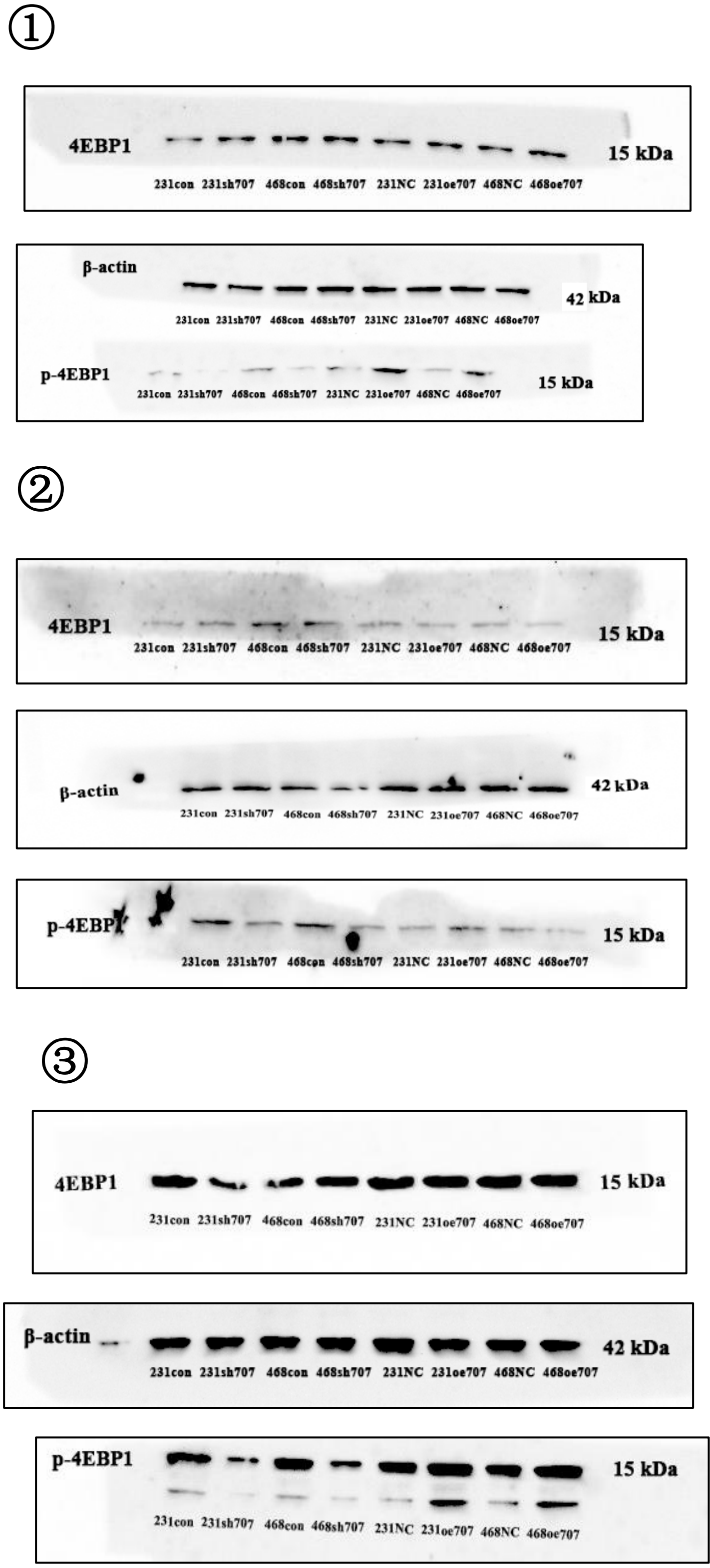
①
4EBP1
15 kDa
231con 231sh707 468con 468sh707 231NC 231oe707 468NC 468oe707
β-actin
42 kDa
231con 231sh707 468con 468sh707 231NC 231oe707 468NC 468oe707
p-4EBP1
15 kDa
231con 231sh707 468con 468sh707 231NC 231oe707 468NC 468oe707
②
4EBP1
15 kDa
231con 231sh707 468con 468sh707 231NC 231oe707 468NC 468oe707
β-actin
42 kDa
231con 231sh707 468con 468sh707 231NC 231oe707 468NC 468oe707
p-4EBP1
15 kDa
231con 231sh707 468con 468sh707 231NC 231oe707 468NC 468oe707
③
4EBP1
15 kDa
231con 231sh707 468con 468sh707 231NC 231oe707 468NC 468oe707
β-actin
42 kDa
231con 231sh707 468con 468sh707 231NC 231oe707 468NC 468oe707
p-4EBP1
15 kDa
231con 231sh707 468con 468sh707 231NC 231oe707 468NC 468oe707

4A-231EDU

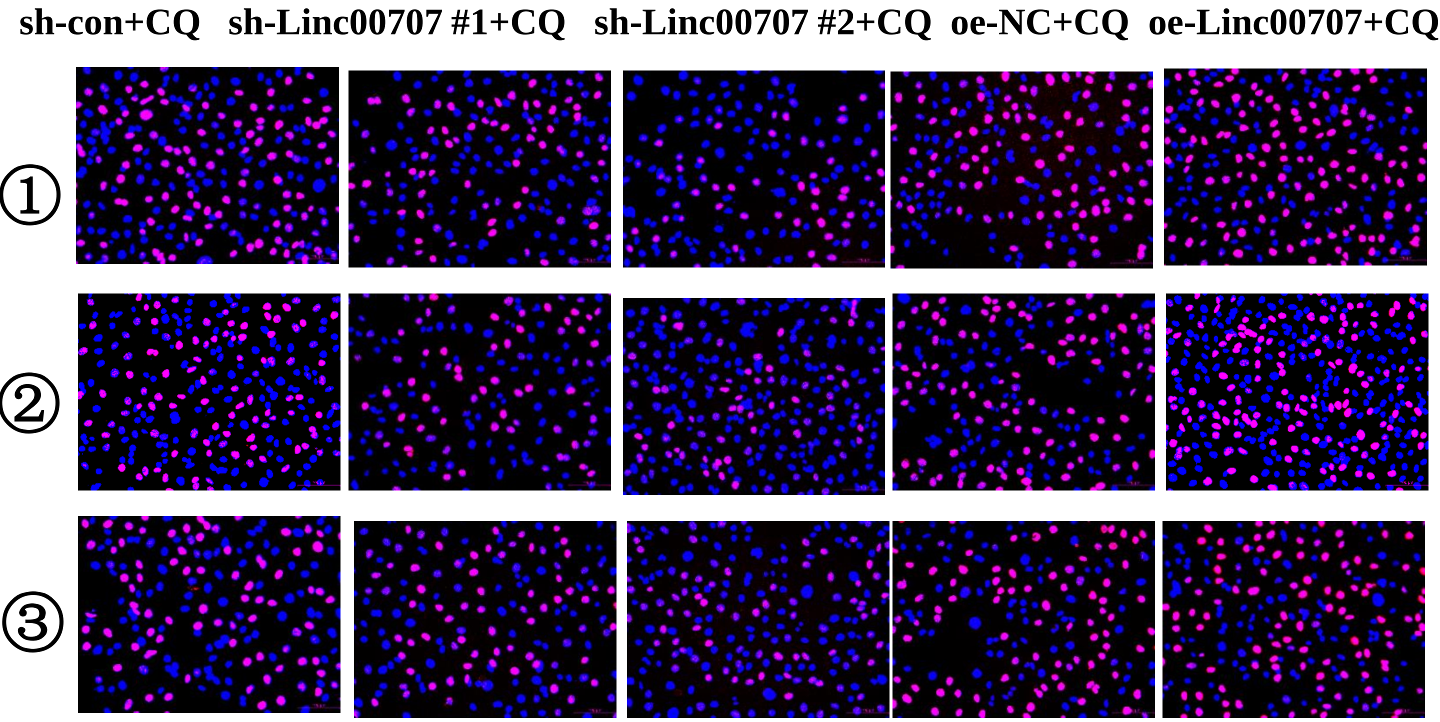
sh-con+CQ
sh-Linc00707 #1+CQ
sh-Linc00707 #2+CQ
oe-NC+CQ
oe-Linc00707+CQ
①
②
③

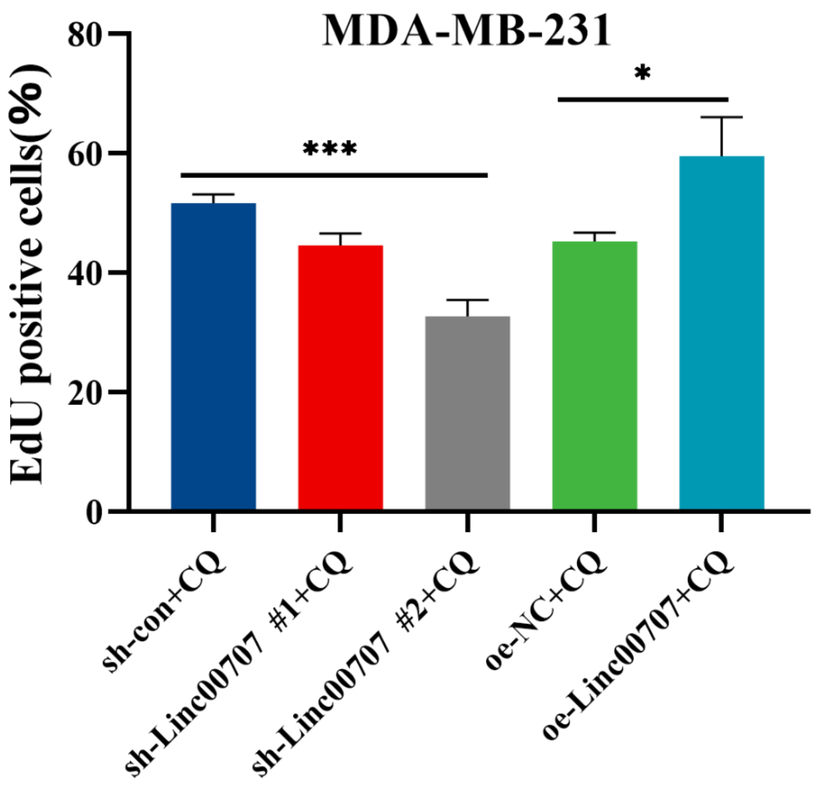
MDA-MB-231
EdU positive cells(%)
80
60
40
20
0
***
*
sh-con+CQ
sh-Linc00707 #1+CQ
sh-Linc00707 #2+CQ
oe-NC+CQ
oe-Linc00707+CQ

4B-468EDU

sh-con+CQ sh-Linc00707 #1+CQ sh-Linc00707 #2+CQ oe-NC+CQ oe-Linc00707+CQ

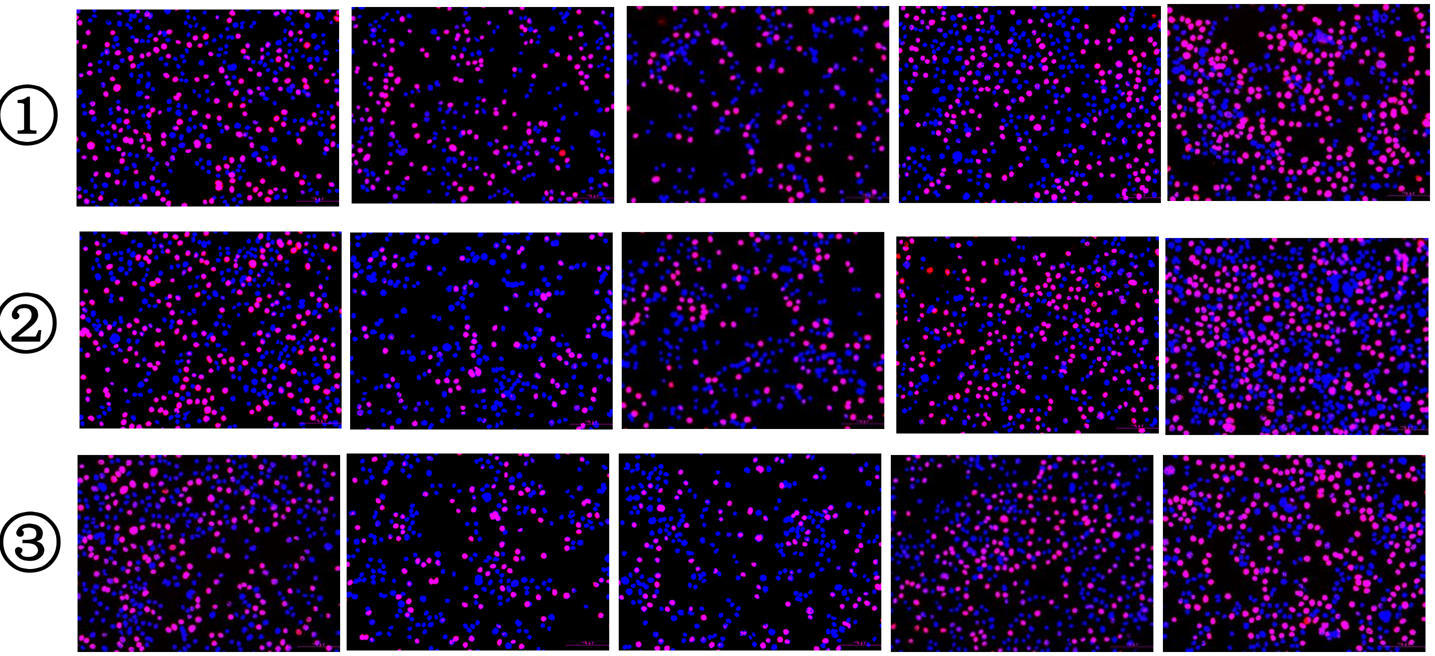

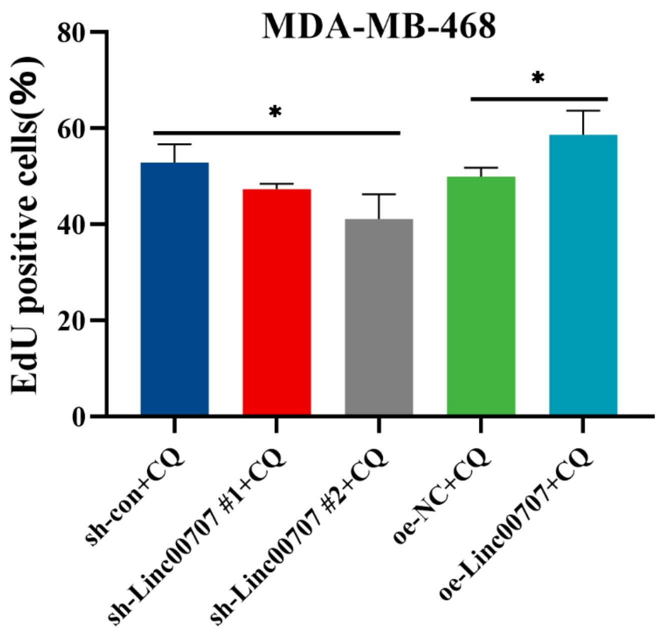

4C-231TRANSWELL

Migration

sh-con+CQ sh-Linc00707 #1+CQ sh-Linc00707 #2+CQ oe-NC+CQ oe-Linc00707+CQ

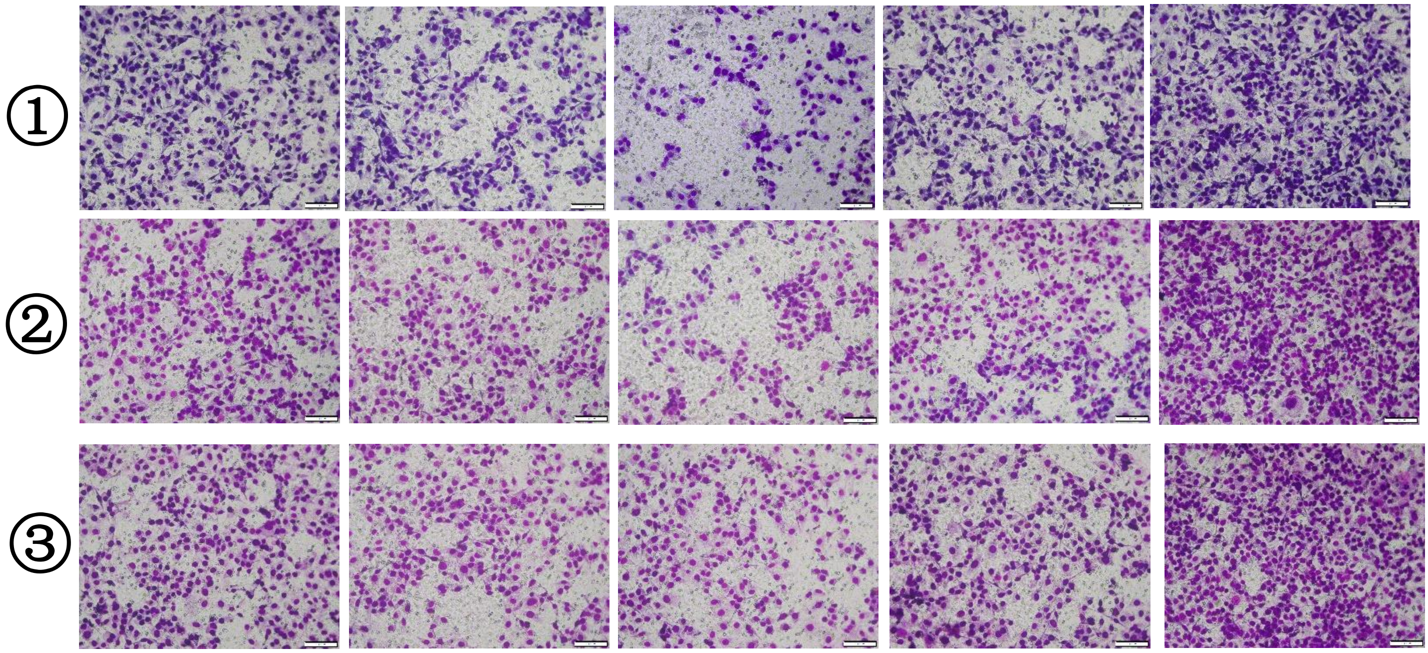

Invasion

sh-con+CQ sh-Linc00707 #1+CQ sh-Linc00707 #2+CQ oe-NC+CQ oe-Linc00707+CQ

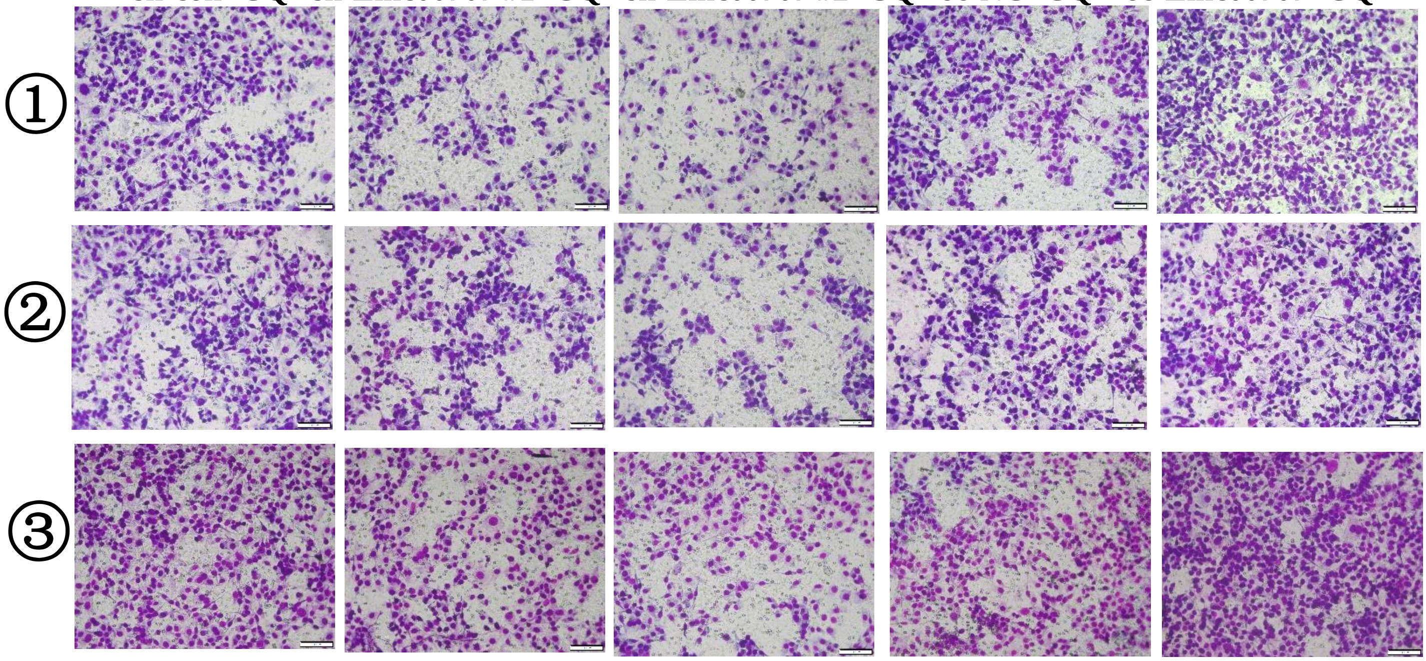

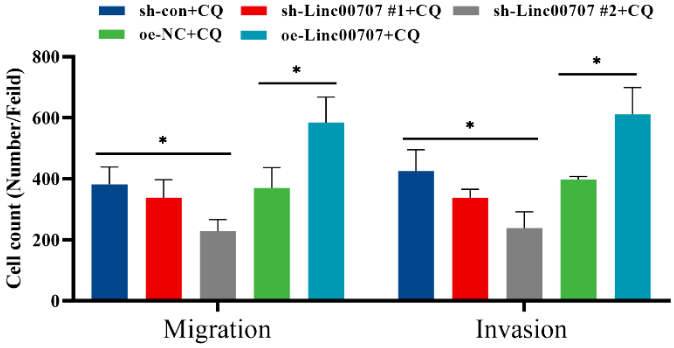

4D-468TRANSWELL

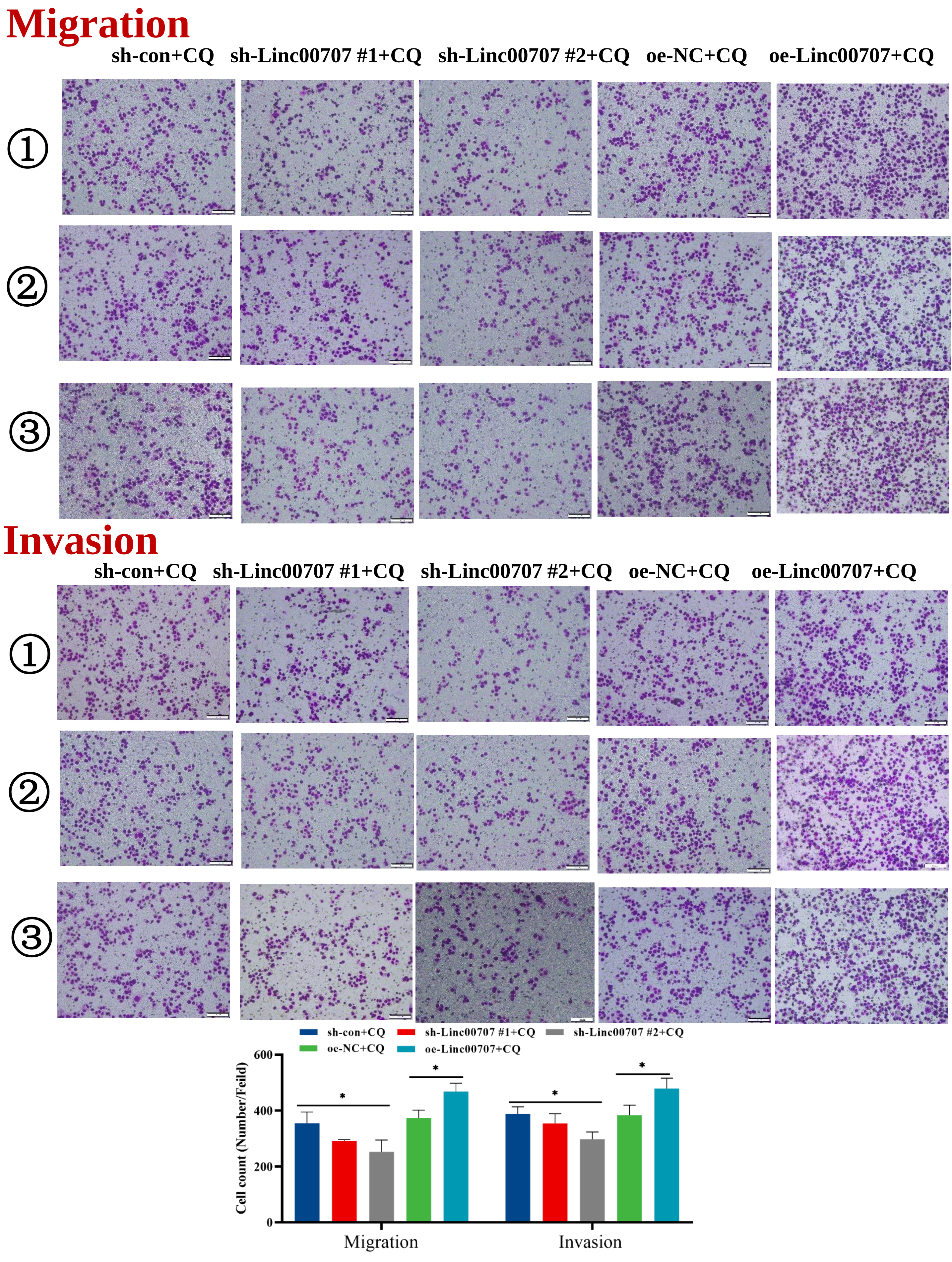

4E-231woundhealing

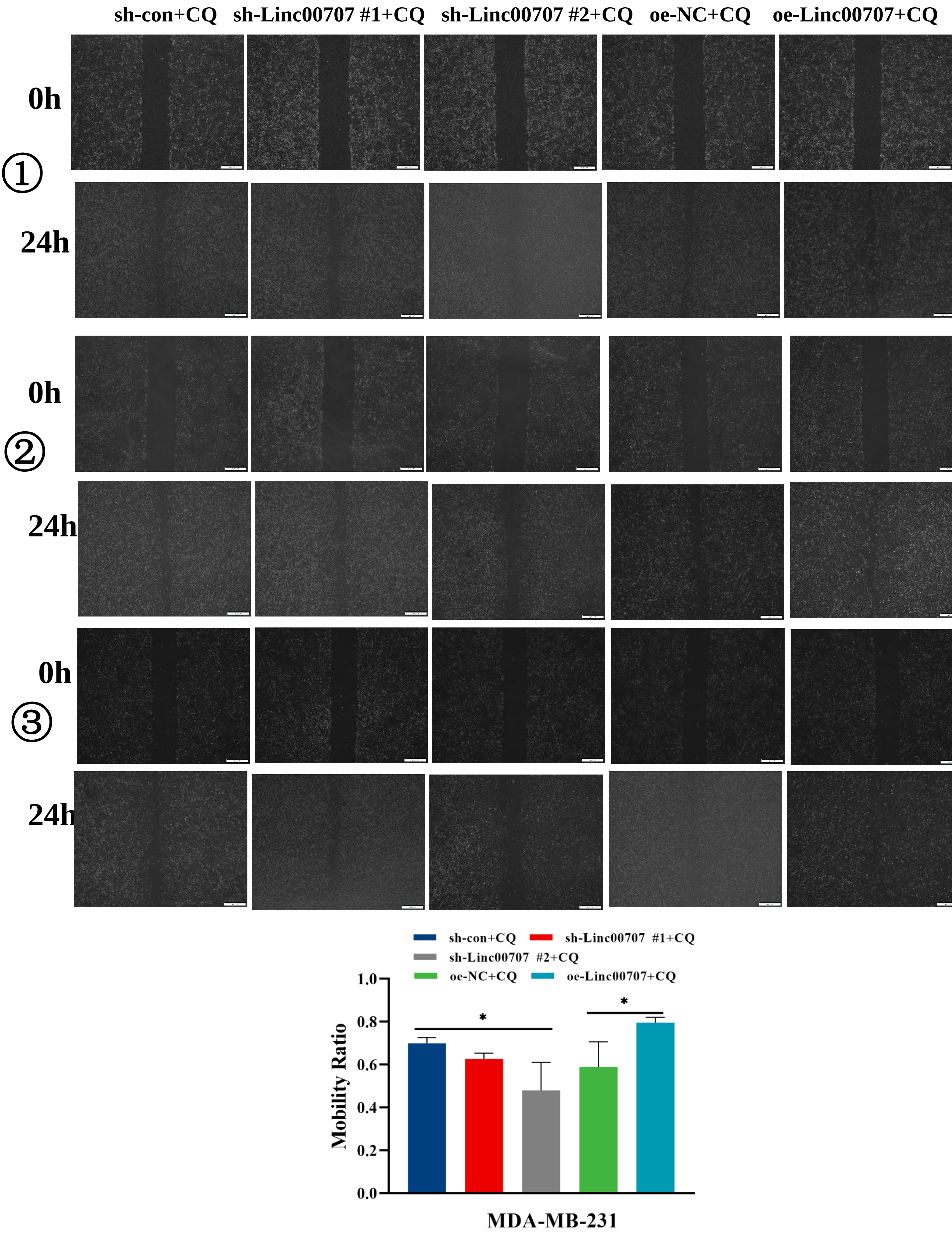

4E-468woundhealing

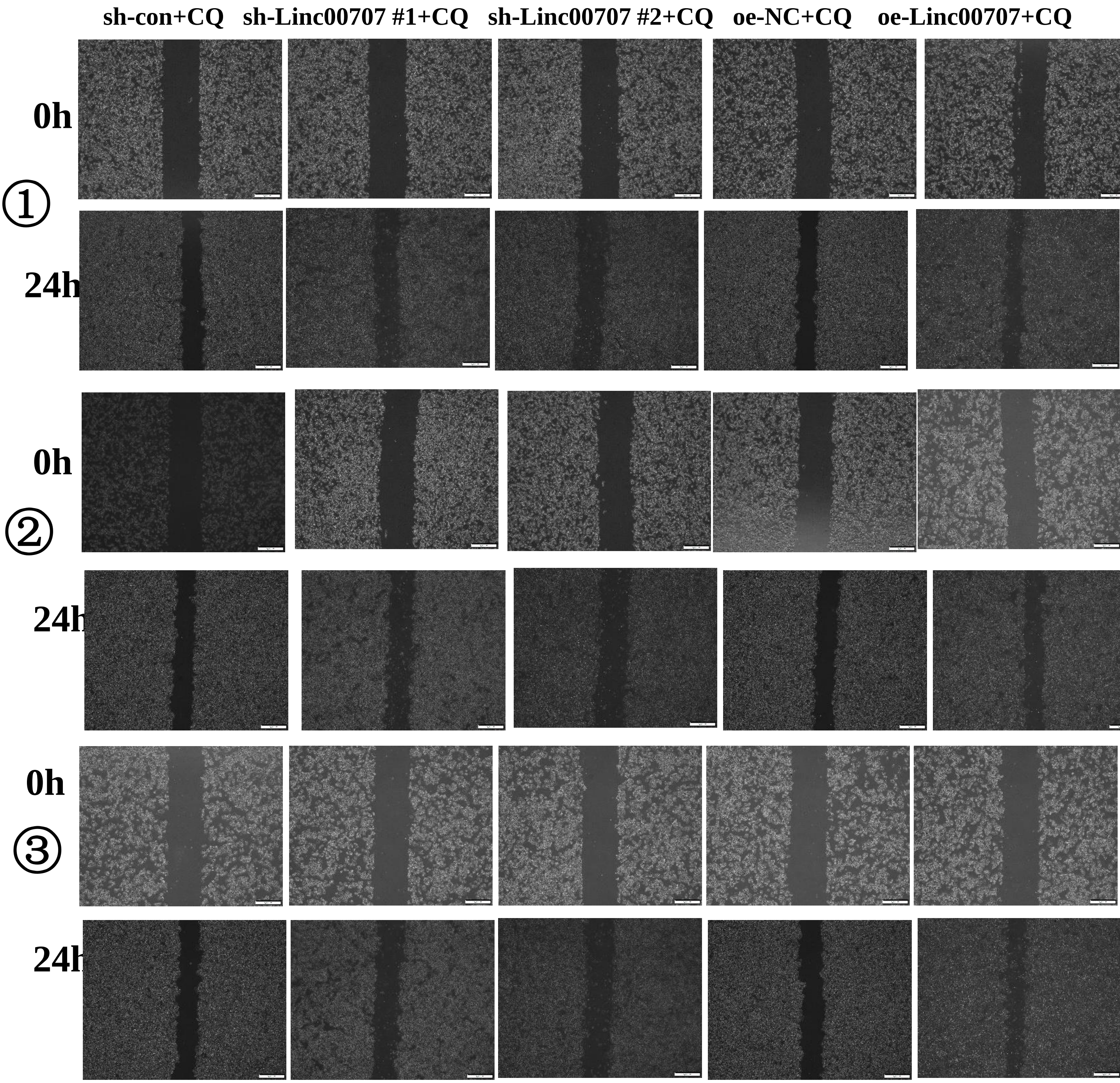

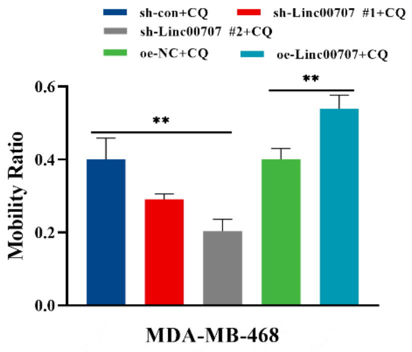

FIG6A-231EdU

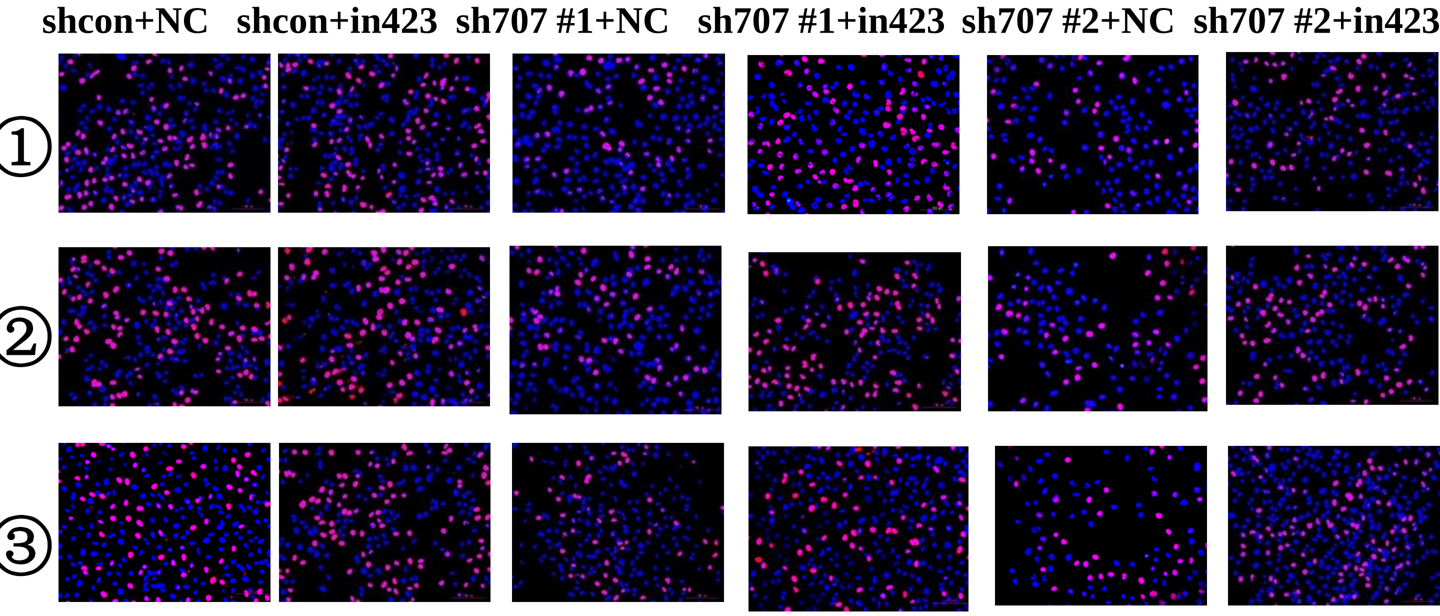

FIG6B-468EdU

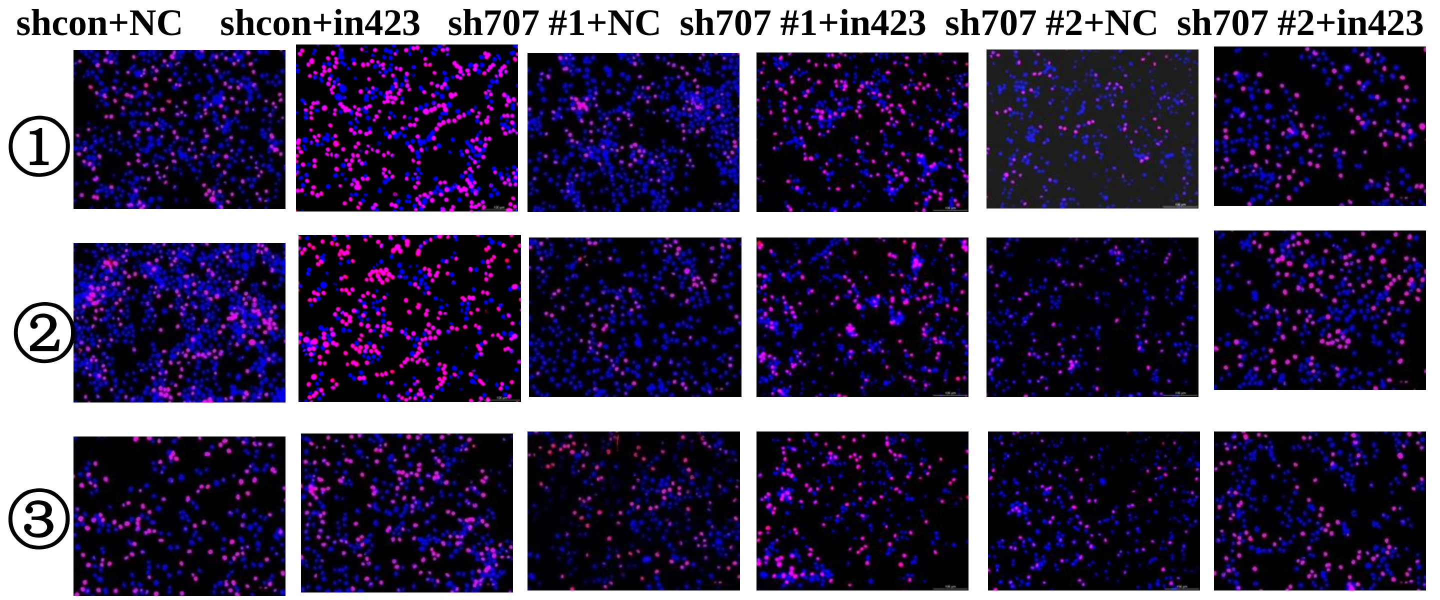

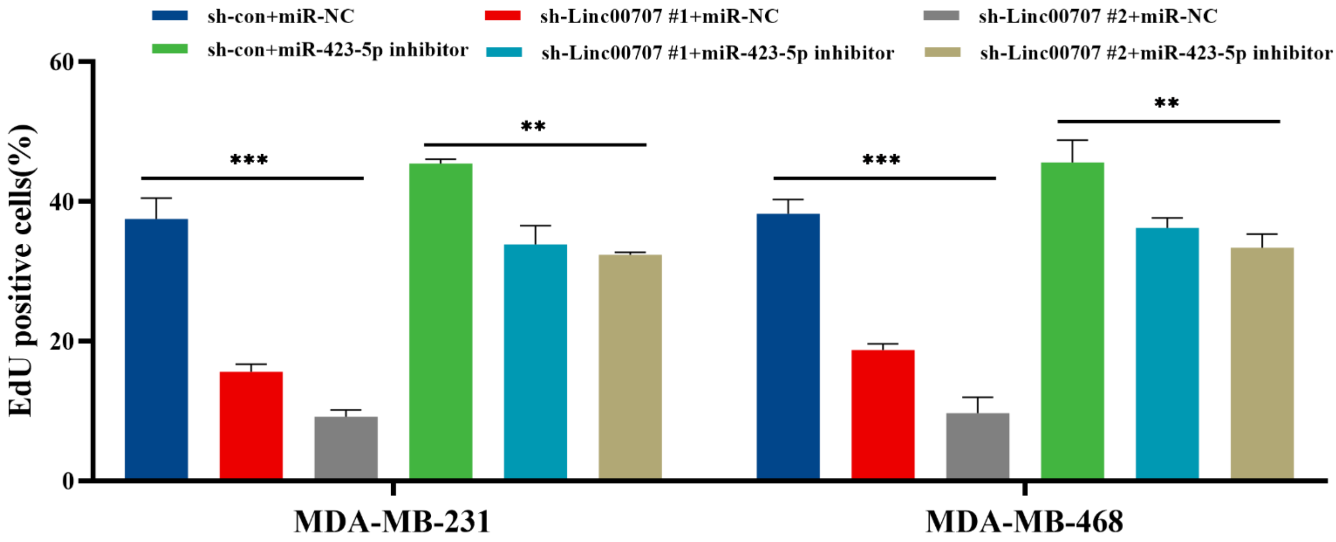

FIG6D-231TRANSWELL

Invasion

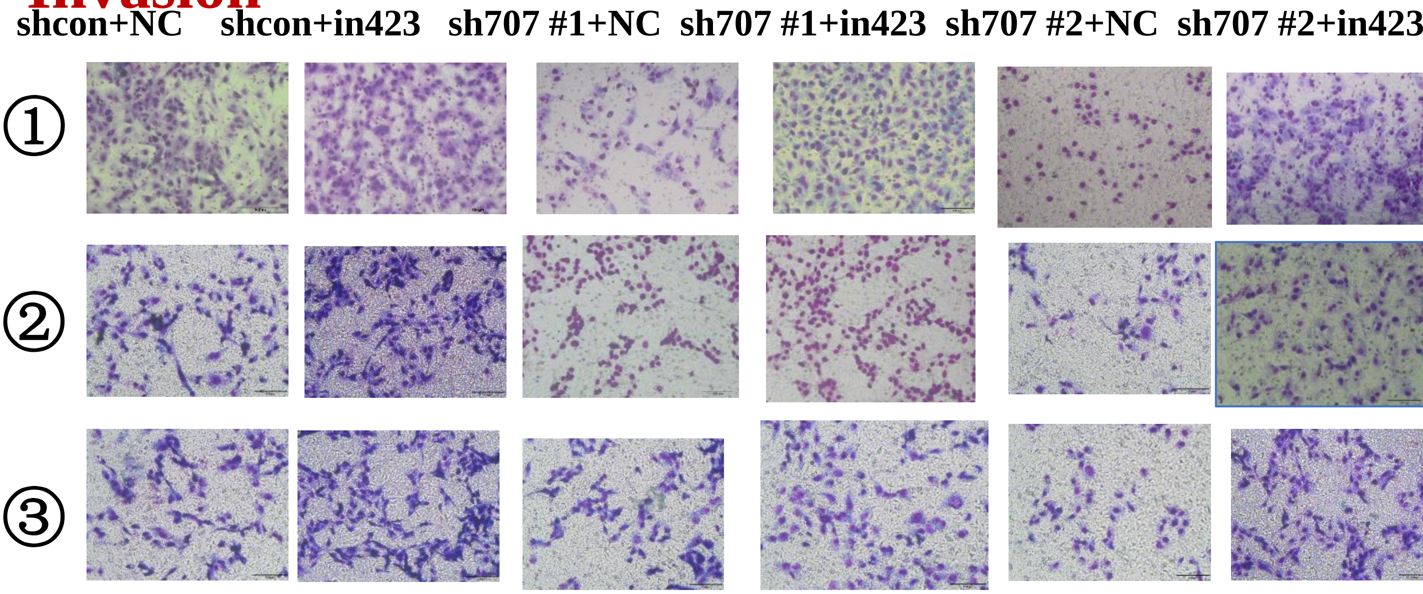

Migration

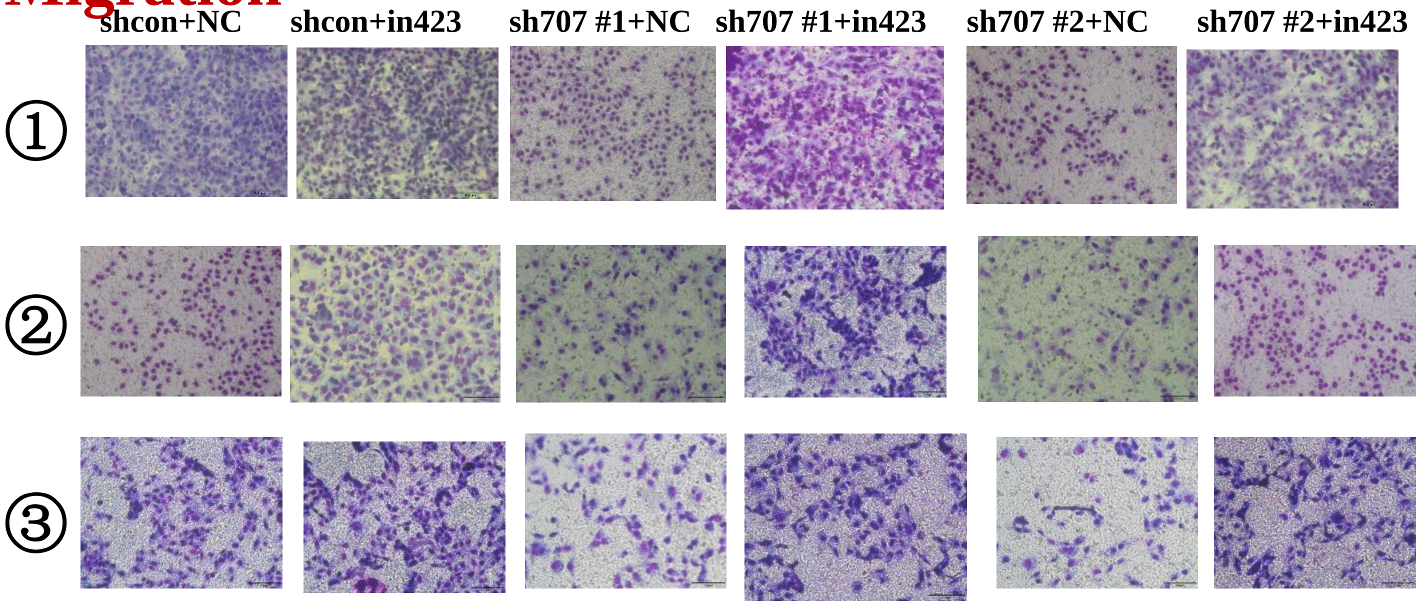

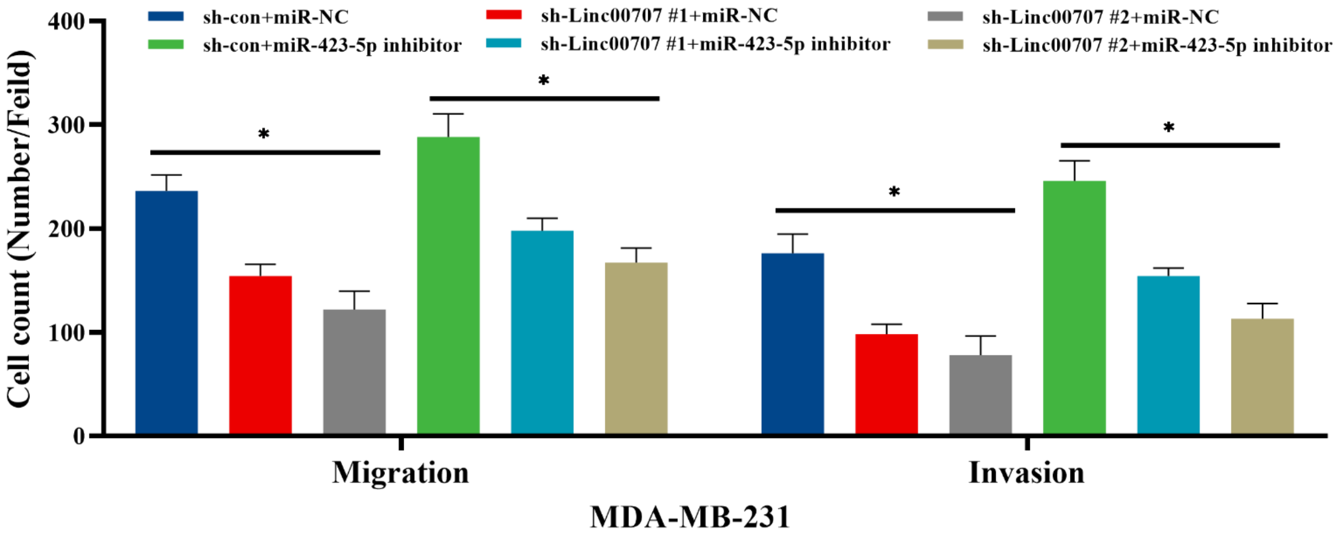

FIG6G-468TRANSWELL

Invasion

shcon+NC shcon+in423 sh707 #1+NC sh707 #1+in423 sh707 #2+NC sh707 #2+in423

① ② ③

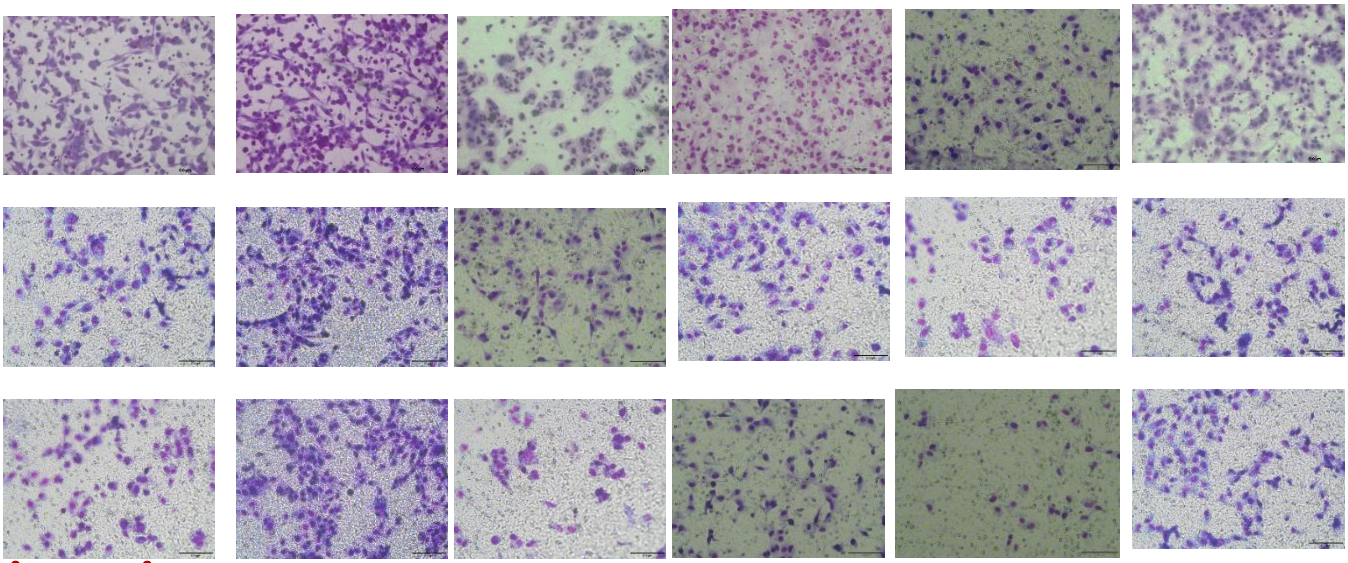

Migration

shcon+NC shcon+in423 sh707 #1+NC sh707 #1+in423 sh707 #2+NC sh707 #2+in423

① ② ③

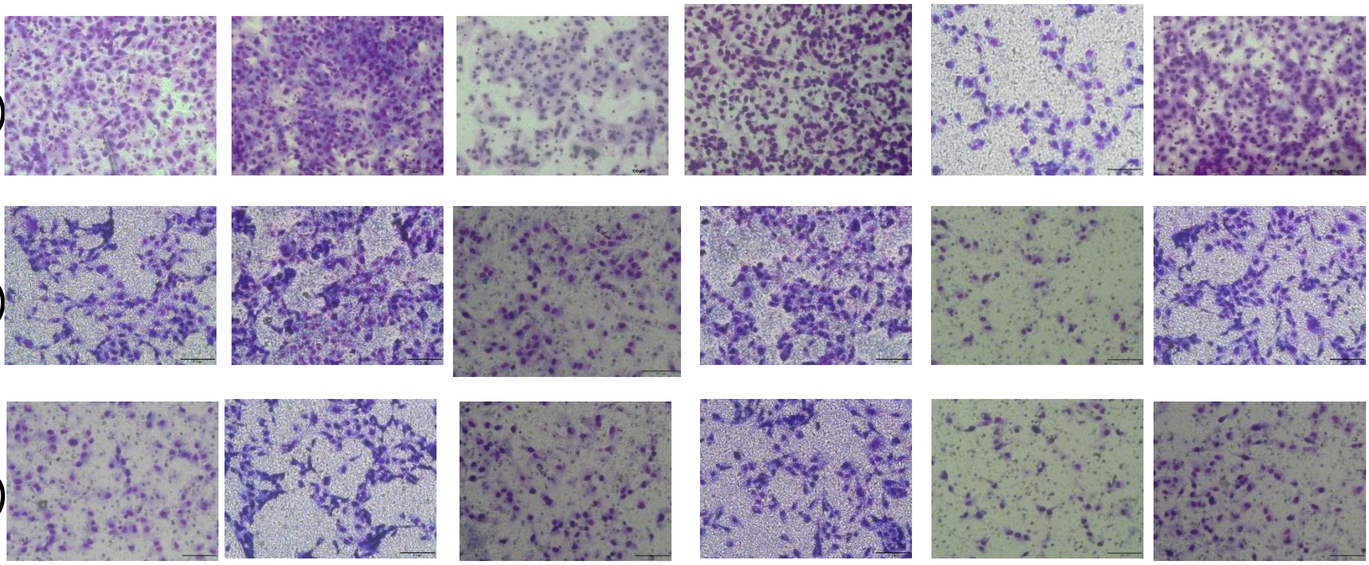

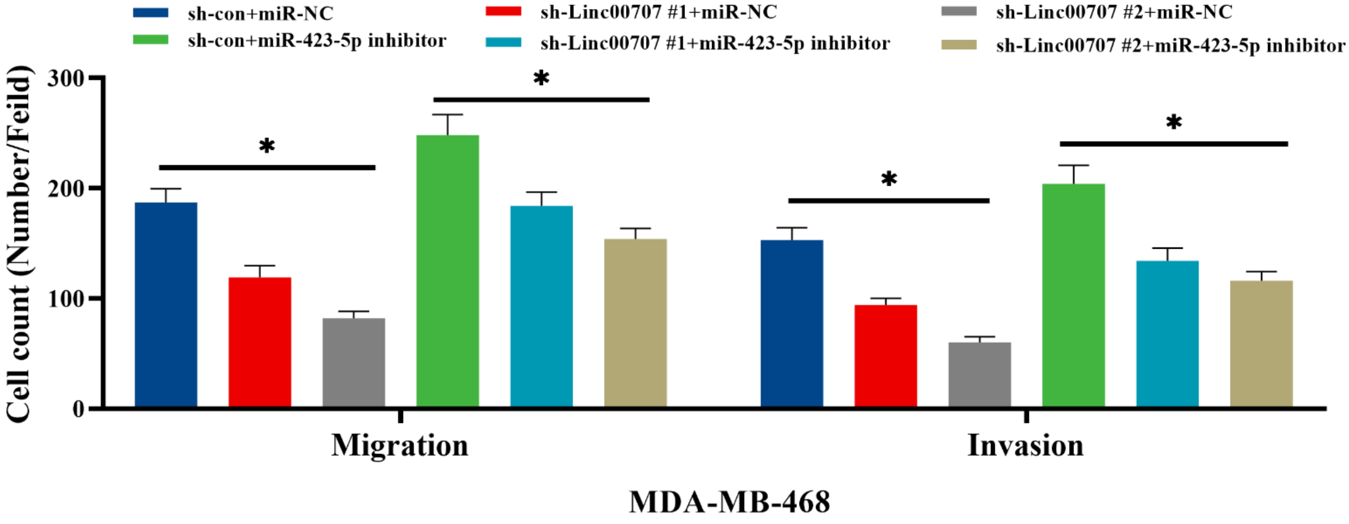

FIG7C

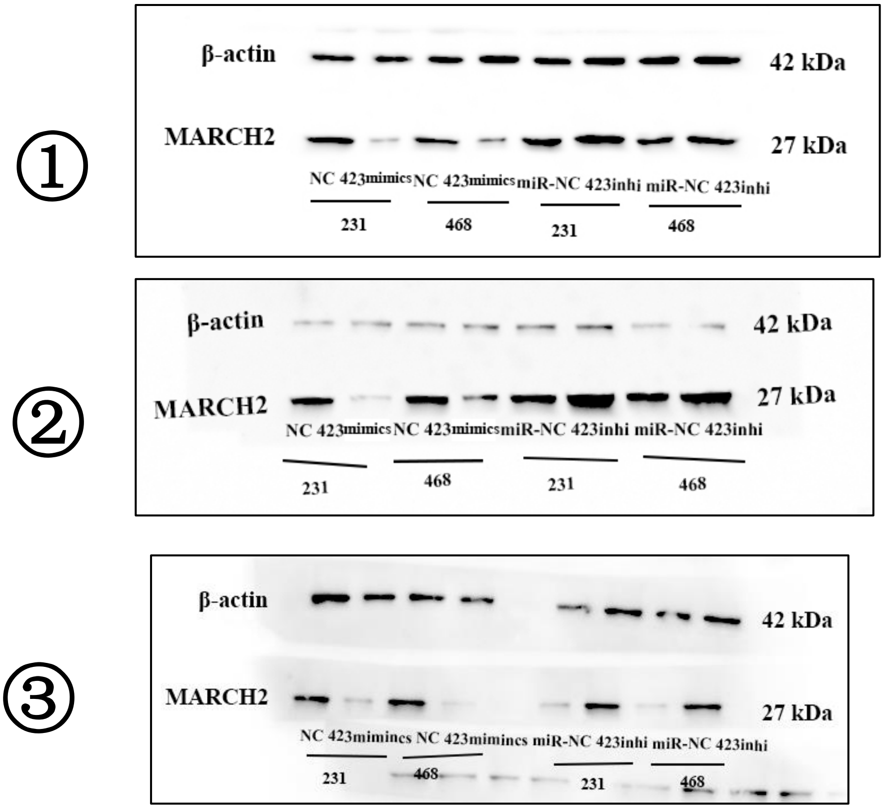
①
β-actin
42 kDa
MARCH2
27 kDa
NC 423mimics NC 423mimics miR-NC 423inhi miR-NC 423inhi
231
468
231
468
②
β-actin
42 kDa
MARCH2
27 kDa
NC 423mimics NC 423mimics miR-NC 423inhi miR-NC 423inhi
231
468
231
468
③
β-actin
42 kDa
MARCH2
27 kDa
NC 423mimincs NC 423mimincs miR-NC 423inhi miR-NC 423inhi
231
468
231
468

FIG7D

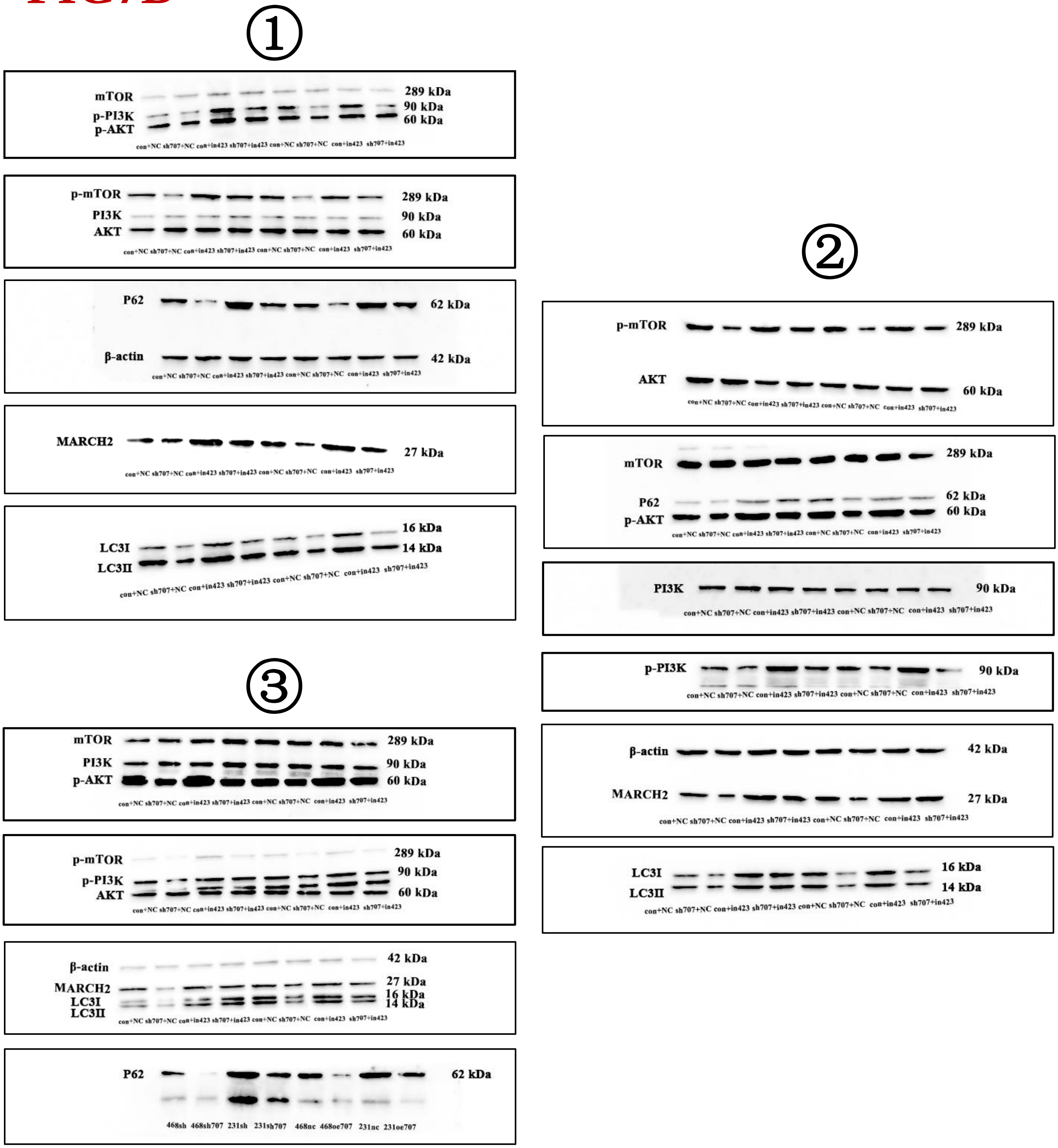

①
mTOR
p-PI3K
p-AKT
289 kDa
90 kDa
60 kDa
con+NC sh707+NC con+in423 sh707+in423 con+NC sh707+NC con+in423 sh707+in423
p-mTOR
PI3K
AKT
289 kDa
90 kDa
60 kDa
P62
62 kDa
β-actin
42 kDa
MARCH2
27 kDa
LC3I
LC3II
16 kDa
14 kDa
②
p-mTOR
289 kDa
AKT
60 kDa
mTOR
289 kDa
P62
p-AKT
62 kDa
60 kDa
PI3K
90 kDa
p-PI3K
90 kDa
β-actin
42 kDa
MARCH2
27 kDa
LC3I
LC3II
16 kDa
14 kDa
③
mTOR
PI3K
p-AKT
289 kDa
90 kDa
60 kDa
p-mTOR
p-PI3K
AKT
289 kDa
90 kDa
60 kDa
β-actin
MARCH2
LC3I
LC3II
42 kDa
27 kDa
16 kDa
14 kDa
P62
62 kDa
468sh 468sh707 231sh 231sh707 468nc 468oe707 231nc 231oe707

SUP1A-231克隆形成

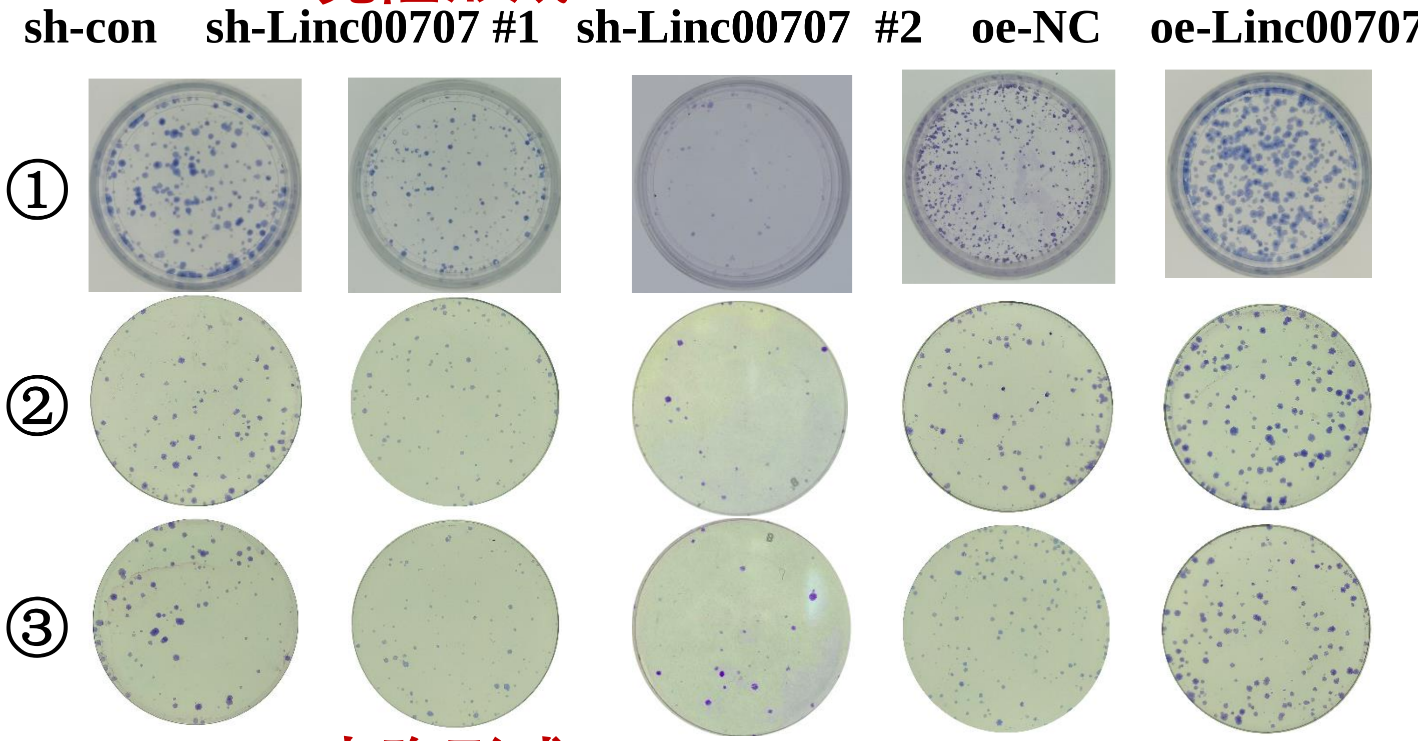

SUP1A-468克隆形成

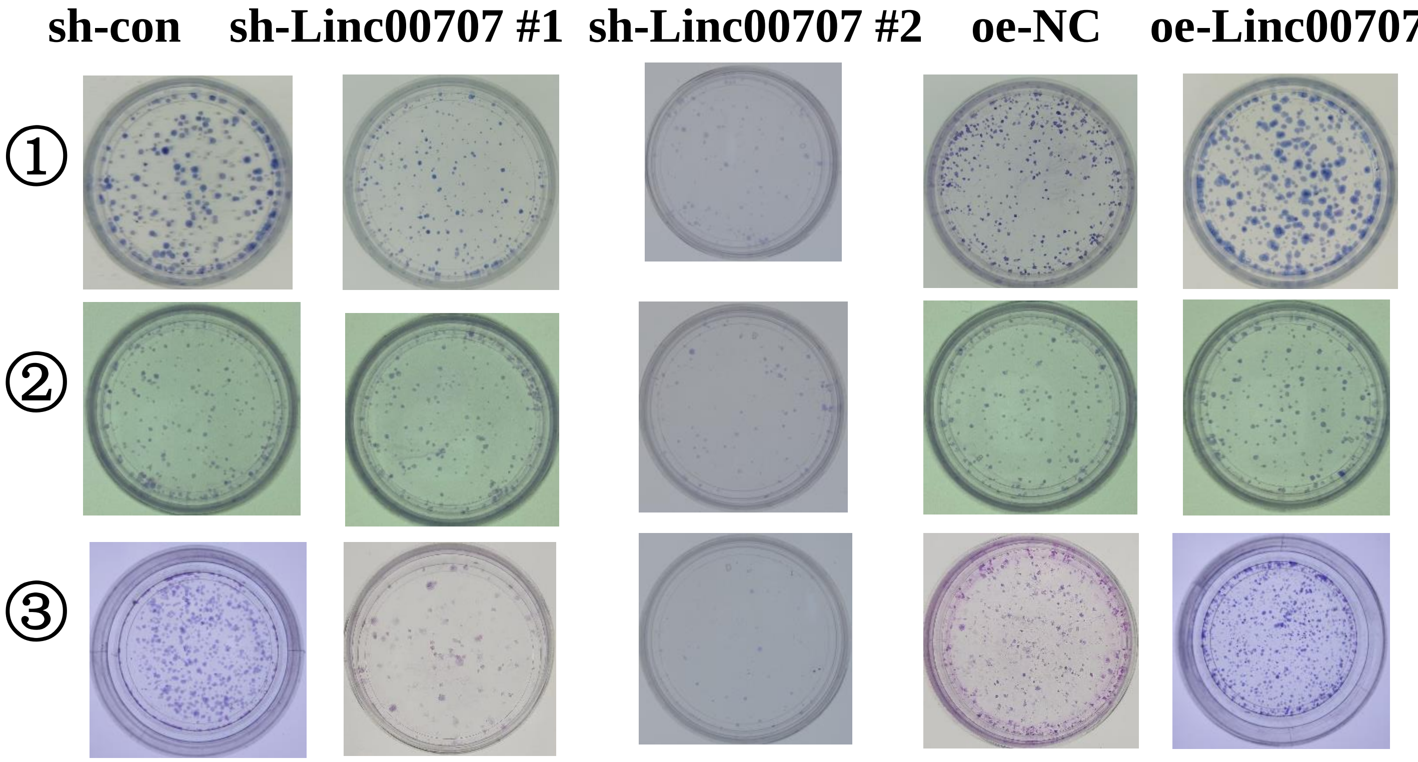

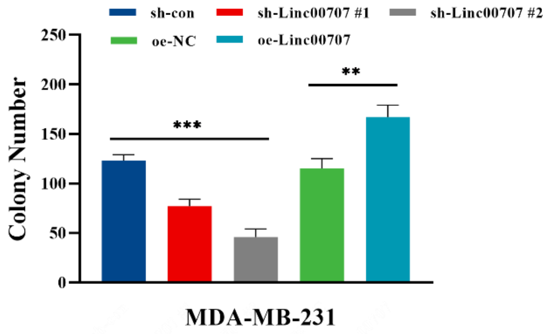

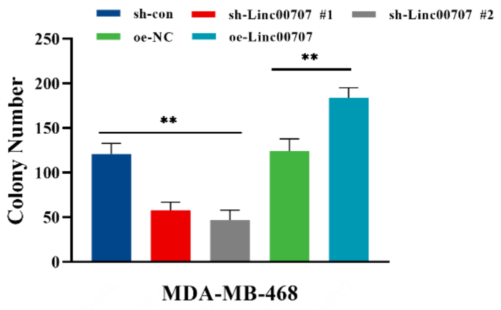

SUP1C-231woundhealing

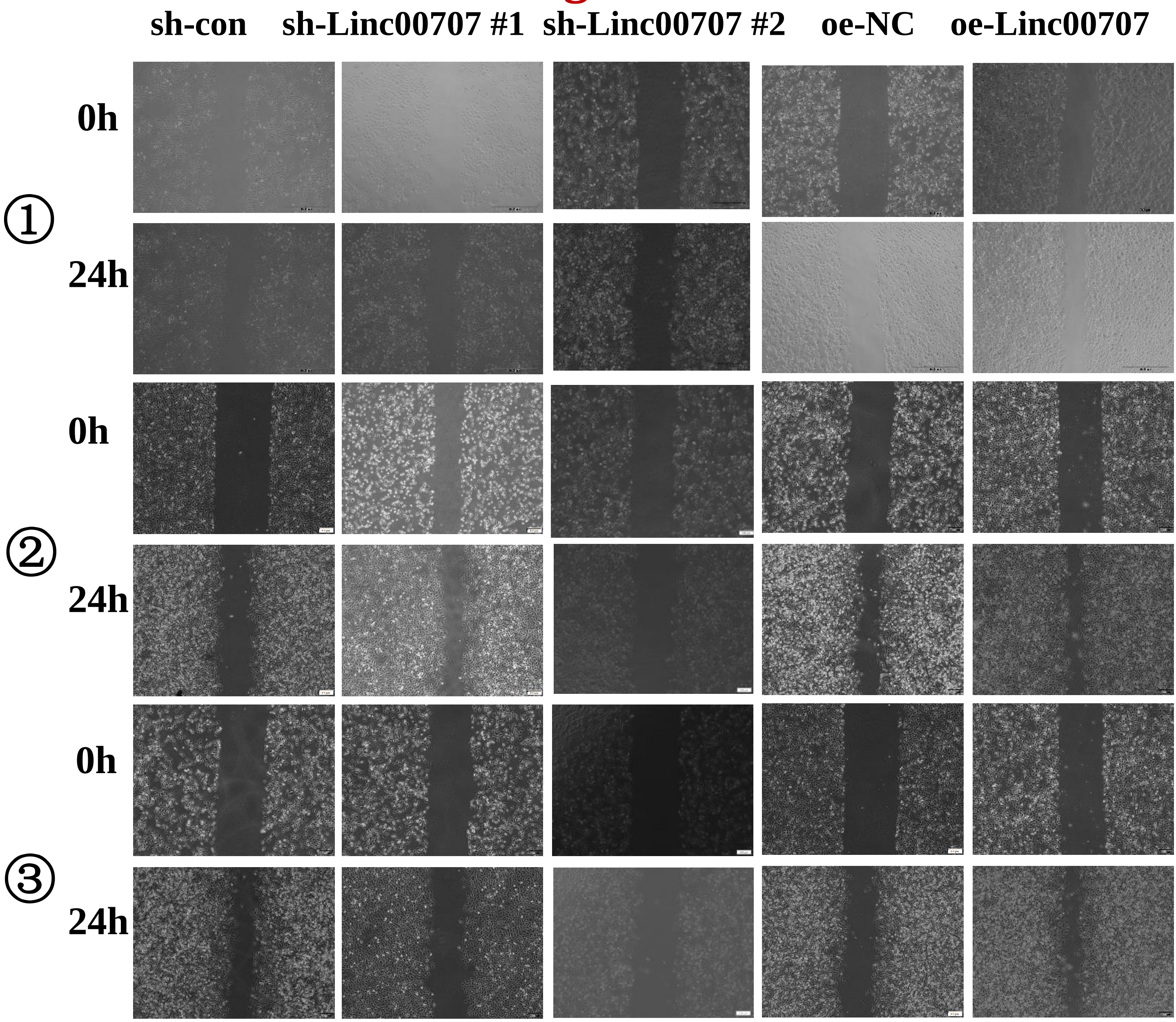

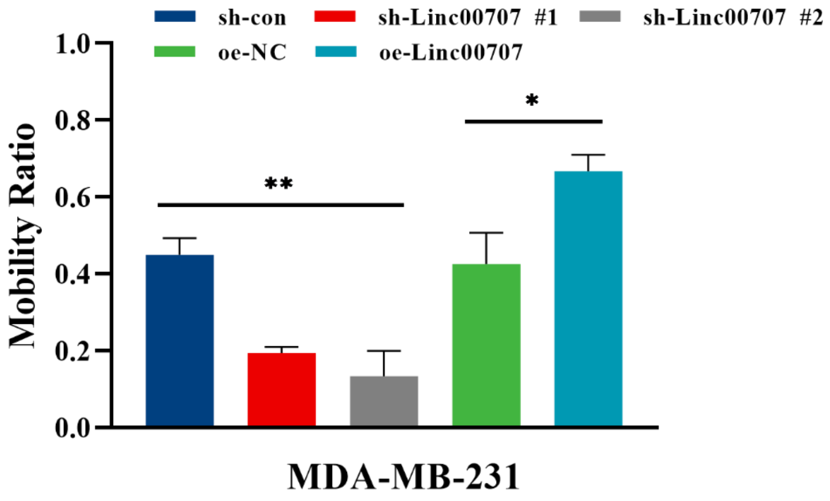

SUP1D-468woundhealing

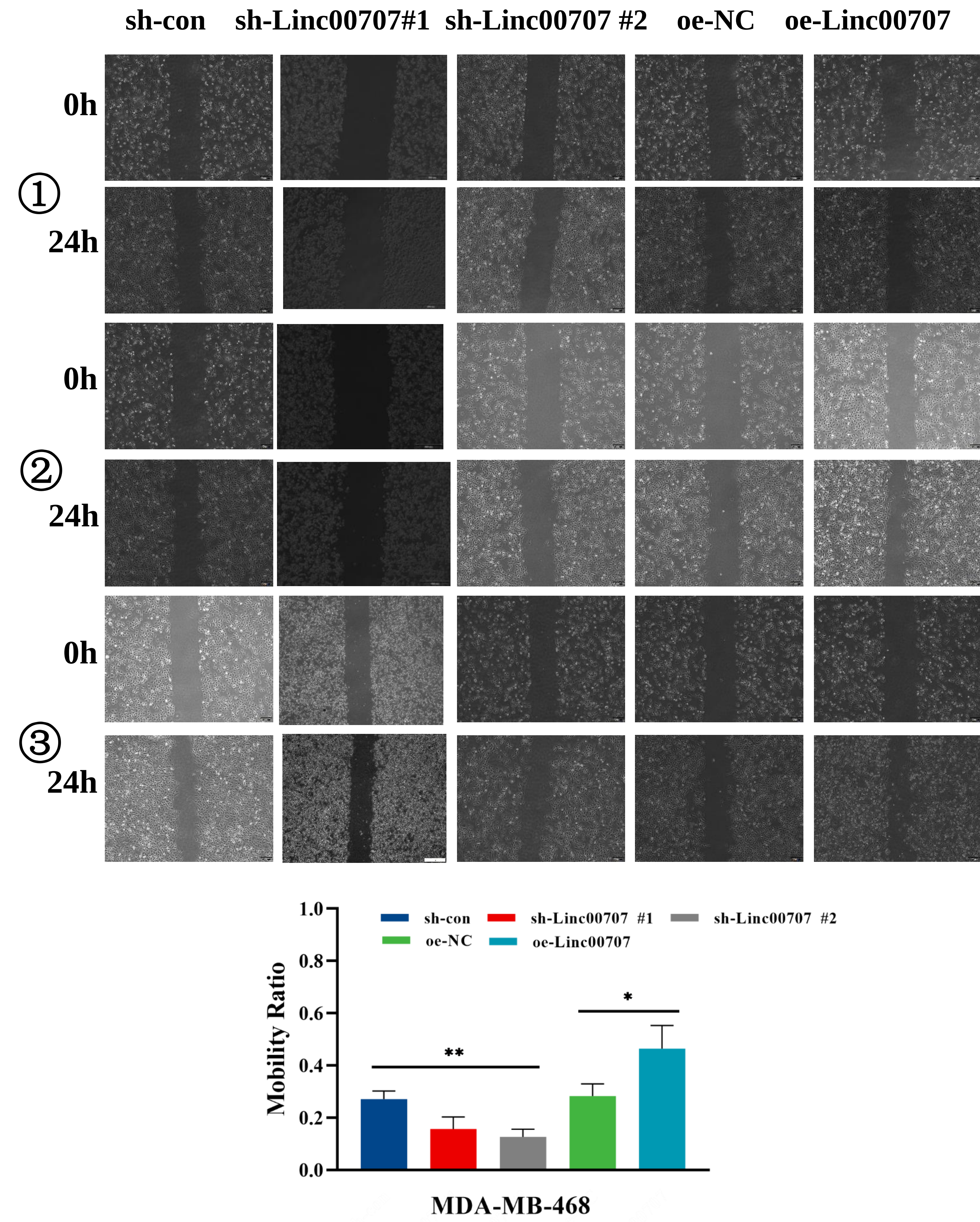

SUPD2A

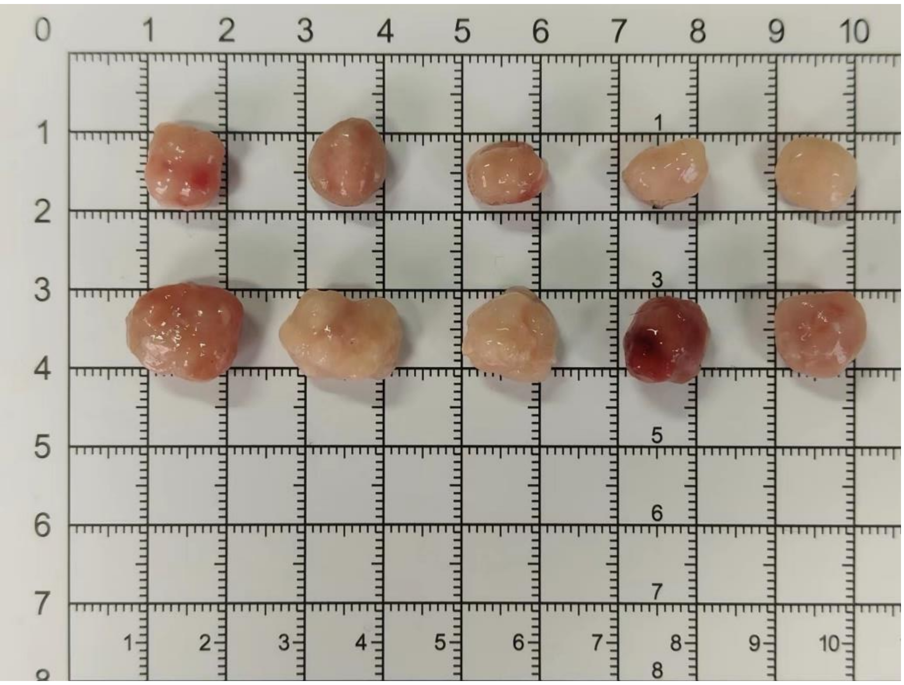
0
1
2
3
4
5
6
7
8
9
10

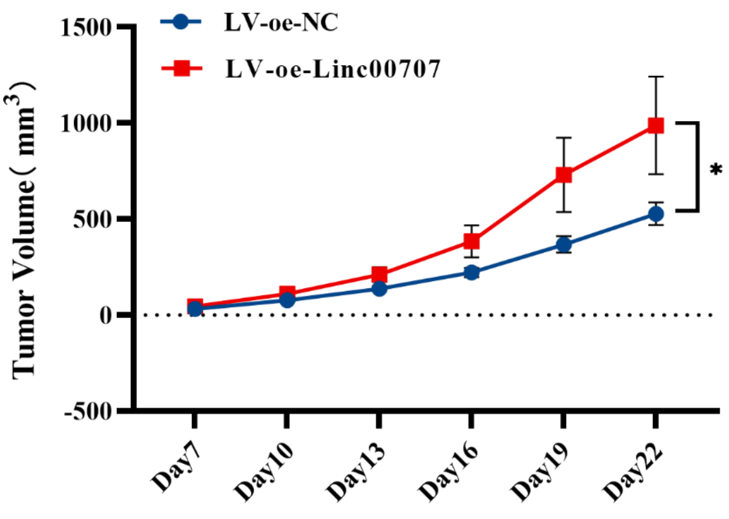
LV-oe-NC
LV-oe-Linc00707
Tumor Volume（mm^3）
1500
1000
500
0
-500
*
Day7
Day10
Day13
Day16
Day19
Day22

SUPD2B

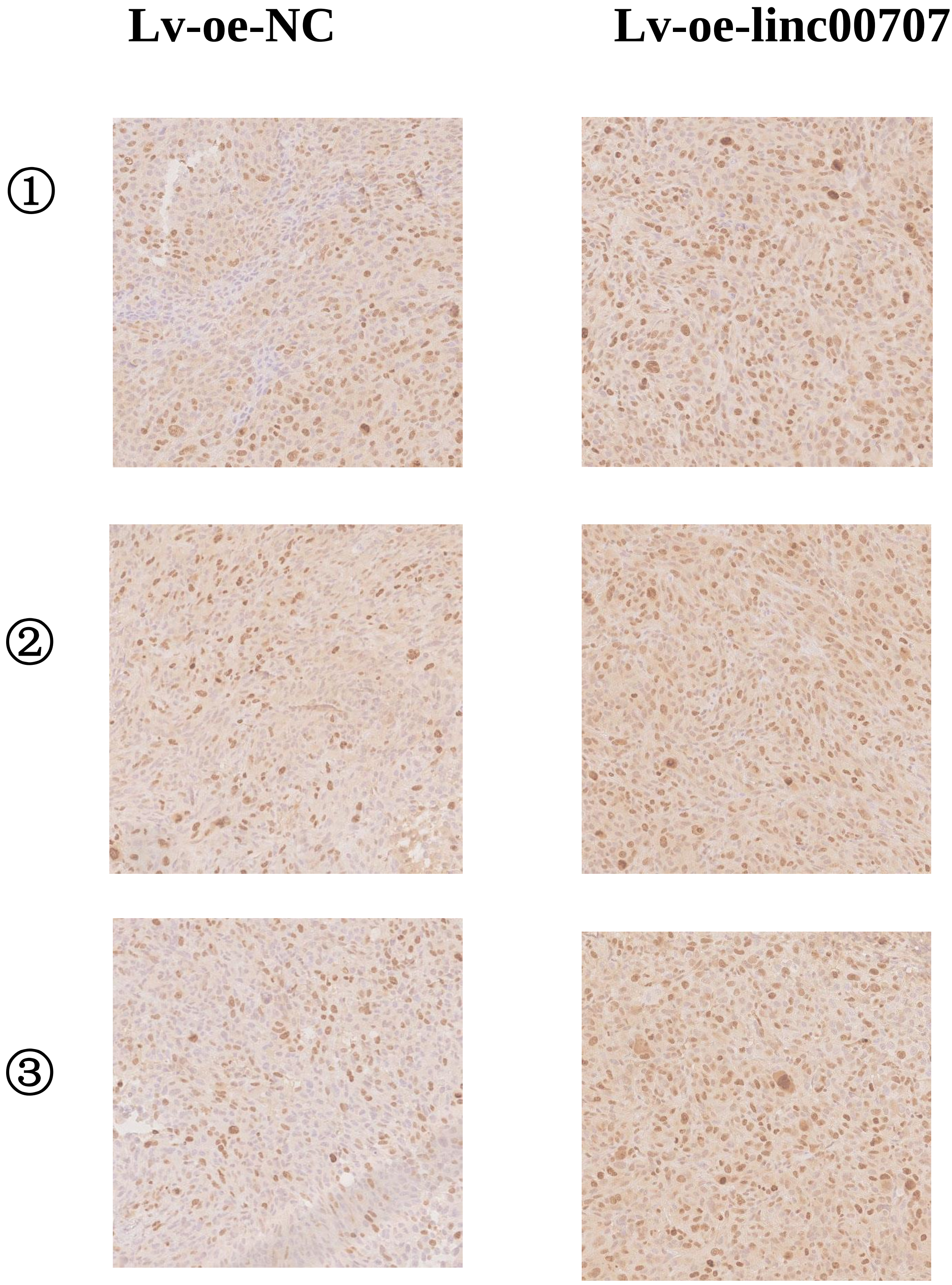

SUPD2C

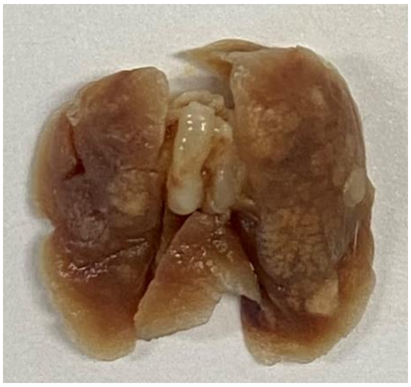

Lv-oe-NC

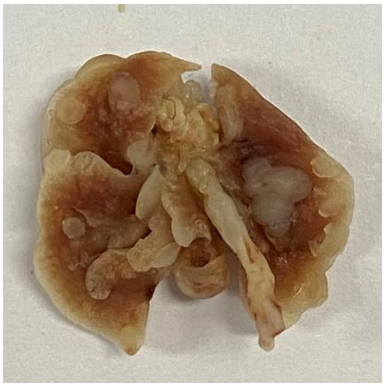

Lv-oe-linc00707

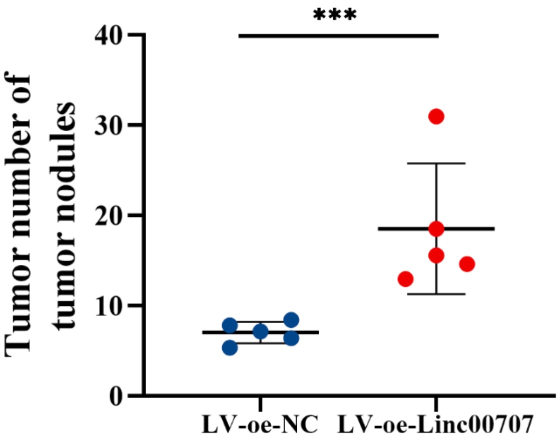

SUPD2D

Lv-oe-NC

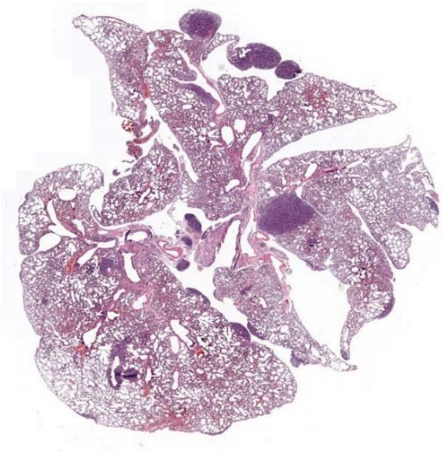

Lv-oe-linc00707

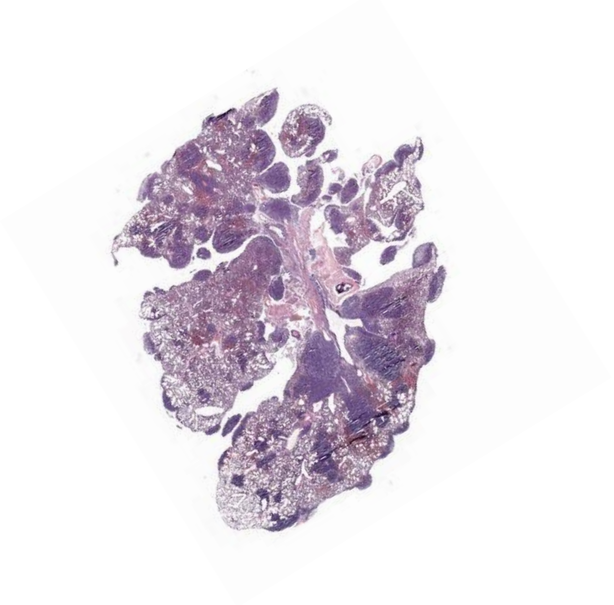

FIG4A-LC3

231

	GFP	RFP	Merge
① sh-con+LY294002			
sh-Linc00707 +LY294002			
② sh-con +LY294002			
sh-Linc00707 +LY294002			
③ sh-con +LY294002			
sh-Linc00707 +LY294002			

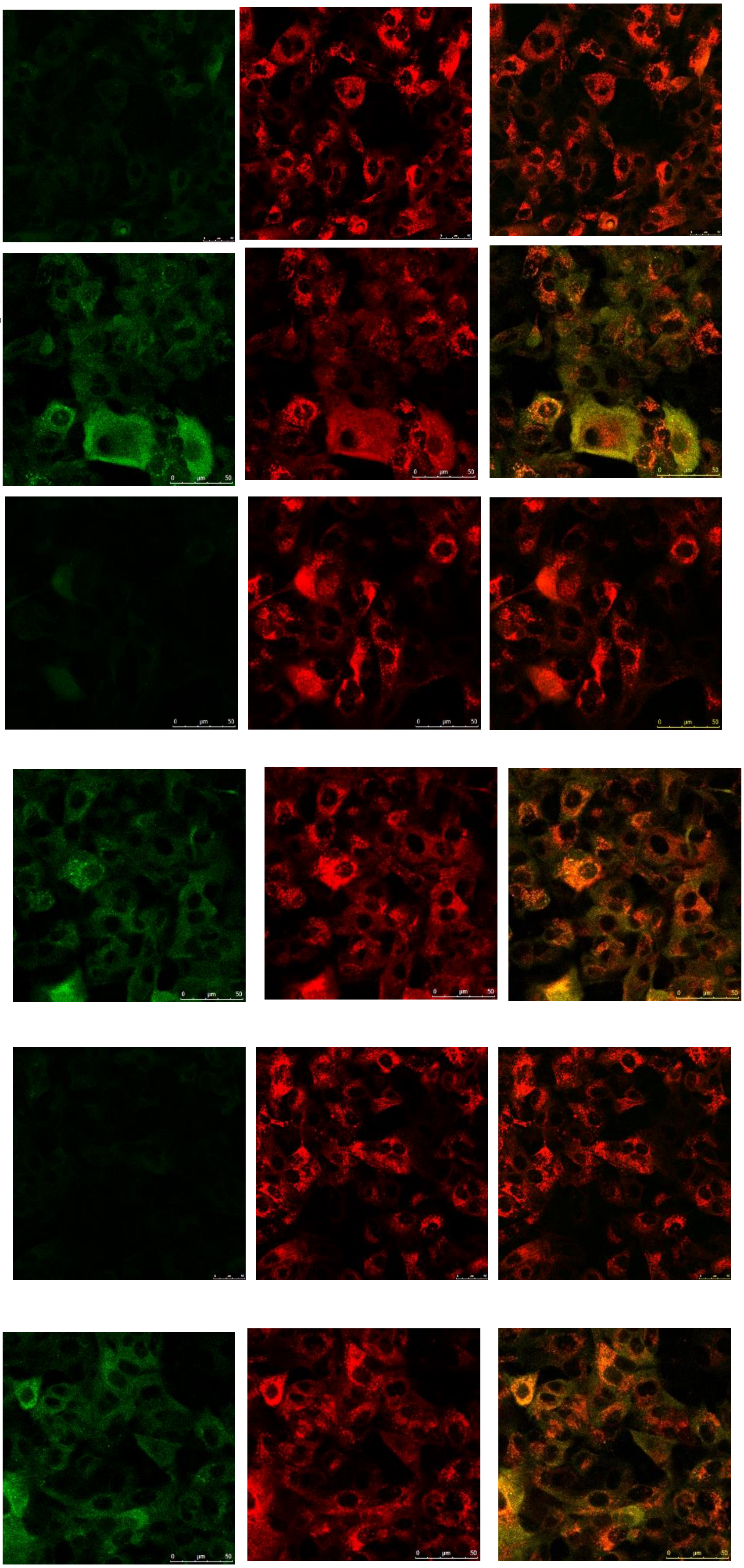

FIG4A-LC3

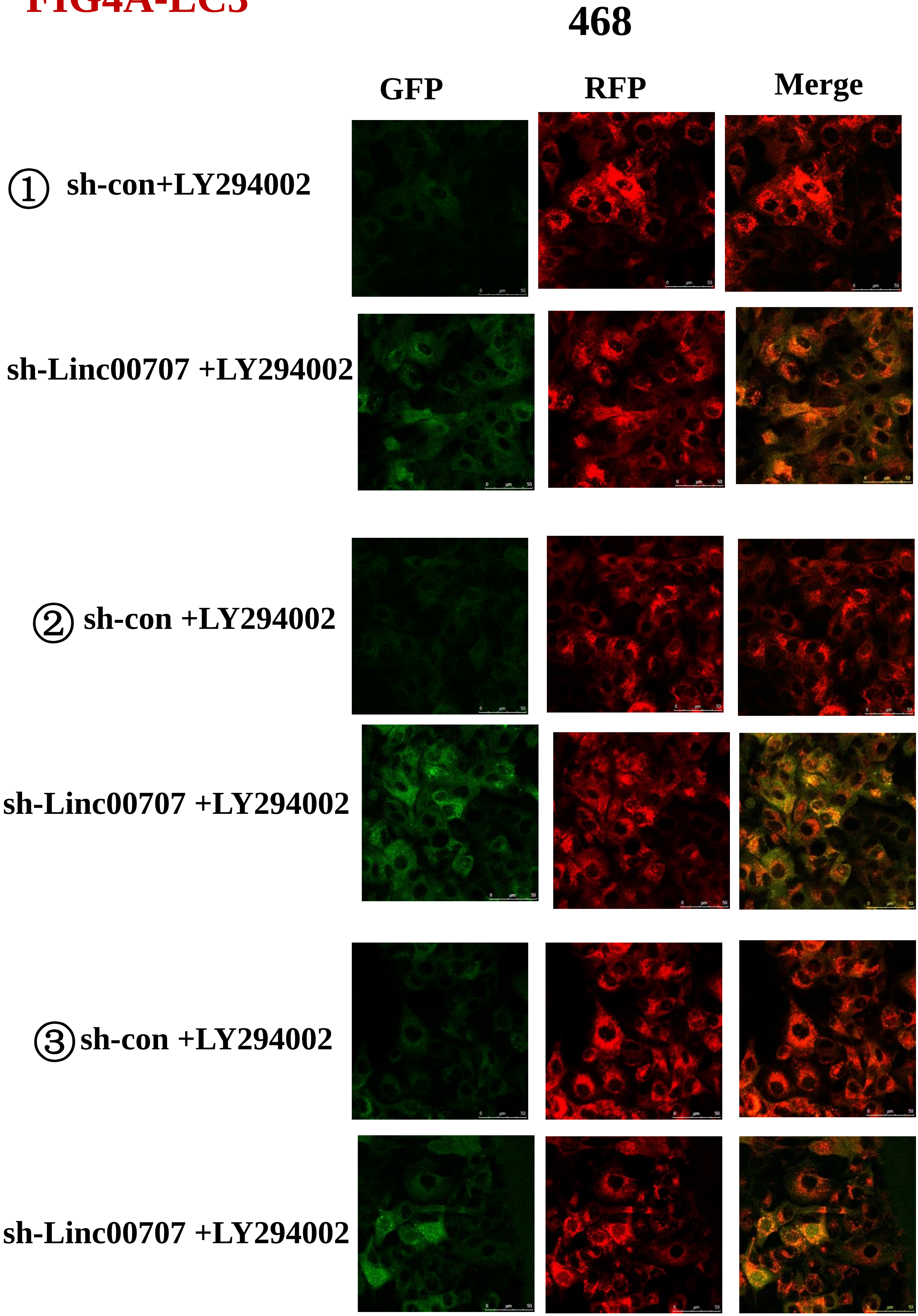
468
GFP
RFP
Merge
① sh-con+LY294002
sh-Linc00707 +LY294002
② sh-con +LY294002
sh-Linc00707 +LY294002
③ sh-con +LY294002
sh-Linc00707 +LY294002

FIG4B-JC-1

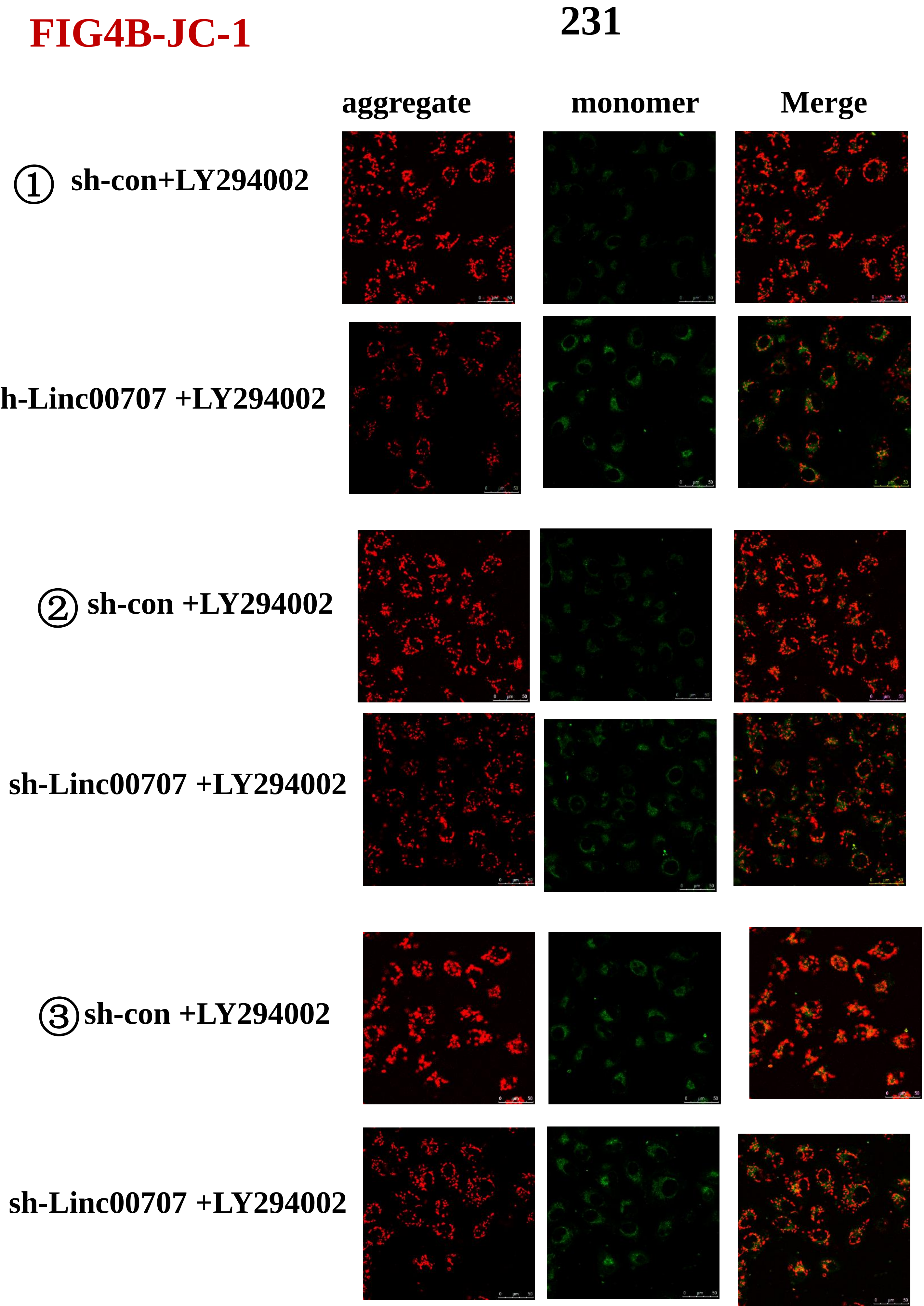

231
aggregate
monomer
Merge
① sh-con+LY294002
h-Linc00707 +LY294002
② sh-con +LY294002
sh-Linc00707 +LY294002
③ sh-con +LY294002
sh-Linc00707 +LY294002

FIG4B-JC-1

468

aggregate | monomer | Merge

① sh-con+LY294002

sh-Linc00707 +LY294002

② sh-con +LY294002

sh-Linc00707 +LY294002

③ sh-con +LY294002

sh-Linc00707 +LY294002

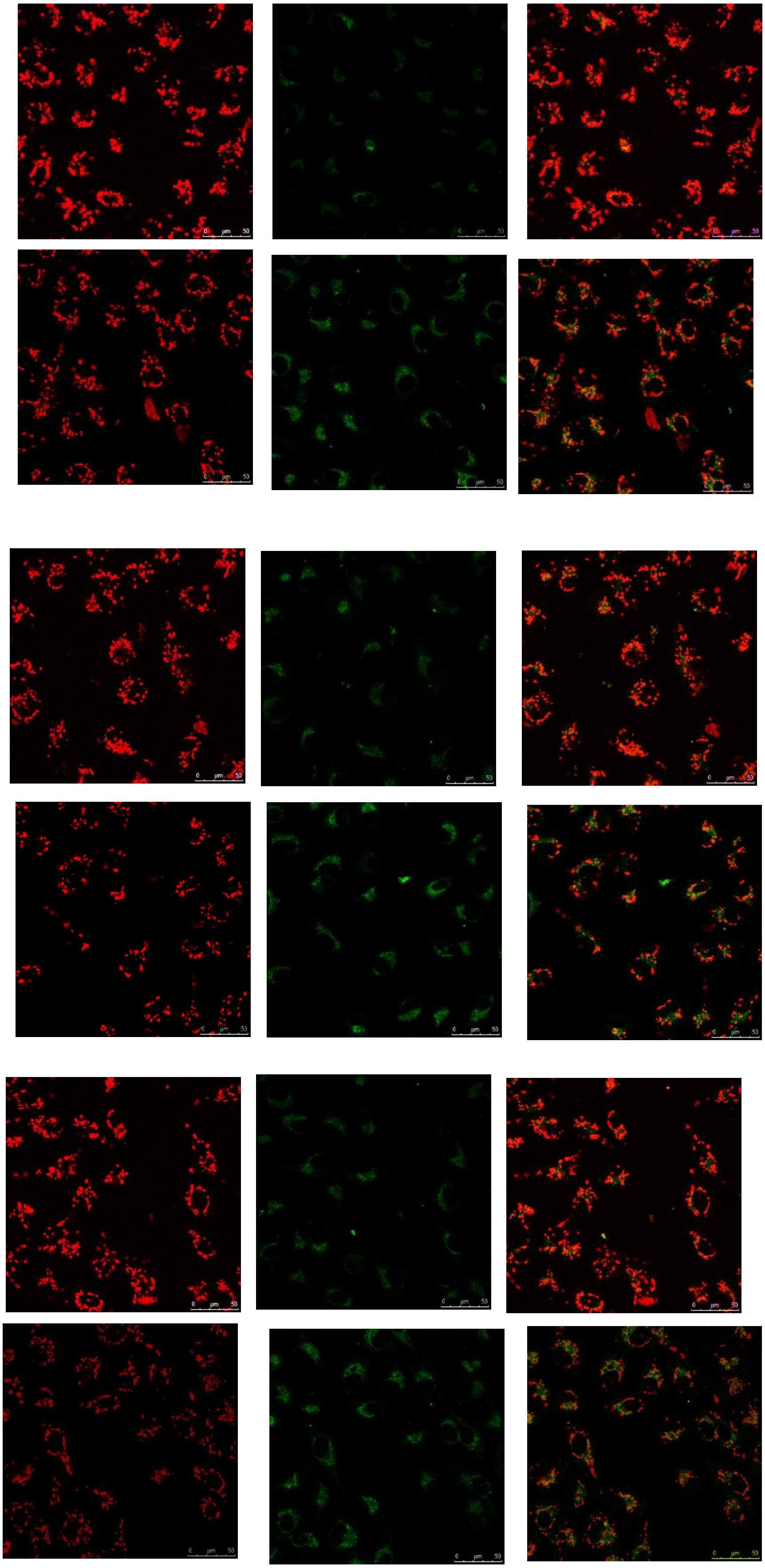

SUPD5A-231克隆形成

shcon+NC shcon+in423 sh707 #1+NC sh707 #1+in423 sh707 #2+NC sh707 #2+in423

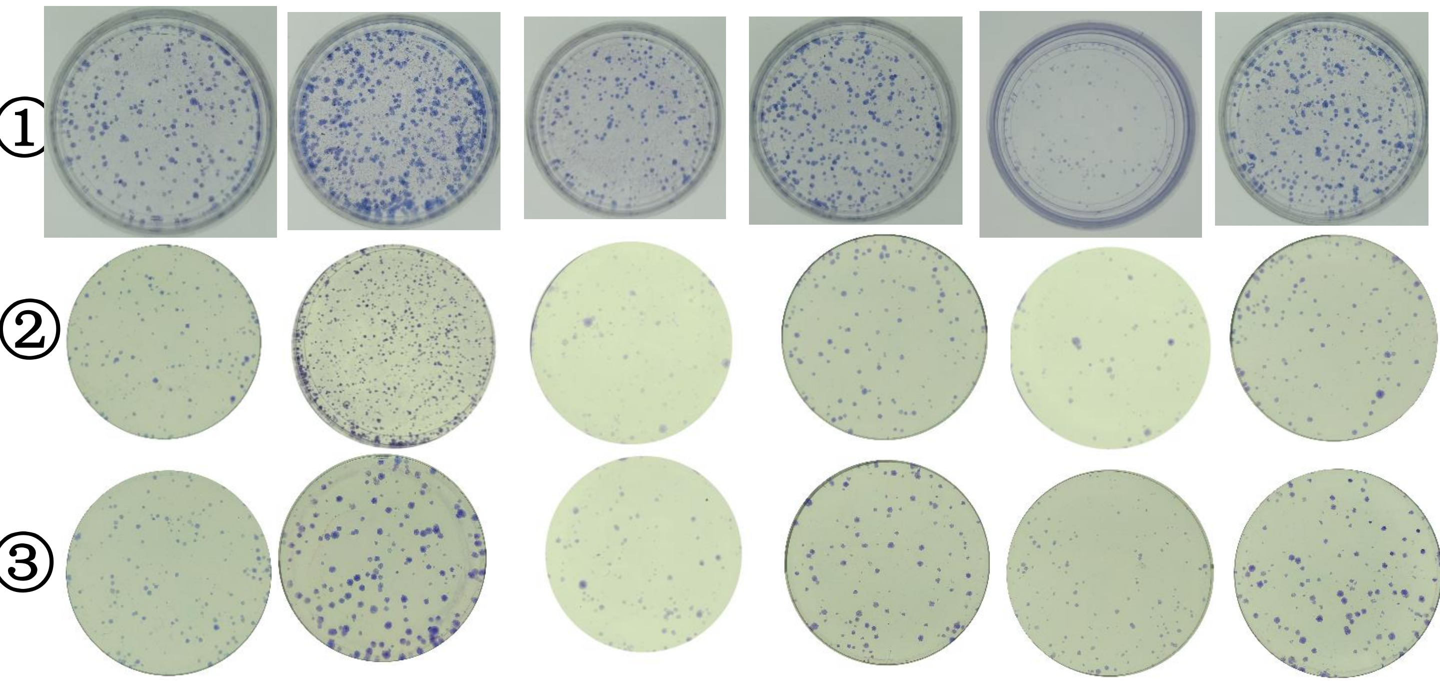

SUPD5B-468克隆形成

shcon+NC shcon+in423 sh707 #1+NC sh707 #1+in423 sh707 #2+NC sh707 #2+in423

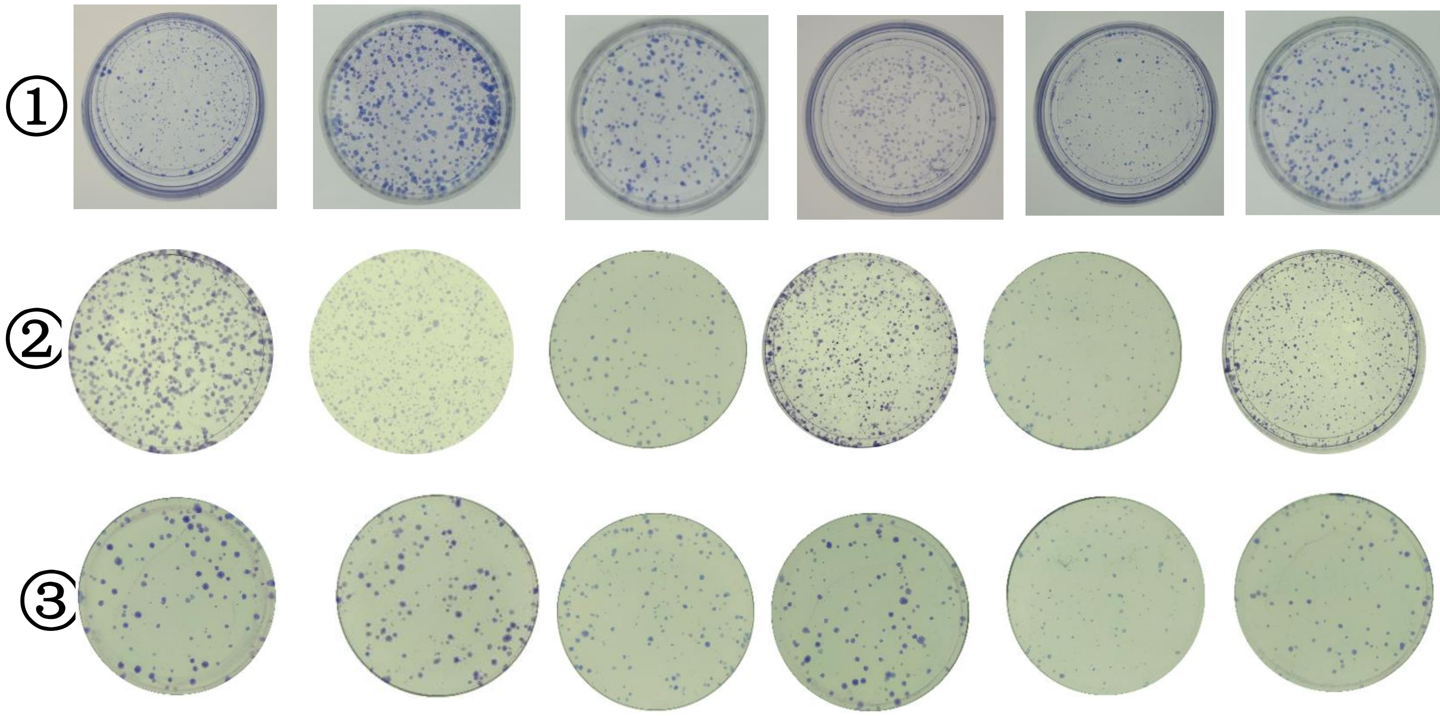

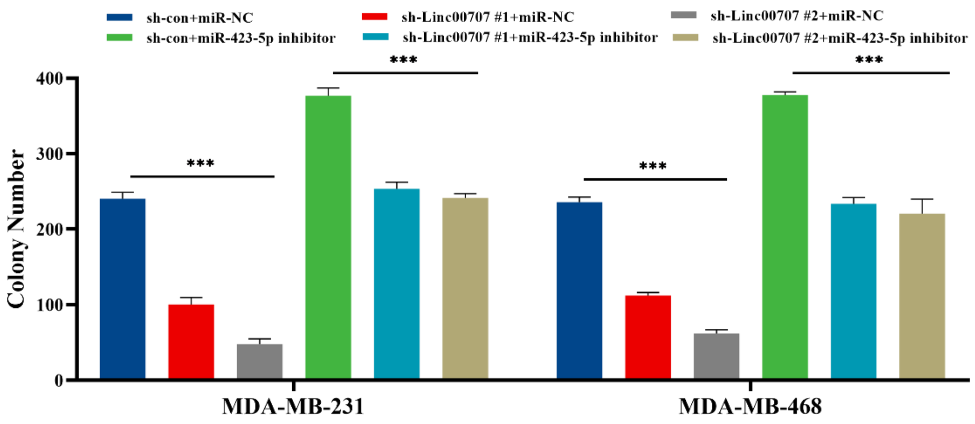

SUPD5E-231woundhealing

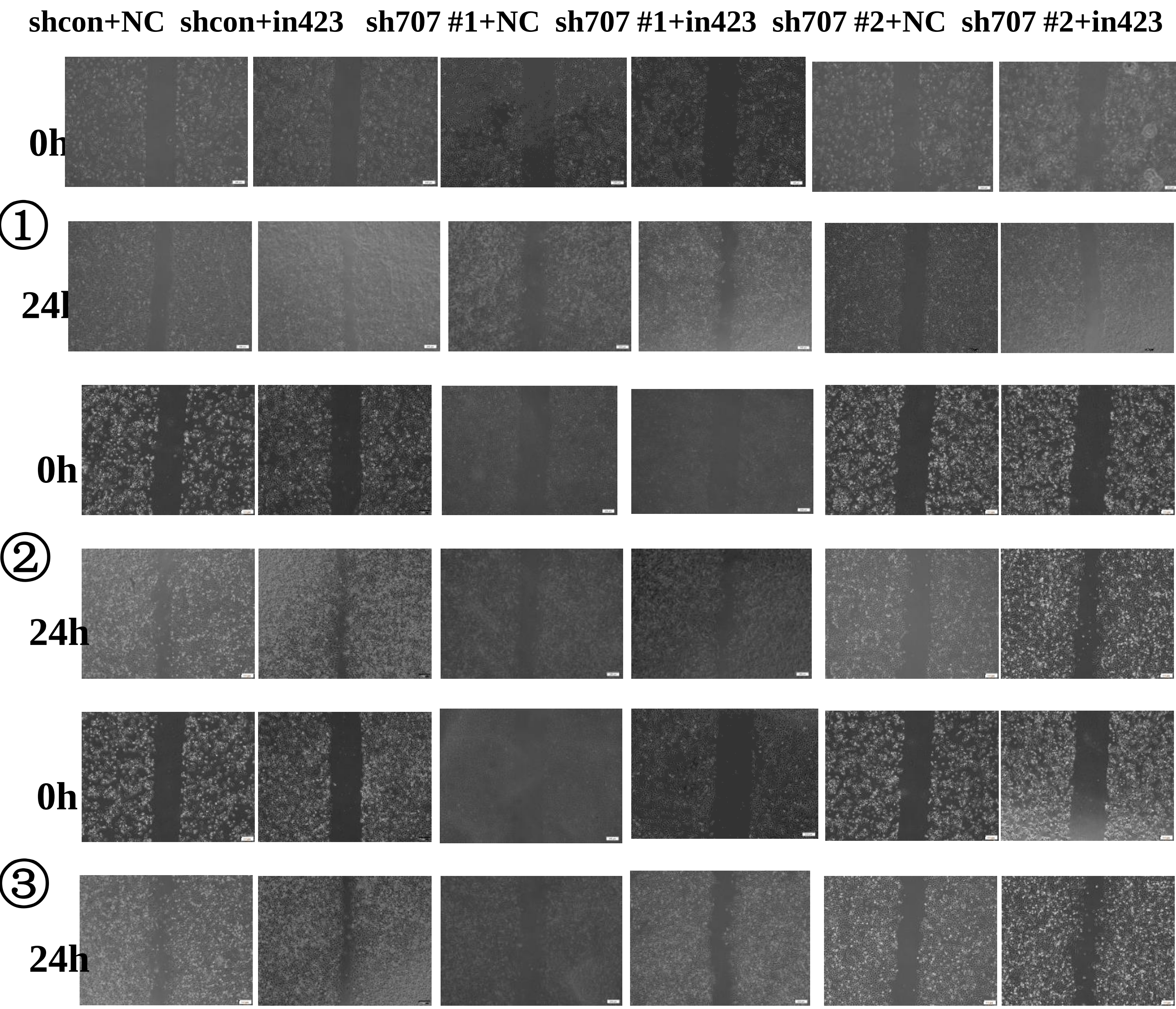

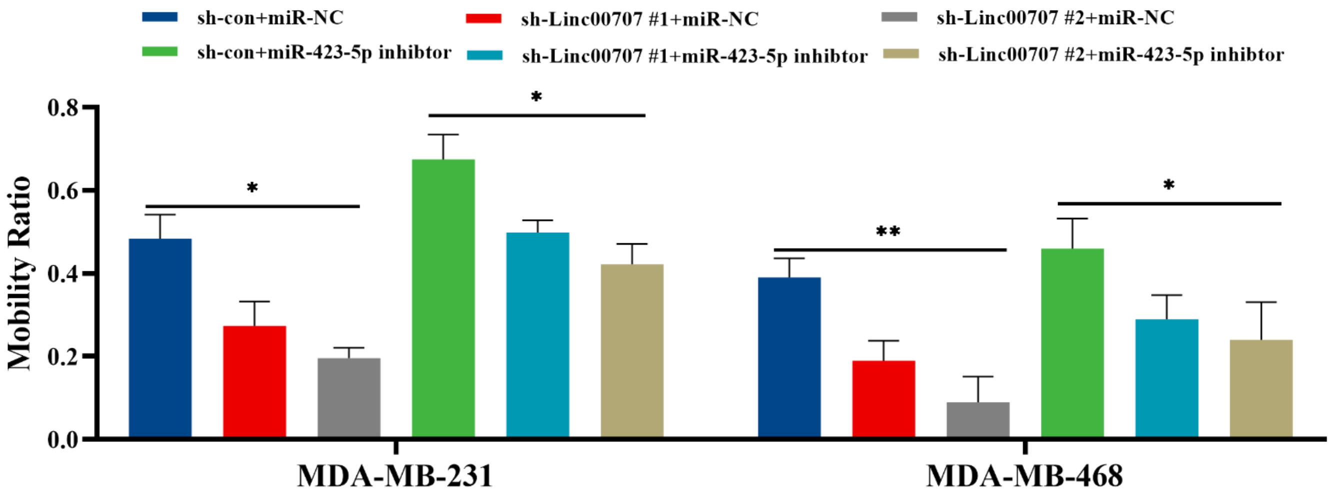

SUPD5F-468woundhealing

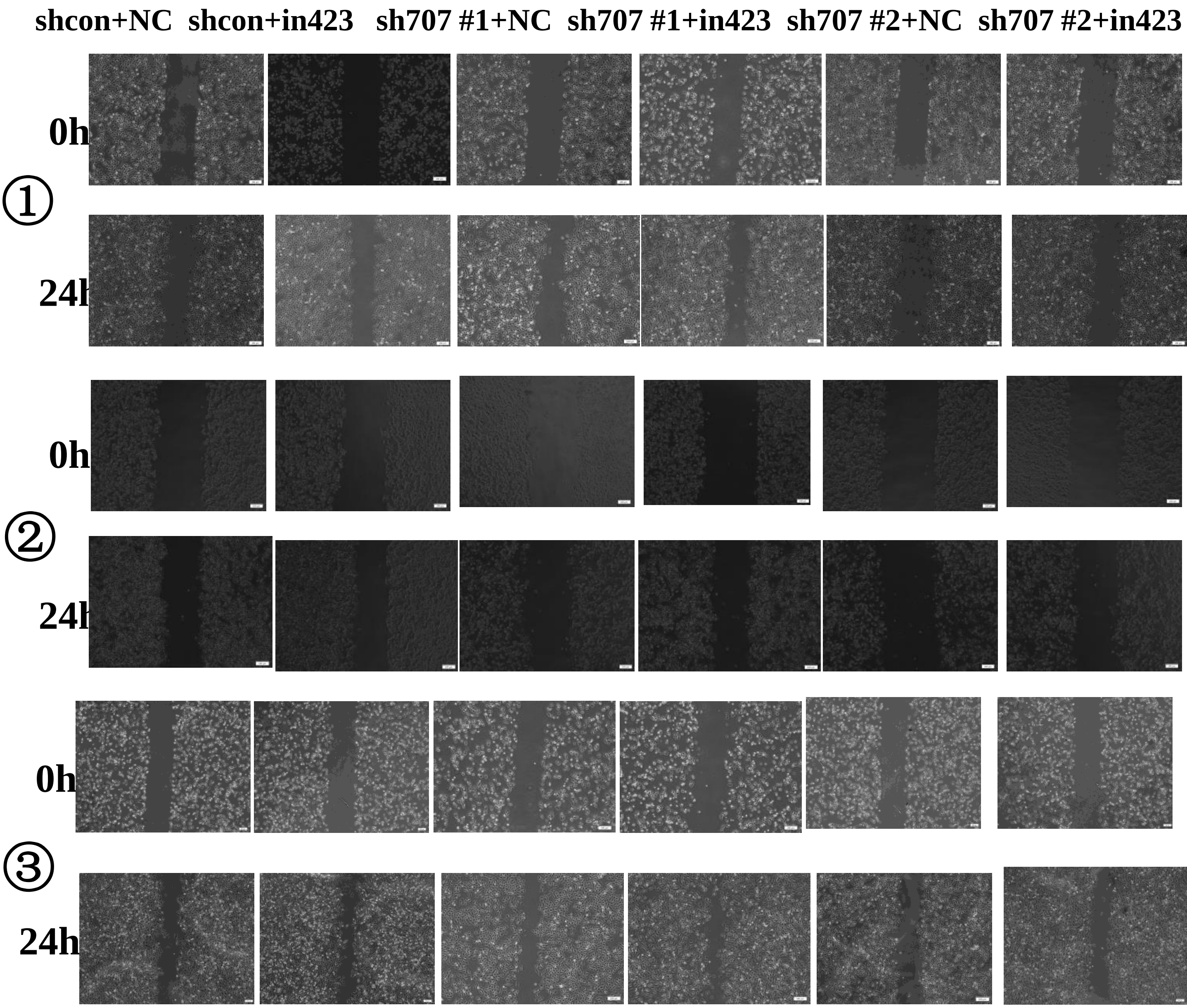

Supplement: Supplementary file 7 — Original Data File [file 41420_2024_1906_MOESM7_ESM.pdf]
